# Supplementary material for: Cardiometabolic health and physical robustness map onto distinct patterns of brain structure and neurotransmitter systems
Source: PLoS Biol. 2025 Nov 21;23(11):e3003498. doi: 10.1371/journal.pbio.3003498 (PMC12671889; doi:10.1371/journal.pbio.3003498)
Supplement: S1 File — (PDF) [file pbio.3003498.s001.pdf]

Supplementary Information

Cardiometabolic health and physical robustness map onto distinct patterns of brain structure and neurotransmitter systems

Eliana Nicolaisen-Sobesky, Somayeh Maleki Balajoo, Mostafa Mahdipour,
Agoston Mihalik, Mahnaz Olfati, Felix Hoffstaedter, Janaina Mourao-Miranda,
Masoud Tahmasian, Simon B. Eickhoff, Sarah Genon

Captions

1. Supplementary methods

1.1 Sex-specific analyses.
1.2 Comparison of latent dimensions with demographics.
1.3 Latent dimensions linking risk factors to subcortical and cerebellar volumes.

2. Supplementary results

2.1 Latent dimensions linking cardiometabolic health to raw cortical thickness and raw grey matter volume.
2.2 Latent dimensions linking cardiometabolic health to proportional cortical thickness and proportional grey matter volume.
2.3 Latent dimensions linking cardiometabolic health to corrected cortical thickness and corrected grey matter volume.
2.4 Latent dimension of cardiometabolic health and demographics.
2.5 Latent dimensions linking physical robustness to raw cortical thickness and raw grey matter volume.
2.6 Latent dimensions linking physical robustness to proportional cortical thickness and proportional grey matter volume.
2.7 Latent dimensions linking physical robustness to corrected cortical thickness and corrected grey matter volume.
2.8 Latent dimension of physical robustness and demographics.
2.9 Latent dimensions linking risk factors to subcortical and cerebellar volumes

3. Supplementary tables

Table A. Variables of risk factors used in this study.
Table B. Demographic variables used in this study.
Table C. Brain maps included in neuromaps that were compared with the latent dimension.
Table D. Statistics of the latent dimensions for raw cortical thickness.
Table E. Statistics of the latent dimensions for raw grey matter volume.
Table F. Statistics of the latent dimensions for proportional cortical thickness.
Table G. Statistics of the latent dimensions for proportional grey matter volume.
Table H. Statistics of the latent dimensions for corrected cortical thickness.
Table I. Statistics of the latent dimensions for corrected grey matter volume.
Table J. Association between the brain loadings of the latent dimension of cardiometabolic health and neuromaps.
Table K. Association between the brain loadings of the latent dimension of physical robustness and neuromaps.
Table L. Statistics of the latent dimensions for subcortical and cerebellar volumes.

4. Supplementary figures

Figure A. Distribution of risk factors. Part 1.
Figure B. Distribution of risk factors. Part 2.
Figure C. Correlation matrix of risk factors in women.
Figure D. Correlation matrix of risk factors in men.
Figure E. Latent dimension of cardiometabolic health.
Figure F. Model optimization for the latent dimension of cardiometabolic health for raw cortical thickness in the main sample.

Figure G. Model optimization for the latent dimension of cardiometabolic health for raw grey matter volume in the main sample.
Figure H. Comparison of risk factor loadings and brain loadings for the cardiometabolic health latent dimension across samples and brain structural measures.
Figure I. Loadings of the latent dimension of cardiometabolic health for raw cortical thickness in women.
Figure J. Loadings of the latent dimension of cardiometabolic health for raw cortical thickness in men.
Figure K. Loadings of the latent dimension of cardiometabolic health for raw grey matter volume in women.
Figure L. Loadings of the latent dimension of cardiometabolic health for raw grey matter volume in men.
Figure M. Loadings of the latent dimension of cardiometabolic health for proportional cortical thickness in the main sample.
Figure N. Loadings of the latent dimension of cardiometabolic health for proportional cortical thickness in women.
Figure O. Loadings of the latent dimension of cardiometabolic health for proportional cortical thickness in men.
Figure P. Loadings of the latent dimension of cardiometabolic health for proportional grey matter volume in the main sample.
Figure Q. Loadings of the latent dimension of cardiometabolic health for proportional grey matter volume in women.
Figure R. Loadings of the latent dimension of cardiometabolic health for proportional grey matter volume in men.
Figure S. Loadings of the latent dimension of cardiometabolic health for corrected cortical thickness in the main sample.
Figure T. Loadings of the latent dimension of cardiometabolic health for corrected cortical thickness in women.
Figure U. Loadings of the latent dimension of cardiometabolic health for corrected cortical thickness in men.
Figure V. Loadings of the latent dimension of cardiometabolic health for corrected grey matter volume in the main sample.
Figure W. Loadings of the latent dimension of cardiometabolic health for corrected grey matter volume in women.
Figure X. Loadings of the latent dimension of cardiometabolic health for corrected grey matter volume in men.
Figure Y. Latent dimension of physical robustness.
Figure Z. Model optimization for the latent dimension of physical robustness for raw cortical thickness in the sample of men.
Figure AA. Model optimization for the latent dimension of physical robustness for raw grey matter volume in the main sample.
Figure AB. Loadings of the latent dimension of physical robustness for raw cortical thickness in women.
Figure AC. Loadings of the latent dimension of physical robustness for raw grey matter volume in women.
Figure AD. Loadings of the latent dimension of physical robustness for raw grey matter volume in men.
Figure AE. Comparison of risk factor loadings and brain loadings for the physical robustness latent dimension across samples and brain structural measures.
Figure AF. Loadings of the latent dimension of physical robustness for proportional cortical thickness in men.
Figure AG. Loadings of the latent dimension of physical robustness for proportional grey matter volume in the main sample.
Figure AH. Loadings of the latent dimension of physical robustness for proportional grey matter volume in women.
Figure AI. Loadings of the latent dimension of physical robustness for proportional grey matter volume in men.
Figure AJ. Loadings of the latent dimension of physical robustness for corrected cortical thickness in men.
Figure AK. Loadings of the latent dimension of physical robustness for corrected cortical thickness in women.
Figure AL. Loadings of the latent dimension of physical robustness for corrected grey matter volume in the main sample.
Figure AM. Loadings of the latent dimension of physical robustness for corrected grey matter volume in women.
Figure AN. Loadings of the latent dimension of physical robustness for corrected grey matter volume in men.
Figure AO. Association of brain structural loadings with brain maps for the latent dimension of cardiometabolic health.
Figure AP. Association of brain structural loadings with brain maps for the latent dimension of physical robustness.
Figure AQ. Loadings of the first latent dimension linking risk factors to subcortical and cerebellar volumes.
Figure AR. Loadings of the second latent dimension linking risk factors to subcortical and cerebellar volumes.
Figure AS. Loadings of the third latent dimension linking risk factors to subcortical and cerebellar volumes.
Figure AT. Loadings of the fourth latent dimension linking risk factors to subcortical and cerebellar volumes.
Figure AU. Loadings of the fifth latent dimension linking risk factors to subcortical and cerebellar volumes.

Figure AV. Comparison of risk factor loadings between latent dimensions yielded with subcortex-cerebellum and with CT and GMV in cortex.

1. Supplementary methods

1.1 Sex-specific analyses

In the main paper, we present analyses in a sample including both, men and women (the main/mixed sample). In addition, we performed sex-specific analyses because previous works have shown sex differences in the association between brain structure and risk factors (1,2) and also in order to avoid sex-bias in the results (3,4). The existence of a sex bias in human neuroscience is recognized, with the consequence that much of our knowledge in the field is based on men, and do not or might not generalize to women (5). To perform the sex-specific analyses, we run our pipeline independently in the subsample of women and in the subsample of men.

1.2 Comparison of latent dimensions with demographics

To assess the role of demographic variables in the latent dimensions, we performed Spearman correlation between the risk factors scores and household income, employment status, and education (Table B). Multiple comparisons were corrected using the Bonferroni method.

1.3 Latent dimensions linking risk factors to subcortical and cerebellar volumes

Using the same framework described in the main paper for the analyses with cortical data (CT and GMV), we also analyzed latent dimensions linking the same set of risk factors with subcortical and cerebellar volumes in the main sample. Subcortical and cerebellar volumes were estimated with the FMRIB pipeline and parceled with the Harvard-Oxford and Diedrichsen atlases (6)**.** Age, site and sex were regressed out avoiding data leakage (7).

2. Supplementary results

2.1 Latent dimensions linking cardiometabolic health to raw cortical thickness and raw grey matter volume

When analyzed in association with raw CT, the latent dimension of cardiometabolic health was significant in the sample of women (Figure I, Table D) and in the sample of men (Figure J, Table J).

When analyzed in association with raw GMV, the latent dimension of cardiometabolic health was significant in the sample of women (Figure K, Table E) and in the sample of men (Figure L, Table E).

Interestingly, the risk factors loadings associated with cardiometabolic health were similar when yielded with raw CT or with raw GMV, while the CT and GMV loadings were not significantly associated (Figure H).

2.2 Latent dimensions linking cardiometabolic health to proportional cortical thickness and proportional grey matter volume

When analyzed in association with proportional CT, the latent dimension of cardiometabolic health was significant in the main/mixed sample (Figure M, Table F), as well as in the sample of women (Figure N, Table F) and in the sample of men (Figure O, Table F).

When analyzed in association with proportional GMV, the latent dimension of cardiometabolic health was significant in the main/mixed sample (Figure P, Table G), as well as in the sample of women (Figure Q, Table G) and in the sample of men (Figure R, Table G).

Interestingly, the risk factors loadings associated with cardiometabolic health were similar when yielded with proportional CT or with proportional GMV, while the CT and GMV loadings were not significantly associated (Figure H).

2.3 Latent dimensions linking cardiometabolic health to corrected cortical thickness and corrected grey matter volume

When analyzed in association with corrected CT, the latent dimension of cardiometabolic health was significant in the main/mixed sample (Figure S, Table H), as well as in the sample of women (Figure T, Table H) and in the sample of men (Figure U, Table H).

When analyzed in association with corrected GMV, the latent dimension of cardiometabolic health was significant in the main/mixed sample (Figure V, Table I), as well as in the sample of women (Figure W, Table I) and in the sample of men (Figure X, Table I).

Interestingly, the risk factors loadings associated with cardiometabolic health were similar when yielded with corrected CT or with corrected GMV, while the CT and GMV loadings were not significantly associated (Figure H).

2.4 Latent dimension of cardiometabolic health and demographics

Our results showed that qualifications (education) and household income were significantly associated with the risk factor scores of the latent dimension of cardiometabolic health when yielded with raw CT (qualifications: $r = 0.15$, $p < 0.001$; income: $r = 0.07$, $p < 0.001$) and raw GMV (qualifications: $r = 0.13$, $p < 0.001$; income: $r = 0.06$, $p < 0.001$) in the main/mixed subsample. This indicates that individuals who score high on the positive pole of risk factors (that are characterized for example by higher physical activity) and hence who score very low on the negative pole of risk factors (that are characterized for example with lower BMI and body size) are associated with higher qualifications and higher household income.

Analyses could not be performed for ethnic background and some employment dummy variables (those corresponding to values 3-7) because of imbalanced distribution.

2.5 Latent dimensions linking physical robustness to raw cortical thickness and raw grey matter volume

When analyzed in association with raw CT, the latent dimension of physical robustness was significant in the sample of men (Figure 4 in main paper, Table D). However, in the sample of women it was significant in only one split ($r = 0.103$, $p = 0.01$) out of five ($r_{range} = 0.013\text{-}0.027$, $p_{range} = 0.384\text{-}0.999$). Considering that, and that the loadings were unstable for most of the risk factors loadings and brain regions in the sample of women (Figure AB), for the latent dimension of physical robustness and cortical thickness we focus on the sample of men.

When analyzed in association with raw GMV, the latent dimension of physical robustness was significant in the sample of women (Figure AC, Table E) and in the sample of men (Figure AD, Table E).

Interestingly, the risk factors loadings associated with physical robustness were similar when yielded with raw CT or with raw GMV, while the CT and GMV loadings were not significantly associated (Figure AE).

2.6 Latent dimensions linking physical robustness to proportional cortical thickness and proportional grey matter volume

When analyzed in association with proportional CT, the latent dimension of physical robustness was significant in the sample of men (Figure AF, Table F). However, in the sample of women it was not significant (r_{range} = 0.003-0.060, p_{range} = 0.220-0.999). For this reason, for the latent dimension of physical robustness and cortical thickness we focus on the sample of men.

When analyzed in association with proportional GMV, the latent dimension of physical robustness was significant in the main/mixed sample (Figure AG, Table G), as well as in the sample of women (Figure AH, Table G) and in the sample of men (Figure AI, Table G).

Interestingly, the risk factors loadings associated with physical robustness were similar when yielded with proportional CT or with proportional GMV, while the CT and GMV loadings were not significantly associated (Figure AE).

2.7 Latent dimensions linking physical robustness to corrected cortical thickness and corrected grey matter volume

When analyzed in association with corrected CT, the latent dimension of physical robustness was significant in the sample of men (Figure AJ, Table H). However, in the sample of women it was significant in only one split (r = 0.108, p = 0.01) out of five (r_{range} = 0.003-0.047, p_{range} = 0.469-0.999). Considering that, and that the loadings were quite unstable (Figure AK), for the latent dimension of physical robustness and cortical thickness we focus on the sample of men.

When analyzed in association with corrected GMV, the latent dimension of physical robustness was significant in the main/mixed sample (Figure AL, Table I), as well as in the sample of women (Figure AM, Table I) and in the sample of men (Figure AN, Table I).

Interestingly, the risk factors loadings associated with physical robustness were similar when yielded with corrected CT or with corrected GMV, while the CT and GMV loadings were not significantly associated (Figure AE).

2.8 Latent dimension of physical robustness and demographics

Our results showed household income was significantly associated with the risk factor scores of the latent dimension of physical robustness when yielded with raw CT in the sample of men ($r = 0.05$, $p = 0.039$) and with raw GMV in the main/mixed sample ($r = 0.08$, $p < 0.001$). Also, qualifications (education) was associated with the risk factor scores when yielded with raw GMV in the main/mixed sample ($r = 0.10$, $p < 0.001$).

Analyses could not be performed for ethnic background and some employment dummy variables (those corresponding to values 3-7) because of imbalanced distribution.

2.9 Latent dimensions linking risk factors to subcortical and cerebellar volumes

The analysis of subcortical and cerebellar data yielded five significant latent dimensions (Figures AQ, AR, AS, AT and AU, Table L). Of those, the risk factor loadings of the first subcortex-cerebellum latent dimension showed significant associations with the loadings of both, the latent dimension of cardiometabolic health and the latent dimension of physical robustness yielded with the cortical data (CT and GMV) (Figure AV). The second latent dimension yielded with subcortex-cerebellum also showed significant associations with the latent dimension of cardiometabolic health. In other words, this first latent dimension that explains interindividual variability in non-cortical regions seems to combine both cardiometabolic health and physical robustness factors. It relates to variability across a wide range of non-cortical regions, such as the amygdala, the hippocampus, the thalamus and several cerebellar regions, but to a lesser extend in basal ganglia regions. This suggests that the set of factors that explain variability in subcortical regions in an aging population may be importantly more complex than the set of factors that relate cortical interindividual variability. Accordingly, we would encourage future studies to further specifically investigate factors that may affect subcortical structures across the lifespan, instead of considering cortical and subcortical regions globally.

3. Supplementary tables

Table A. Variables of risk factors used in this study.

UDI	Label	Category	Modified or reversed
46-2.0	Hand grip strength (left)	Hand grip strength	
47-2.0	Hand grip strength (right)	Hand grip strength	
48-2.0	Waist circumference	Body size measures	
49-2.0	Hip circumference	Body size measures	
102-0.0	Pulse rate, automated reading	Blood pressure	
135-0.0	Number of self-reported non-cancer illnesses	Medical conditions	
136-0.0	Number of operations, self-reported	Operations	
137-0.0	Number of treatments/medications taken	Medications	
864-2.0	Number of days/weeks walked 10+ minutes	Physical activity	
884-2.0	Number of days/weeks of moderate physical activity 10+ minutes	Physical activity	
904-2.0	Number of days/weeks of vigorous physical activity 10+ minutes	Physical activity	

924-2.0	Usual walking pace	Physical activity	
943-2.0	Frequency of stair climbing in last 4 weeks	Physical activity	
1070-2.0	Time spent watching television (TV)	Physical activity	Modified (-10 replaced by 0)
1080-2.0	Time spent using computer	Physical activity	Modified (-10 replaced by 0)
1090-2.0	Time spent driving	Physical activity	Modified (-10 replaced by 0)
1160-2.0	Sleep duration	Sleep	
1170-2.0	Getting up in morning	Sleep	
1190-2.0	Nap during day	Sleep	
1200-2.0	Sleeplessness / insomnia	Sleep	
1249-2.0	Past tobacco smoking	Smoking	Reversed ([1,2,3,4] replaced by [14,13,12,11])
1289-2.0	Cooked vegetable intake	Diet	Modified (-10 replaced by 0)
1299-2.0	Salad / raw vegetable intake	Diet	Modified (-10 replaced by 0)
1309-2.0	Fresh fruit intake	Diet	Modified (-10 replaced by 0)

1319-2.0	Dried fruit intake	Diet	Modified (-10 replaced by 0)
1329-2.0	Oily fish intake	Diet	
1339-2.0	Non-oily fish intake	Diet	
1349-2.0	Processed meat intake	Diet	
1359-2.0	Poultry intake	Diet	
1369-2.0	Beef intake	Diet	
1379-2.0	Lamb/mutton intake	Diet	
1389-2.0	Pork intake	Diet	
1408-2.0	Cheese intake	Diet	
1438-2.0	Bread intake	Diet	Modified (-10 replaced by 0)
1458-2.0	Cereal intake	Diet	Modified (-10 replaced by 0)
1478-2.0	Salt added to food	Diet	
1488-2.0	Tea intake	Diet	Modified (-10 replaced by 0)
1498-2.0	Coffee intake	Diet	Modified (-10 replaced by 0)
1528-2.0	Water intake	Diet	Modified (-10 replaced by 0)

1538-2.0	Major dietary changes in the last 5 years	Diet	Modified (2 replaced by 1)
1548-2.0	Variation in diet	Diet	
1558-2.0	Alcohol intake frequency.	Alcohol	Reversed ([1,2,3,4,5,6] replaced by [16,15,14,13,12,11])
1618-2.0	Alcohol usually taken with meals	Alcohol	Modified (1, 0, -6 replaced by 12,10,22 respectively)
1628-2.0	Alcohol intake versus 10 years previously	Alcohol	Reversed ([1,2,3] replaced by [12,11,10])
2178-2.0	Overall health rating	General health	Reversed ([1,2,3,4] replaced by [14,13,12,11])
2306-2.0	Weight change compared with 1 year ago	General health	Modified ([0,2,3] replaced by [11, 12, 10])
2492-2.0	Taking other prescription medications	Medication	
4079-0.0	Diastolic blood pressure, automated reading	Blood pressure	
4080-0.0	Systolic blood pressure, automated reading	Blood pressure	
20116-2.0	Smoking status	Smoking	
20160-2.0	Ever smoked	Smoking	

21001-2.0	Body mass index (BMI)	Body size measures	
21003-2.0	Age when attended assessment centre	Reception	
23099-2.0	Body fat percentage	Body composition by impedance	
23100-2.0	Whole body fat mass	Body composition by impedance	
23101-2.0	Whole body fat-free mass	Body composition by impedance	
23102-2.0	Whole body water mass	Body composition by impedance	
23105-2.0	Basal metabolic rate	Body composition by impedance	
23106-2.0	Impedance of whole body	Body composition by impedance	
24003-0.0	Nitrogen dioxide air pollution; 2010	Residential air pollution	
24004-0.0	Nitrogen oxides air pollution; 2010	Residential air pollution	
24005-0.0	Particulate matter air pollution (pm10); 2010	Residential air pollution	

24006-0.0	Particulate matter air pollution (pm2.5); 2010	Residential air pollution	
24007-0.0	Particulate matter air pollution (pm2.5) absorbance; 2010	Residential air pollution	
24008-0.0	Particulate matter air pollution 2.5-10um; 2010	Residential air pollution	
24011-0.0	Traffic intensity on the nearest major road	Residential air pollution	
24018-0.0	Nitrogen dioxide air pollution; 2007	Residential air pollution	
24019-0.0	Particulate matter air pollution (pm10); 2007	Residential air pollution	
46-2.0	Hand grip strength (left)	Hand grip strength	
54-2.0	UK Biobank assessment centre (site)	Reception	Dummy
1428-2.0	Spread type	Diet	Dummy

Some scores were reversed to facilitate interpretability, represented as dummy variables, or modified to create ordinal variables.

Table B. Demographic variables used in this study.

UDI	Label	Category	Modified or reversed
738-2.0	Average total household income before tax	Household	
21000-0.0	Ethnic background	Ethnicity	
6138-2.*	Qualifications	Education	Modified and reversed: [1,2,3,4,5,6] were replaced by [16,15,14,14,None,15]. Original value 5 was replaced by None in order to create ordinal variable.
6142-2.*	Current employment status	Employment	Dummy

Table C. Brain maps included in neuromaps that were compared with the latent dimension.

('abagen', 'genepc1', 'fsaverage', '10k')	PC1 of genes in the Allen Human Brain Atlas
('aghourian2017', 'feobv', 'MNI152', '1mm')	PET tracer binding (SUVR) to VAChT (acetylcholine transporter)
('alarkurtti2015', 'raclopride', 'MNI152', '3mm')	PET tracer binding (BPnd) to D2 (dopamine receptor)
('bedard2019', 'feobv', 'MNI152', '1mm')	PET tracer binding (SUVR) to VAChT (acetylcholine transporter)
('beliveau2017', 'az10419369', 'fsaverage', '164k')	PET tracer binding (Bmax) to 5-HT1b (serotonin receptor)
('beliveau2017', 'cimbi36', 'fsaverage', '164k')	PET tracer binding (Bmax) to 5-HT2a (serotonin receptor)
('beliveau2017', 'cumi101', 'fsaverage', '164k')	PET tracer binding (Bmax) to 5-HT1a (serotonin receptor)
('beliveau2017', 'dasb', 'fsaverage', '164k')	PET tracer binding (Bmax) to 5-HTT (serotonin transporter)
('beliveau2017', 'sb207145', 'fsaverage', '164k')	PET tracer binding (Bmax) to 5-HT4 (serotonin receptor)
('ding2010', 'mrb', 'MNI152', '1mm')	PET tracer binding (BPnd) to NET (norepinephrine transporter)
('dubois2015', 'abp688', 'MNI152', '1mm')	PET tracer binding (BPnd) to mGluR5 (glutamate receptor)
('dukart2018', 'flumazenil', 'MNI152', '3mm')	PET tracer binding (BPnd) to GABAa (gaba receptor)
('dukart2018', 'fpcit', 'MNI152', '3mm')	SPECT tracer binding (SUVR) to DAT (dopamine transporter)
('fazio2016', 'madam', 'MNI152', '3mm')	PET tracer binding (BPnd) to 5-HTT (serotonin transporter)
('finnema2016', 'ucbj', 'MNI152', '1mm')	PET tracer binding (BPnd) to SV2A (synaptic vesicle glycoprotein 2A, a synapse marker)

('gallezot2010', 'p943', 'MNI152', '1mm')	PET tracer binding (BPnd) to 5-HT1b (serotonin receptor)
('gallezot2017', 'gsk189254', 'MNI152', '1mm')	PET tracer binding (Vt) to H3 (histamine receptor)
('galovic2021', 'ge179', 'MNI152', '1mm')	PET tracer binding (Vt) to NMDA (glutamate receptor)
('hcps1200', 'megalpha', 'fsLR', '4k')	MEG alpha (8-12 Hz) power distribution from the Human Connectome Project S1200 release
('hcps1200', 'megbeta', 'fsLR', '4k')	MEG beta (15-29 Hz) power distribution from the Human Connectome Project S1200 release
('hcps1200', 'megdelta', 'fsLR', '4k')	MEG delta (2-4 Hz) power distribution from the Human Connectome Project S1200 release
('hcps1200', 'meggamma1', 'fsLR', '4k')	MEG low gamma (30-59 Hz) power distribution from the Human Connectome Project S1200 release
('hcps1200', 'meggamma2', 'fsLR', '4k')	MEG high gamma (60-90 Hz) power distribution from the Human Connectome Project S1200 release
('hcps1200', 'megtheta', 'fsLR', '4k')	MEG theta (5-7 Hz) power distribution from the Human Connectome Project S1200 release
('hcps1200', 'megtimescale', 'fsLR', '4k')	MEG intrinsic timescale from the Human Connectome Project S1200 release
('hcps1200', 'myelinmap', 'fsLR', '32k')	MRI T1w/T2w ratio from the Human Connectome Project S1200 release
('hcps1200', 'thickness', 'fsLR', '32k')	MRI cortical thickness from the Human Connectome Project S1200 release
('hesse2017', 'methylreboxetine', 'MNI152', '3mm')	PET tracer binding (BPnd) to NET (norepinephrine transporter)
('hillmer2016', 'flubatine', 'MNI152', '1mm')	PET tracer binding (Vt) to a4b2 (acetylcholine receptor)

('jaworska2020', 'fallypride', 'MNI152', '1mm')	PET tracer binding (BPnd) to D2 (dopamine receptor)
('kaller2017', 'sch23390', 'MNI152', '3mm')	PET tracer binding (BPnd) to D1 (dopamine receptor)
('kantonen2020', 'carfentanil', 'MNI152', '3mm')	PET tracer binding (BPnd) to MOR (mu-opioid receptor)
('kim2020', 'ps13', 'MNI152', '2mm')	PET tracer binding (Vt) to COX-1 (cyclooxygenase-1)
('laurikainen2018', 'fmpepd2', 'MNI152', '1mm')	PET tracer binding (Vt) to CB1 (cannabinoid receptor)
('lois2018', 'pbr28', 'MNI152', '2mm')	PET tracer binding (SUVR) to TSPO (translocator protein)
('lukow2022', 'ro154513', 'MNI152', '2mm')	PET tracer binding (BPnd) to GABAa receptor, alpha5 subunit
('malen2022', 'raclopride', 'MNI152', '2mm')	PET tracer binding (BPnd) to D2 (dopamine receptor)
('margulies2016', 'fcgradient01', 'fsLR', '32k')	Diffusion map embedding gradient 1 of group-averaged functional connectivity
('margulies2016', 'fcgradient02', 'fsLR', '32k')	Diffusion map embedding gradient 2 of group-averaged functional connectivity
('margulies2016', 'fcgradient03', 'fsLR', '32k')	Diffusion map embedding gradient 3 of group-averaged functional connectivity
('margulies2016', 'fcgradient04', 'fsLR', '32k')	Diffusion map embedding gradient 4 of group-averaged functional connectivity
('margulies2016', 'fcgradient05', 'fsLR', '32k')	Diffusion map embedding gradient 5 of group-averaged functional connectivity
('margulies2016', 'fcgradient06', 'fsLR', '32k')	Diffusion map embedding gradient 6 of group-averaged functional connectivity
('margulies2016', 'fcgradient07', 'fsLR', '32k')	Diffusion map embedding gradient 7 of group-averaged functional connectivity

('margulies2016', 'fcgradient08', 'fsLR', '32k')	Diffusion map embedding gradient 8 of group-averaged functional connectivity
('margulies2016', 'fcgradient09', 'fsLR', '32k')	Diffusion map embedding gradient 9 of group-averaged functional connectivity
('margulies2016', 'fcgradient10', 'fsLR', '32k')	Diffusion map embedding gradient 10 of group-averaged functional connectivity
('mueller2013', 'intersubjvar', 'fsLR', '164k')	Intersubject variability of resting-state functional connectivity.
('naganawa2020', 'lsn3172176', 'MNI152', '1mm')	PET tracer binding (BPnd) to M1 (acetylcholine receptor)
('neurosynth', 'cogpc1', 'MNI152', '2mm')	PC1 of Neurosynth terms in the Cognitive Atlas (123 terms total)
('norgaard2021', 'flumazenil', 'fsaverage', '164k')	PET and autoradiography informed GABAa benzodiazepine binding-site density (Bmax
('normandin2015', 'omar', 'MNI152', '1mm')	PET tracer binding (Vt) to CB1 (cannabinoid receptor)
('radnakrishnan2018', 'gsk215083', 'MNI152', '1mm')	PET tracer binding (BPnd) to 5-HT6 (serotonin receptor)
('raichle', 'cbf', 'fsLR', '164k')	Cerebral blood flow
('raichle', 'cbv', 'fsLR', '164k')	Cerebral blood volume
('raichle', 'cmr02', 'fsLR', '164k')	Oxygen metabolism
('raichle', 'cmrglc', 'fsLR', '164k')	Glucose metabolism
('reardon2018', 'scalinghcp', 'civet', '41k')	Cortical areal scaling during development from the HCP dataset (S1200 release)
('reardon2018', 'scalingnih', 'civet', '41k')	Cortical areal scaling during development from the NIH dataset

('reardon2018', 'scalingpnc', 'civet', '41k')	Cortical areal scaling during development from the PNC dataset
('rosaneto', 'abp688', 'MNI152', '1mm')	PET tracer binding (BPnd) to mGluR5 (glutamate receptor)
('sandiego2015', 'flb457', 'MNI152', '1mm')	PET tracer binding (BPnd) to D2 (dopamine receptor)
('sasaki2012', 'fepe2i', 'MNI152', '1mm')	PET tracer binding (BPnd) to DAT (dopamine transporter)
('satterthwaite2014', 'meancbf', 'MNI152', '1mm')	Cerebral blood flow
('savli2012', 'altanserin', 'MNI152', '3mm')	PET tracer binding (BPnd) to 5-HT2a (serotonin receptor)
('savli2012', 'dasb', 'MNI152', '3mm')	PET tracer binding (BPnd) to 5-HTT (serotonin transporter)
('savli2012', 'p943', 'MNI152', '3mm')	PET tracer binding (BPnd) to 5-HT1b (serotonin receptor)
('savli2012', 'way100635', 'MNI152', '3mm')	PET tracer binding (BPnd) to 5-HT1a (serotonin receptor)
('smart2019', 'abp688', 'MNI152', '1mm')	PET tracer binding (BPnd) to mGluR5 (glutamate receptor)
('smith2017', 'flb457', 'MNI152', '1mm')	PET tracer binding (BPnd) to D2 (dopamine receptor)
('sydnor2021', 'SAaxis', 'fsLR', '32k')	Sensory-association mean rank axis
('tuominen', 'feobv', 'MNI152', '2mm')	PET tracer binding (SUVR) to VAChT (acetylcholine transporter)
('turtonen2020', 'carfentanil', 'MNI152', '1mm')	PET tracer binding (BPnd) to MOR (mu-opioid receptor)
('vijay2018', 'ly2795050', 'MNI152', '2mm')	PET tracer binding (Vt) to KOR (kappa-opioid receptor)
('wey2016', 'martinostat', 'MNI152', '2mm')	PET tracer binding (SUVR) to class 1 HDAC isoforms 1, 2, and 3 (histone deacetylase)
('xu2020', 'FChomology', 'fsLR', '32k')	Cross-species functional homology

('xu2020', 'evoexp', 'fsLR', '32k')	Evolutionary cortical expansion

Table D. Statistics of the latent dimensions for raw cortical thickness.

	Main/Mixed sample						Women				Men					
	1st LD		2nd LD		3rd LD		1st LD		2nd LD		1st LD		2nd LD		3rd LD	
	Cardiomeatbolic health		Physical robustness				Cardiomeatbolic health		Physical robustness		Cardiomeatbolic health		Physical robustness			
Split	r	p-value	r	p-value	r	p-value	r	p-value	r	p-value	r	p-value	r	p-value	r	p-value
1	0.34	**0.005**	0.10	**0.005**	0.10	**0.005**	0.29	**0.005**	0.10	**0.010**	0.31	**0.005**	0.17	**0.005**	0.15	**0.005**
2	0.32	**0.005**	0.09	**0.005**	0.02	>0.999	0.31	**0.005**	0.03	>0.999	0.34	**0.005**	0.12	**0.01**	0.05	0.384
3	0.30	**0.005**	0.11	**0.005**	0.10	**0.005**	0.29	**0.005**	0.03	>0.999	0.33	**0.005**	0.13	**0.005**	-0.001	>0.999
4	0.31	**0.005**	0.12	**0.005**	0.09	**0.010**	0.30	**0.005**	0.01	>0.999	0.32	**0.005**	0.19	**0.005**	0.09	**0.035**
5	0.30	**0.005**	0.10	**0.005**	0.08	**0.005**	0.25	**0.005**	0.05	0.384	0.33	**0.005**	0.18	**0.005**	0.11	**0.015**

p-values are corrected using the Bonferroni method over 5 comparisons; significant p-values are in bold; r: Pearson’s correlation coefficient

Table E. Statistics of the latent dimensions for raw grey matter volume.

	Main/Mixed sample				Women				Men			
	1st LD		2nd LD		1st LD		2nd LD		1st LD		2nd LD	
	Physical robustness		Cardiomeatbolic health		Physical robustness		Cardiomeatbolic health		Physical robustness		Cardiomeatbolic health	
Split	r	p-value	r	p-value	r	p-value	r	p-value	r	p-value	r	p-value
1	0.27	**0.005**	0.20	**0.005**	0.21	**0.005**	0.17	**0.005**	0.29	**0.005**	0.11	**0.015**
2	0.27	**0.005**	0.16	**0.005**	0.23	**0.005**	0.20	**0.005**	0.27	**0.005**	0.15	**0.005**
3	0.27	**0.005**	0.20	**0.005**	0.28	**0.005**	0.12	**0.010**	0.28	**0.005**	0.17	**0.005**
4	0.27	**0.005**	0.17	**0.005**	0.24	**0.005**	0.15	**0.005**	0.26	**0.005**	0.12	**0.010**
5	0.25	**0.005**	0.20	**0.005**	0.25	**0.005**	0.16	**0.005**	0.25	**0.005**	0.14	**0.005**

p-values are corrected using the Bonferroni method over 5 comparisons; significant p-values are in bold; r: Pearson's correlation coefficient

Table F. Statistics of the latent dimensions for proportional cortical thickness.

	Main/Mixed sample						Women		Men			
	1st LD		2nd LD		3rd LD		1st LD		1st LD		2nd LD	
	Cardiomeatbolic health		Physical robustness				Cardiomeatbolic health		Cardiomeatbolic health		Physical robustness	
Split	r	p-value	r	p-value	r	p-value	r	p-value	r	p-value	r	p-value
1	0.34	**0.005**	0.11	**0.005**	0.09	**0.005**	0.29	**0.005**	0.31	**0.005**	0.17	**0.005**
2	0.33	**0.005**	0.06	**0.05**	0.02	>0.99	0.32	**0.005**	0.34	**0.005**	0.11	**0.005**
3	0.30	**0.005**	0.11	**0.005**	0.08	**0.005**	0.29	**0.005**	0.32	**0.005**	0.13	**0.005**
4	0.32	**0.005**	0.12	**0.005**	0.11	**0.005**	0.29	**0.005**	0.31	**0.005**	0.17	**0.005**
5	0.30	**0.005**	0.09	**0.005**	0.06	0.070	0.26	**0.005**	0.32	**0.005**	0.16	**0.005**

p-values are corrected using the Bonferroni method over 5 comparisons; significant p-values are in bold; r: Pearson's correlation coefficient

Table G. Statistics of the latent dimensions for proportional grey matter volume.

	Main/Mixed sample						Women				Men					
	1st LD		2nd LD		3rd LD		1st LD		2nd LD		1st LD		2nd LD		3rd LD	
	Cardiomeatbolic health		Physical robustness				Cardiomeatbolic health		Physical robustness		Cardiomeatbolic health		Physical robustness			
Split	r	p-value	r	p-value	r	p-value	r	p-value	r	p-value	r	p-value	r	p-value	r	p-value
1	0.18	**0.005**	0.17	**0.005**	0.07	**0.035**	0.17	**0.005**	0.12	**0.010**	0.12	**0.010**	0.21	**0.005**	0.07	0.13
2	0.16	**0.005**	0.16	**0.005**	0.10	**0.005**	0.21	**0.005**	0.12	**0.005**	0.13	**0.010**	0.16	**0.005**	0.08	0.12
3	0.22	**0.005**	0.19	**0.005**	0.08	**0.020**	0.16	**0.005**	0.13	**0.005**	0.18	**0.005**	0.16	**0.005**	0.09	0.055
4	0.19	**0.005**	0.19	**0.005**	0.14	**0.005**	0.15	**0.005**	0.09	0.055	0.12	**0.005**	0.20	**0.005**	0.09	**0.045**
5	0.15	**0.005**	0.15	**0.005**	0.08	**0.015**	0.17	**0.005**	0.12	**0.005**	0.15	**0.005**	0.15	**0.005**	0.12	**0.010**

p-values are corrected using the Bonferroni method over 5 comparisons; significant p-values are in bold; r: Pearson's correlation coefficient

Table H. Statistics of the latent dimensions for corrected cortical thickness.

	Main/Mixed sample						Women				Men			
	1st LD		2nd LD		3rd LD		1st LD		2nd LD		1st LD		2nd LD	
	Cardiomeatbolic health		Physical robustness				Cardiomeatbolic health		Physical robustness		Cardiomeatbolic health		Physical robustness	
Split	r	p-value	r	p-value	r	p-value	r	p-value	r	p-value	r	p-value	r	p-value
1	0.34	**0.005**	0.11	**0.005**	0.09	**0.005**	0.29	**0.005**	0.01	>0.99	0.31	**0.005**	0.17	**0.005**
2	0.33	**0.005**	0.06	**0.024**	0.01	>0.99	0.32	**0.005**	0.003	>0.99	0.34	**0.005**	0.11	**0.005**
3	0.30	**0.005**	0.11	**0.005**	0.08	**0.005**	0.30	**0.005**	0.05	0.479	0.32	**0.005**	0.20	**0.010**
4	0.32	**0.005**	0.12	**0.005**	0.11	**0.005**	0.30	**0.005**	0.12	**0.010**	0.31	**0.005**	0.18	**0.005**
5	0.30	**0.005**	0.09	**0.005**	0.08	**0.005**	0.26	**0.005**	0.03	0.374	0.32	**0.005**	0.17	**0.015**

p-values are corrected using the Bonferroni method over 5 comparisons; significant p-values are in bold; r: Pearson's correlation coefficient

Table I. Statistics of the latent dimensions for corrected grey matter volume.

	Main/Mixed sample						Women						Men			
	1st LD		2nd LD		3rd LD		1st LD		2nd LD		3rd LD		1st LD		2nd LD	
	Cardiomeatbolic health		Physical robustness				Cardiomeatbolic health		Physical robustness				Cardiomeatbolic health		Physical robustness	
Split	r	p-value	r	p-value	r	p-value	r	p-value	r	p-value	r	p-value	r	p-value	r	p-value
1	0.22	**0.005**	0.14	**0.005**	0.07	**0.020**	0.17	**0.005**	0.08	0.080	0.09	**0.030**	0.12	**0.010**	0.16	**0.005**
2	0.19	**0.005**	0.13	**0.005**	0.04	0.329	0.21	**0.005**	0.11	**0.005**	0.05	0.414	0.16	**0.005**	0.13	**0.005**
3	0.23	**0.005**	0.09	**0.005**	0.06	**0.035**	0.14	**0.005**	0.13	**0.010**	0.12	**0.010**	0.18	**0.005**	0.17	**0.005**
4	0.19	**0.005**	0.12	**0.005**	0.10	**0.005**	0.15	**0.005**	0.09	**0.030**	0.02	<0.99	0.12	**0.010**	0.17	**0.005**
5	0.17	**0.005**	0.11	**0.005**	0.06	**0.025**	0.16	**0.005**	0.10	**0.030**	0.06	0.264	0.15	**0.005**	0.13	**0.005**

p-values are corrected using the Bonferroni method over 5 comparisons; significant p-values are in bold; r: Pearson's correlation coefficient

Table J. Association between the brain loadings of the latent dimension of cardiometabolic health and neuromaps.

			CT Main/MIxed						GMV Main/MIxed					
			Raw		Proportional		Corrected		Raw		Proportional		Corrected	
id	Category	Subcategory	r	p-value	r	p-value	r	p-value	r	p-value	r	p-value	r	p-value
abagen_genepc1_fsaverage_10k	Genetics	PC1 Allen	-0.647	0.026	-0.599	0.103	-0.625	0.026	-0.024	> 0.999	-0.147	> 0.999	-0.164	> 0.999
aghourian2017_feobv_MNI152_1mm	Molecular	VAChT	0.524	> 0.999	0.525	> 0.999	0.525	> 0.999	0.012	> 0.999	-0.033	> 0.999	0.115	> 0.999
alarkurtti2015_raclopride_MNI152_3mm	Molecular	D2	-0.179	> 0.999	-0.197	> 0.999	-0.198	> 0.999	0.309	> 0.999	0.290	> 0.999	0.289	> 0.999
bedard2019_feobv_MNI152_1mm	Molecular	VAChT	0.729	0.026	0.711	0.026	0.729	0.026	0.267	> 0.999	0.148	> 0.999	0.277	> 0.999
beliveau2017_az10419369_fsaverage_164k	Molecular	5-HT1b	-0.413	> 0.999	-0.425	> 0.999	-0.424	> 0.999	-0.411	0.052	-0.481	0.026	-0.306	> 0.999
beliveau2017_cimbi36_fsaverage_164k	Molecular	5-HT2a	0.083	> 0.999	0.053	> 0.999	0.069	> 0.999	0.010	> 0.999	0.312	> 0.999	0.226	> 0.999
beliveau2017_cumi101_fsaverage_164k	Molecular	5-HT1a	0.732	0.026	0.685	0.026	0.709	0.026	0.275	> 0.999	0.454	> 0.999	0.380	> 0.999
beliveau2017_dasb_fsaverage_164k	Molecular	5-HTT	0.516	> 0.999	0.538	0.284	0.541	> 0.999	-0.052	> 0.999	-0.359	> 0.999	-0.224	> 0.999

beliveau2017_sb207145_fsaverage_164k	Molecular	5-HT4	0.420	> 0.999	0.381	> 0.999	0.405	> 0.999	0.314	> 0.999	0.598	0.542	0.477	> 0.999
castrillon2023_cmrglc_MNI152_3mm	Metabolism	Glucose	-0.281	> 0.999	-0.324	> 0.999	-0.316	> 0.999	-0.241	> 0.999	-0.062	> 0.999	-0.013	> 0.999
ding2010_mrb_MNI152_1mm	Molecular	NET	-0.205	> 0.999	-0.199	> 0.999	-0.223	> 0.999	-0.041	> 0.999	0.033	> 0.999	0.090	> 0.999
dubois2015_abp688_MNI152_1mm	Molecular	mGluR5	0.365	> 0.999	0.330	> 0.999	0.350	> 0.999	-0.094	> 0.999	0.117	> 0.999	0.170	> 0.999
dukart2018_flumazenil_MNI152_3mm	Molecular	GABAa	-0.049	> 0.999	-0.041	> 0.999	-0.048	> 0.999	-0.212	> 0.999	-0.132	> 0.999	-0.033	> 0.999
dukart2018_fpcit_MNI152_3mm	Molecular	DAT	0.575	0.026	0.553	0.026	0.571	0.026	0.238	> 0.999	0.034	> 0.999	0.043	> 0.999
fazio2016_madam_MNI152_3mm	Molecular	5-HTT	0.282	> 0.999	0.262	> 0.999	0.275	> 0.999	-0.169	> 0.999	-0.401	0.671	-0.349	> 0.999
finnema2016_ucbj_MNI152_1mm	Molecular	SV2a	-0.054	> 0.999	-0.061	> 0.999	-0.067	> 0.999	-0.209	> 0.999	0.043	> 0.999	0.011	> 0.999
gallezot2010_p943_MNI152_1mm	Molecular	5-HT1b	-0.428	> 0.999	-0.427	> 0.999	-0.427	> 0.999	-0.424	0.026	-0.343	> 0.999	-0.239	> 0.999
gallezot2017_gsk189254_MNI152_1mm	Molecular	H3	0.194	> 0.999	0.164	> 0.999	0.170	> 0.999	-0.405	0.026	-0.220	> 0.999	-0.167	> 0.999
galovic2021_ge179_MNI152_1mm	Molecular	NMDA	0.360	> 0.999	0.327	> 0.999	0.342	> 0.999	0.044	> 0.999	0.257	> 0.999	0.167	> 0.999
hcps1200_megalpha_fsLR_4k	Brain function	MEG 1alpha	-0.434	> 0.999	-0.343	> 0.999	-0.373	> 0.999	0.195	> 0.999	0.088	> 0.999	0.017	> 0.999

hcps1200_megbeta_fsLR_4k	Brain function	MEG 2beta	-0.242	> 0.999	-0.264	> 0.999	-0.273	> 0.999	0.042	> 0.999	0.073	> 0.999	0.106	> 0.999
hcps1200_megdelta_fsLR_4k	Brain function	MEG 3delta	0.498	> 0.999	0.437	> 0.999	0.476	> 0.999	-0.203	> 0.999	-0.152	> 0.999	-0.061	> 0.999
hcps1200_meggamma1_fsLR_4k	Brain function	MEG 4low gamma	0.264	> 0.999	0.198	> 0.999	0.221	> 0.999	-0.284	> 0.999	-0.206	> 0.999	-0.110	> 0.999
hcps1200_meggamma2_fsLR_4k	Brain function	MEG 5high gamma	0.462	> 0.999	0.438	> 0.999	0.467	> 0.999	-0.258	> 0.999	-0.076	> 0.999	-0.032	> 0.999
hcps1200_megtheta_fsLR_4k	Brain function	MEG 6theta	0.292	> 0.999	0.222	> 0.999	0.238	> 0.999	-0.182	> 0.999	-0.096	> 0.999	-0.014	> 0.999
hcps1200_megtimescale_fsLR_4k	Brain function	MEG intts	0.488	> 0.999	0.428	> 0.999	0.474	> 0.999	-0.226	> 0.999	-0.119	> 0.999	-0.082	> 0.999
hcps1200_myelinmap_fsLR_32k	Brain structure	T1w/T2w	-0.426	> 0.999	-0.361	> 0.999	-0.388	> 0.999	0.054	> 0.999	-0.238	> 0.999	-0.180	> 0.999
hcps1200_thickness_fsLR_32k	Brain structure	CT	0.731	0.026	0.672	0.026	0.706	0.026	0.062	> 0.999	0.325	> 0.999	0.264	> 0.999
hesse2017_methylreboxetine_MNI152_3mm	Molecular	NET	0.286	> 0.999	0.293	> 0.999	0.273	> 0.999	0.065	> 0.999	0.100	> 0.999	0.175	> 0.999
hillmer2016_flubatine_MNI152_1mm	Molecular	a4b2	-0.174	> 0.999	-0.205	> 0.999	-0.208	> 0.999	-0.112	> 0.999	0.044	> 0.999	0.090	> 0.999
jaworska2020_fallypride_MNI152_1mm	Molecular	D2	0.582	0.155	0.516	> 0.999	0.542	> 0.999	0.275	> 0.999	0.551	0.516	0.454	> 0.999
kaller2017_sch23390_MNI152_3mm	Molecular	D1	0.366	> 0.999	0.331	> 0.999	0.350	> 0.999	0.056	> 0.999	0.100	> 0.999	0.084	> 0.999

kantonen2020_carfentanil_MNI152_3mm	Molecular	MOR	0.461	> 0.999	0.394	> 0.999	0.421	> 0.999	-0.059	> 0.999	0.154	> 0.999	0.210	> 0.999
kim2020_ps13_MNI152_2mm	Molecular	COX-1	-0.312	> 0.999	-0.245	> 0.999	-0.256	> 0.999	0.156	> 0.999	-0.190	> 0.999	-0.153	> 0.999
laurikainen2018_fmpepd2_MNI152_1mm	Molecular	CB1	-0.030	> 0.999	-0.077	> 0.999	-0.069	> 0.999	-0.125	> 0.999	0.195	> 0.999	0.211	> 0.999
lois2018_pbr28_MNI152_2mm	Molecular	TSPO	0.543	0.052	0.519	0.026	0.536	0.052	0.198	> 0.999	0.032	> 0.999	0.066	> 0.999
lukow2022_ro154513_MNI152_2mm	Molecular	GABAa	0.636	0.026	0.587	0.026	0.612	0.026	-0.032	> 0.999	0.195	> 0.999	0.182	> 0.999
malen2022_raclopride_MNI152_2mm	Molecular	D2	0.244	> 0.999	0.214	> 0.999	0.230	> 0.999	0.401	0.026	0.364	0.181	0.285	0.103
margulies2016_fcgradient01_fsLR_32k	Brain function	FC Grad01	0.219	> 0.999	0.149	> 0.999	0.181	> 0.999	-0.047	> 0.999	0.271	> 0.999	0.185	> 0.999
margulies2016_fcgradient02_fsLR_32k	Brain function	FC Grad02	-0.390	> 0.999	-0.390	> 0.999	-0.382	> 0.999	0.023	> 0.999	-0.023	> 0.999	-0.192	> 0.999
margulies2016_fcgradient03_fsLR_32k	Brain function	FC Grad03	-0.163	> 0.999	-0.197	> 0.999	-0.188	> 0.999	-0.125	> 0.999	-0.116	> 0.999	-0.153	> 0.999
margulies2016_fcgradient04_fsLR_32k	Brain function	FC Grad04	0.162	> 0.999	0.219	> 0.999	0.213	> 0.999	-0.277	> 0.999	-0.548	0.026	-0.401	> 0.999
margulies2016_fcgradient05_fsLR_32k	Brain function	FC Grad05	0.571	0.026	0.542	0.052	0.566	0.026	0.192	> 0.999	0.379	> 0.999	0.225	> 0.999
margulies2016_fcgradient06_fsLR_32k	Brain function	FC Grad06	-0.315	> 0.999	-0.303	> 0.999	-0.306	> 0.999	-0.234	> 0.999	-0.198	> 0.999	-0.251	> 0.999

margulies2016_fcgradient07_fsLR_32k	Brain function	FC Grad07	0.070	> 0.999	0.051	> 0.999	0.056	> 0.999	-0.062	> 0.999	-0.376	> 0.999	-0.320	> 0.999
margulies2016_fcgradient08_fsLR_32k	Brain function	FC Grad08	0.096	> 0.999	0.080	> 0.999	0.086	> 0.999	0.379	0.026	0.252	> 0.999	0.093	> 0.999
margulies2016_fcgradient09_fsLR_32k	Brain function	FC Grad09	-0.033	> 0.999	-0.042	> 0.999	-0.041	> 0.999	-0.330	> 0.999	-0.266	> 0.999	-0.130	> 0.999
margulies2016_fcgradient10_fsLR_32k	Brain function	FC Grad10	-0.228	> 0.999	-0.204	> 0.999	-0.210	> 0.999	-0.326	0.026	-0.238	> 0.999	-0.182	> 0.999
mueller2013_intersubjvar_fsLR_164k	Brain function	RSFC intvar	0.158	> 0.999	0.088	> 0.999	0.119	> 0.999	0.144	> 0.999	0.450	> 0.999	0.347	> 0.999
naganawa2020_lsn3172176_MNI152_1mm	Molecular	M1	-0.097	> 0.999	-0.115	> 0.999	-0.118	> 0.999	-0.238	> 0.999	0.035	> 0.999	0.027	> 0.999
neurosynth_cogpc1_MNI152_2mm	Cognition	PC1 Cognition	-0.250	> 0.999	-0.209	> 0.999	-0.236	> 0.999	0.096	> 0.999	0.071	> 0.999	0.078	> 0.999
norgaard2021_flumazenil_fsaverage_164k	Molecular	GABAa	-0.113	> 0.999	-0.101	> 0.999	-0.098	> 0.999	-0.322	0.929	-0.294	> 0.999	-0.134	> 0.999
normandin2015_omar_MNI152_1mm	Molecular	CB1	0.367	> 0.999	0.326	> 0.999	0.341	> 0.999	-0.106	> 0.999	0.172	> 0.999	0.217	> 0.999
radnakrishnan2018_gsk215083_MNI152_1mm	Molecular	5-HT6	0.057	> 0.999	0.012	> 0.999	0.028	> 0.999	-0.045	> 0.999	0.221	> 0.999	0.157	> 0.999
raichle_cbf_fsLR_164k	Physiology	Blood flow	-0.235	> 0.999	-0.236	> 0.999	-0.250	> 0.999	-0.257	> 0.999	-0.514	> 0.999	-0.327	> 0.999
raichle_cbv_fsLR_164k	Physiology	Blood vol	0.176	> 0.999	0.210	> 0.999	0.202	> 0.999	-0.171	> 0.999	-0.333	> 0.999	-0.329	> 0.999

raichle_cmr02_fsLR_164k	Metabolism	Oxygen	-0.599	0.129	-0.581	0.361	-0.608	0.284	-0.045	> 0.999	-0.260	> 0.999	-0.213	> 0.999
raichle_cmrglc_fsLR_164k	Metabolism	Glucose	-0.628	0.026	-0.629	0.026	-0.643	0.026	-0.175	> 0.999	-0.279	> 0.999	-0.236	> 0.999
reardon2018_scalinghcp_civet_41k	Brain structure	Expansion	-0.167	> 0.999	-0.202	> 0.999	-0.188	> 0.999	-0.040	> 0.999	0.065	> 0.999	-0.001	> 0.999
reardon2018_scalingnih_civet_41k	Brain structure	Expansion	0.065	> 0.999	0.029	> 0.999	0.031	> 0.999	0.237	> 0.999	0.585	0.026	0.399	0.181
reardon2018_scalingpnc_civet_41k	Brain structure	Expansion	-0.076	> 0.999	-0.127	> 0.999	-0.115	> 0.999	0.111	> 0.999	0.350	> 0.999	0.195	> 0.999
rosaneto_abp688_MNI152_1mm	Molecular	mGluR5	0.230	> 0.999	0.202	> 0.999	0.216	> 0.999	-0.110	> 0.999	0.060	> 0.999	0.124	> 0.999
sandiego2015_flb457_MNI152_1mm	Molecular	D2	0.604	0.155	0.543	0.206	0.564	0.335	0.222	> 0.999	0.491	> 0.999	0.402	> 0.999
sasaki2012_fepe2i_MNI152_1mm	Molecular	DAT	0.090	> 0.999	0.082	> 0.999	0.089	> 0.999	0.297	0.026	0.158	> 0.999	0.145	> 0.999
satterthwaite2014_meancbf_MNI152_1mm	Physiology	Blood flow	0.048	> 0.999	0.018	> 0.999	0.030	> 0.999	-0.033	> 0.999	0.080	> 0.999	0.102	> 0.999
savli2012_altanserin_MNI152_3mm	Molecular	5-HT2a	0.047	> 0.999	0.009	> 0.999	0.026	> 0.999	0.064	> 0.999	0.401	> 0.999	0.349	> 0.999
savli2012_dasb_MNI152_3mm	Molecular	5-HTT	0.533	0.980	0.533	0.077	0.543	0.851	-0.112	> 0.999	-0.300	> 0.999	-0.160	> 0.999
savli2012_p943_MNI152_3mm	Molecular	5-HT1b	-0.286	> 0.999	-0.303	> 0.999	-0.299	> 0.999	-0.208	> 0.999	-0.116	> 0.999	0.015	> 0.999

savli2012_way100635_MNI152_3mm	Molecular	5-HT1a	0.630	0.026	0.583	0.026	0.599	0.026	0.260	> 0.999	0.494	> 0.999	0.416	> 0.999
smart2019_abp688_MNI152_1mm	Molecular	mGluR5	0.292	> 0.999	0.253	> 0.999	0.264	> 0.999	-0.051	> 0.999	0.163	> 0.999	0.202	> 0.999
smith2017_flb457_MNI152_1mm	Molecular	D2	0.530	0.645	0.476	> 0.999	0.501	> 0.999	0.265	> 0.999	0.505	> 0.999	0.446	> 0.999
sydnor2021_SAaxis_fsLR_32k	Brain function	Sensory-association	0.373	> 0.999	0.277	> 0.999	0.324	> 0.999	-0.021	> 0.999	0.305	> 0.999	0.233	> 0.999
tuominen_feobv_MNI152_2mm	Molecular	VAChT	0.548	0.026	0.562	0.026	0.552	0.026	0.114	> 0.999	-0.015	> 0.999	0.148	> 0.999
turtonen2020_carfentanil_MNI152_1mm	Molecular	MOR	0.429	> 0.999	0.355	> 0.999	0.385	> 0.999	-0.055	> 0.999	0.170	> 0.999	0.238	> 0.999
vijay2018_ly2795050_MNI152_2mm	Molecular	KOR	0.280	> 0.999	0.243	> 0.999	0.243	> 0.999	-0.179	> 0.999	0.087	> 0.999	0.146	> 0.999
wey2016_martinostat_MNI152_2mm	Molecular	HDAC	-0.385	0.026	-0.396	0.026	-0.402	0.026	-0.408	0.026	-0.293	0.387	-0.209	> 0.999
xu2020_FChomology_fsLR_32k	Brain function	Functional homology	-0.238	> 0.999	-0.188	> 0.999	-0.212	> 0.999	-0.099	> 0.999	-0.326	> 0.999	-0.232	> 0.999
xu2020_evoexp_fsLR_32k	Brain structure	Expansion	0.105	> 0.999	0.058	> 0.999	0.076	> 0.999	0.043	> 0.999	0.348	> 0.999	0.235	> 0.999

Table K. Association between the brain loadings of the latent dimension of physical robustness and neuromaps.

			CT Men						GMV Main/Mixed					
			Raw		Proportional		Corrected		Raw		Proportional		Corrected	
id	Category	Subcategory	r	p-value	r	p-value	r	p-value	r	p-value	r	p-value	r	p-value
abagen_genepc1_fsaverage_10k	Genetics	PC1 Allen	-0.339	> 0.999	-0.308	> 0.999	-0.478	> 0.999	-0.370	> 0.999	-0.272	> 0.999	-0.316	> 0.999
aghourian2017_feobv_MNI152_1mm	Molecular	VAChT	0.267	> 0.999	0.507	> 0.999	0.368	> 0.999	0.292	> 0.999	0.074	> 0.999	0.493	0.026
alarkurtti2015_raclopride_MNI152_3mm	Molecular	D2	0.249	> 0.999	0.074	> 0.999	-0.018	> 0.999	0.070	> 0.999	0.194	> 0.999	0.086	> 0.999
bedard2019_feobv_MNI152_1mm	Molecular	VAChT	0.181	> 0.999	0.368	> 0.999	0.422	> 0.999	0.285	> 0.999	0.124	> 0.999	0.299	> 0.999
beliveau2017_az10419369_fsaverage_164k	Molecular	5-HT1b	0.312	> 0.999	0.301	> 0.999	0.278	> 0.999	-0.050	> 0.999	-0.081	> 0.999	0.185	> 0.999
beliveau2017_cimbi36_fsaverage_164k	Molecular	5-HT2a	0.356	0.490	0.080	> 0.999	0.155	> 0.999	0.399	0.052	0.403	0.258	0.316	> 0.999
beliveau2017_cumi101_fsaverage_164k	Molecular	5-HT1a	0.257	> 0.999	0.193	> 0.999	0.375	> 0.999	0.434	0.026	0.314	> 0.999	0.297	0.851
beliveau2017_dasb_fsaverage_164k	Molecular	5-HTT	-0.138	> 0.999	0.118	> 0.999	0.268	> 0.999	-0.151	> 0.999	-0.162	> 0.999	-0.024	> 0.999

beliveau2017_sb207145_fsaverage_164k	Molecular	5-HT4	0.167	> 0.999	-0.128	> 0.999	-0.001	> 0.999	0.521	0.077	0.507	0.181	0.348	0.464
castrillon2023_cmrglc_MNI152_3mm	Metabolism	Glucose	0.276	0.697	0.119	> 0.999	0.067	> 0.999	0.242	> 0.999	0.324	0.077	0.354	0.026
ding2010_mrb_MNI152_1mm	Molecular	NET	0.068	> 0.999	0.159	> 0.999	-0.131	> 0.999	0.116	> 0.999	-0.010	> 0.999	0.236	> 0.999
dubois2015_abp688_MNI152_1mm	Molecular	mGluR5	0.360	0.284	0.244	> 0.999	0.276	> 0.999	0.547	0.026	0.476	0.026	0.536	0.026
dukart2018_flumazenil_MNI152_3mm	Molecular	GABAa	-0.027	> 0.999	-0.124	> 0.999	0.015	> 0.999	0.145	> 0.999	0.204	> 0.999	0.059	> 0.999
dukart2018_fpcit_MNI152_3mm	Molecular	DAT	0.077	> 0.999	0.153	> 0.999	0.398	> 0.999	-0.047	> 0.999	0.157	> 0.999	-0.061	> 0.999
fazio2016_madam_MNI152_3mm	Molecular	5-HTT	0.165	> 0.999	0.251	> 0.999	0.409	> 0.999	-0.042	> 0.999	-0.050	> 0.999	0.120	> 0.999
finnema2016_ucbj_MNI152_1mm	Molecular	SV2a	-0.054	> 0.999	-0.149	> 0.999	-0.174	> 0.999	0.251	0.439	0.146	> 0.999	0.250	0.052
gallezot2010_p943_MNI152_1mm	Molecular	5-HT1b	0.263	> 0.999	0.267	> 0.999	0.208	> 0.999	0.040	> 0.999	0.001	> 0.999	0.191	> 0.999
gallezot2017_gsk189254_MNI152_1mm	Molecular	H3	0.297	> 0.999	0.437	> 0.999	0.340	> 0.999	0.357	0.026	0.152	> 0.999	0.502	0.026
galovic2021_ge179_MNI152_1mm	Molecular	NMDA	0.256	> 0.999	0.193	> 0.999	0.282	> 0.999	0.403	0.026	0.297	0.697	0.432	0.026
hcps1200_megalpha_fsLR_4k	Brain function	MEG 1alpha	-0.469	> 0.999	-0.641	> 0.999	-0.614	> 0.999	-0.245	> 0.999	-0.005	> 0.999	-0.372	0.026

hcps1200_megbeta_fsLR_4k	Brain function	MEG 2beta	0.200	> 0.999	0.186	> 0.999	-0.119	> 0.999	0.136	> 0.999	0.083	> 0.999	0.261	> 0.999
hcps1200_megdelta_fsLR_4k	Brain function	MEG 3delta	0.391	> 0.999	0.549	> 0.999	0.704	0.026	0.194	> 0.999	0.040	> 0.999	0.250	> 0.999
hcps1200_meggamma1_fsLR_4k	Brain function	MEG 4low gamma	0.343	> 0.999	0.562	> 0.999	0.476	> 0.999	0.168	> 0.999	-0.056	> 0.999	0.318	0.129
hcps1200_meggamma2_fsLR_4k	Brain function	MEG 5high gamma	0.250	> 0.999	0.405	> 0.999	0.596	> 0.999	0.167	> 0.999	0.033	> 0.999	0.181	> 0.999
hcps1200_megtheta_fsLR_4k	Brain function	MEG 6theta	0.460	> 0.999	0.622	> 0.999	0.493	> 0.999	0.239	> 0.999	0.105	> 0.999	0.401	0.026
hcps1200_megtimescale_fsLR_4k	Brain function	MEG intts	0.315	> 0.999	0.448	> 0.999	0.639	0.258	0.189	> 0.999	0.015	> 0.999	0.189	> 0.999
hcps1200_myelinmap_fsLR_32k	Brain structure	T1w/T2w	-0.379	> 0.999	-0.280	> 0.999	-0.369	> 0.999	-0.485	0.026	-0.296	> 0.999	-0.450	0.026
hcps1200_thickness_fsLR_32k	Brain structure	CT	0.232	> 0.999	0.258	> 0.999	0.381	> 0.999	0.480	0.026	0.253	> 0.999	0.363	> 0.999
hesse2017_methylreboxetine_MNI152_3mm	Molecular	NET	-0.084	> 0.999	0.078	> 0.999	-0.024	> 0.999	0.188	> 0.999	-0.006	> 0.999	0.209	> 0.999
hillmer2016_flubatine_MNI152_1mm	Molecular	a4b2	0.480	0.748	0.401	> 0.999	0.249	> 0.999	0.279	> 0.999	0.154	> 0.999	0.411	0.026
jaworska2020_fallypride_MNI152_1mm	Molecular	D2	0.061	> 0.999	-0.070	> 0.999	0.082	> 0.999	0.424	0.542	0.334	> 0.999	0.300	> 0.999
kaller2017_sch23390_MNI152_3mm	Molecular	D1	0.231	> 0.999	0.230	> 0.999	0.407	> 0.999	0.247	> 0.999	0.292	> 0.999	0.248	> 0.999

kantonen2020_carfentanil_MNI152_3mm	Molecular	MOR	0.502	0.413	0.440	> 0.999	0.474	> 0.999	0.547	0.026	0.350	> 0.999	0.582	0.026
kim2020_ps13_MNI152_2mm	Molecular	COX-1	-0.144	> 0.999	-0.191	> 0.999	-0.126	> 0.999	-0.384	0.258	-0.156	> 0.999	-0.340	0.052
laurikainen2018_fmpepd2_MNI152_1mm	Molecular	CB1	0.372	0.980	0.175	> 0.999	0.043	> 0.999	0.522	0.026	0.471	0.052	0.549	0.026
lois2018_pbr28_MNI152_2mm	Molecular	TSPO	0.001	> 0.999	0.069	> 0.999	0.273	> 0.999	0.018	> 0.999	0.144	> 0.999	0.041	> 0.999
lukow2022_ro154513_MNI152_2mm	Molecular	GABAa	0.370	> 0.999	0.366	> 0.999	0.515	> 0.999	0.459	0.026	0.252	> 0.999	0.440	0.026
malen2022_raclopride_MNI152_2mm	Molecular	D2	0.201	> 0.999	0.080	> 0.999	0.168	> 0.999	0.102	> 0.999	0.330	0.026	0.100	> 0.999
margulies2016_fcgradient01_fsLR_32k	Brain function	FC Grad01	0.276	> 0.999	0.029	> 0.999	0.147	> 0.999	0.399	0.026	0.357	> 0.999	0.309	> 0.999
margulies2016_fcgradient02_fsLR_32k	Brain function	FC Grad02	-0.208	> 0.999	-0.502	> 0.999	-0.266	> 0.999	-0.286	> 0.999	0.009	> 0.999	-0.444	0.026
margulies2016_fcgradient03_fsLR_32k	Brain function	FC Grad03	0.296	0.568	0.228	> 0.999	0.191	> 0.999	0.060	> 0.999	0.189	> 0.999	0.076	> 0.999
margulies2016_fcgradient04_fsLR_32k	Brain function	FC Grad04	-0.078	> 0.999	0.098	> 0.999	0.219	> 0.999	-0.176	> 0.999	-0.167	> 0.999	-0.022	> 0.999
margulies2016_fcgradient05_fsLR_32k	Brain function	FC Grad05	0.280	> 0.999	0.149	> 0.999	0.258	> 0.999	0.362	> 0.999	0.345	> 0.999	0.348	0.103
margulies2016_fcgradient06_fsLR_32k	Brain function	FC Grad06	-0.082	> 0.999	-0.146	> 0.999	-0.220	> 0.999	-0.010	> 0.999	0.176	> 0.999	0.013	> 0.999

margulies2016_fcgradient07_fsLR_32k	Brain function	FC Grad07	0.071	> 0.999	0.136	> 0.999	0.165	> 0.999	-0.130	> 0.999	0.014	> 0.999	-0.113	> 0.999
margulies2016_fcgradient08_fsLR_32k	Brain function	FC Grad08	-0.296	> 0.999	-0.403	0.490	-0.329	> 0.999	-0.143	> 0.999	0.033	> 0.999	-0.425	0.026
margulies2016_fcgradient09_fsLR_32k	Brain function	FC Grad09	0.361	0.103	0.456	0.026	0.401	0.929	0.143	> 0.999	-0.010	> 0.999	0.379	0.026
margulies2016_fcgradient10_fsLR_32k	Brain function	FC Grad10	0.032	> 0.999	0.131	> 0.999	0.101	> 0.999	-0.006	> 0.999	-0.032	> 0.999	0.059	> 0.999
mueller2013_intersubjvar_fsLR_164k	Brain function	RSFC intvar	0.536	0.026	0.284	> 0.999	0.262	> 0.999	0.496	0.026	0.553	0.026	0.433	0.026
naganawa2020_lsn3172176_MNI152_1mm	Molecular	M1	-0.065	> 0.999	-0.165	> 0.999	-0.203	> 0.999	0.228	> 0.999	0.090	> 0.999	0.234	0.464
neurosynth_cogpc1_MNI152_2mm	Cognition	PC1 Cognition	0.010	> 0.999	0.096	> 0.999	-0.144	> 0.999	-0.044	> 0.999	-0.144	> 0.999	0.031	> 0.999
norgaard2021_flumazenil_fsaverage_164k	Molecular	GABAa	0.099	> 0.999	0.024	> 0.999	0.125	> 0.999	0.103	> 0.999	0.114	> 0.999	0.157	> 0.999
normandin2015_omar_MNI152_1mm	Molecular	CB1	0.207	> 0.999	0.177	> 0.999	0.197	> 0.999	0.473	0.026	0.274	> 0.999	0.447	0.026
radnakrishnan2018_gsk215083_MNI152_1mm	Molecular	5-HT6	0.136	> 0.999	-0.031	> 0.999	0.009	> 0.999	0.331	0.568	0.325	0.361	0.289	0.645
raichle_cbf_fsLR_164k	Physiology	Blood flow	0.175	> 0.999	0.361	> 0.999	0.217	> 0.999	-0.167	> 0.999	-0.010	> 0.999	0.136	> 0.999
raichle_cbv_fsLR_164k	Physiology	Blood vol	-0.249	> 0.999	-0.079	> 0.999	0.010	> 0.999	-0.167	> 0.999	-0.061	> 0.999	-0.112	> 0.999

raichle_cmr02_fsLR_164k	Metabolism	Oxygen	-0.034	> 0.999	-0.030	> 0.999	-0.189	> 0.999	-0.331	> 0.999	-0.091	> 0.999	-0.115	> 0.999
raichle_cmrglc_fsLR_164k	Metabolism	Glucose	0.104	> 0.999	0.084	> 0.999	-0.089	> 0.999	-0.214	> 0.999	-0.138	> 0.999	0.000	> 0.999
reardon2018_scalinghcp_civet_41k	Brain structure	Expansion	0.304	> 0.999	0.070	> 0.999	0.053	> 0.999	0.211	> 0.999	0.606	0.026	0.015	> 0.999
reardon2018_scalingnih_civet_41k	Brain structure	Expansion	0.313	0.052	-0.027	> 0.999	-0.056	> 0.999	0.464	0.026	0.500	0.026	0.238	> 0.999
reardon2018_scalingpnc_civet_41k	Brain structure	Expansion	0.376	0.026	0.114	> 0.999	0.042	> 0.999	0.280	> 0.999	0.470	0.026	0.128	> 0.999
rosaneto_abp688_MNI152_1mm	Molecular	mGluR5	0.358	0.026	0.210	> 0.999	0.222	> 0.999	0.519	0.026	0.480	0.026	0.576	0.026
sandiego2015_flb457_MNI152_1mm	Molecular	D2	0.088	> 0.999	-0.027	> 0.999	0.135	> 0.999	0.410	0.568	0.277	> 0.999	0.252	> 0.999
sasaki2012_fepe2i_MNI152_1mm	Molecular	DAT	0.198	> 0.999	0.203	> 0.999	0.186	> 0.999	-0.032	> 0.999	0.033	> 0.999	0.068	> 0.999
satterthwaite2014_meancbf_MNI152_1mm	Physiology	Blood flow	0.337	> 0.999	0.293	> 0.999	0.271	> 0.999	0.286	> 0.999	0.235	> 0.999	0.379	0.464
savli2012_altanserin_MNI152_3mm	Molecular	5-HT2a	0.149	> 0.999	-0.044	> 0.999	0.057	> 0.999	0.351	> 0.999	0.476	0.026	0.207	> 0.999
savli2012_dasb_MNI152_3mm	Molecular	5-HTT	-0.040	> 0.999	0.177	> 0.999	0.373	> 0.999	-0.021	> 0.999	0.107	> 0.999	0.028	> 0.999
savli2012_p943_MNI152_3mm	Molecular	5-HT1b	0.239	> 0.999	0.175	> 0.999	0.101	> 0.999	0.128	> 0.999	0.262	> 0.999	0.231	> 0.999

savli2012_way100635_MNI152_3mm	Molecular	5-HT1a	0.193	> 0.999	0.128	> 0.999	0.289	> 0.999	0.398	0.284	0.354	0.748	0.240	> 0.999
smart2019_abp688_MNI152_1mm	Molecular	mGluR5	0.346	0.026	0.223	> 0.999	0.201	> 0.999	0.566	0.026	0.536	0.026	0.564	0.026
smith2017_flb457_MNI152_1mm	Molecular	D2	0.075	> 0.999	-0.054	> 0.999	0.092	> 0.999	0.424	0.181	0.295	> 0.999	0.275	> 0.999
sydnor2021_SAaxis_fsLR_32k	Brain function	Sensory-association	0.439	> 0.999	0.297	> 0.999	0.374	> 0.999	0.466	0.026	0.396	0.671	0.407	0.026
tuominen_feobv_MNI152_2mm	Molecular	VAChT	0.289	> 0.999	0.523	> 0.999	0.498	> 0.999	0.269	> 0.999	0.050	> 0.999	0.462	0.026
turtonen2020_carfentanil_MNI152_1mm	Molecular	MOR	0.548	0.052	0.490	> 0.999	0.504	> 0.999	0.544	0.026	0.390	> 0.999	0.597	0.026
vijay2018_ly2795050_MNI152_2mm	Molecular	KOR	0.319	> 0.999	0.455	> 0.999	0.339	> 0.999	0.397	0.026	0.086	> 0.999	0.508	0.026
wey2016_martinostat_MNI152_2mm	Molecular	HDAC	0.155	> 0.999	0.142	> 0.999	0.058	> 0.999	0.075	> 0.999	0.070	> 0.999	0.191	> 0.999
xu2020_FChomology_fsLR_32k	Brain function	Functional homology	-0.305	0.439	-0.053	> 0.999	-0.089	> 0.999	-0.496	0.026	-0.503	0.129	-0.414	0.026
xu2020_evoexp_fsLR_32k	Brain structure	Expansion	0.215	> 0.999	-0.023	> 0.999	-0.013	> 0.999	0.414	0.026	0.314	> 0.999	0.316	> 0.999

Table L. Statistics of the latent dimensions for subcortical and cerebellar volumes.

	Main/Mixed sample									
	1st LD		2nd LD		3rd LD		4th LD		5th LD	
Split	r	p-value	r	p-value	r	p-value	r	p-value	r	p-value
1	0.27	**0.005**	0.13	**0.005**	0.08	**0.005**	0.03	0.459	0.03	0.739
2	0.25	**0.005**	0.14	**0.005**	0.10	**0.005**	0.08	**0.005**	0.02	0.889
3	0.27	**0.005**	0.18	**0.005**	0.11	**0.005**	0.08	**0.005**	0.03	0.424
4	0.27	**0.005**	0.17	**0.005**	0.08	**0.025**	0.06	0.115	0.003	>0.99
5	0.26	**0.005**	0.16	**0.005**	0.11	**0.005**	0.05	0.105	0.06	**0.03**

p-values are corrected using the Bonferroni method over 5 comparisons; significant p-values are in bold; r: Pearson’s correlation coefficient

4. Supplementary figures

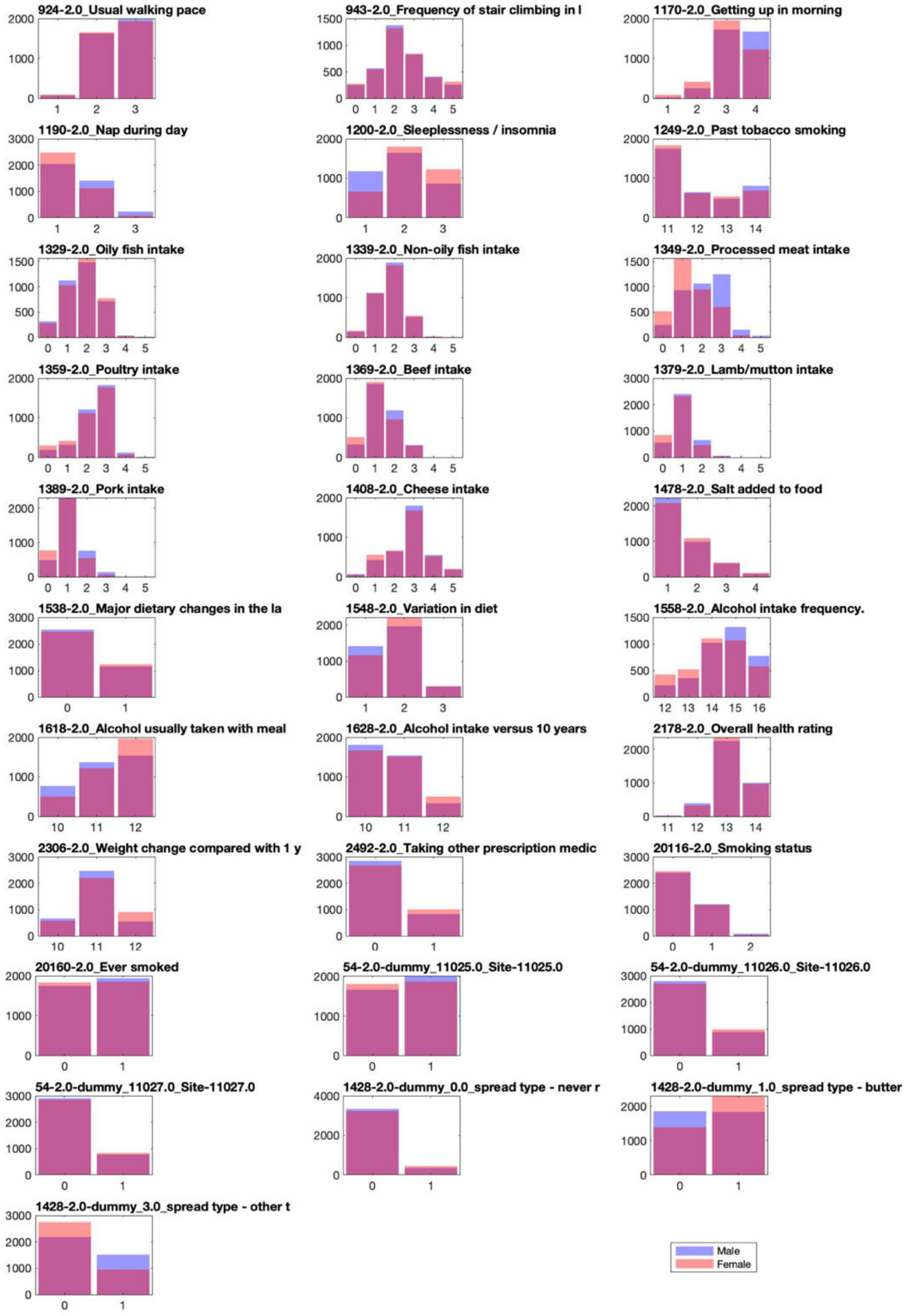

Figure A. Distribution of risk factors. Part 1. Bar plots depicting the distribution of risk factors for women and men in the analyzed samples.

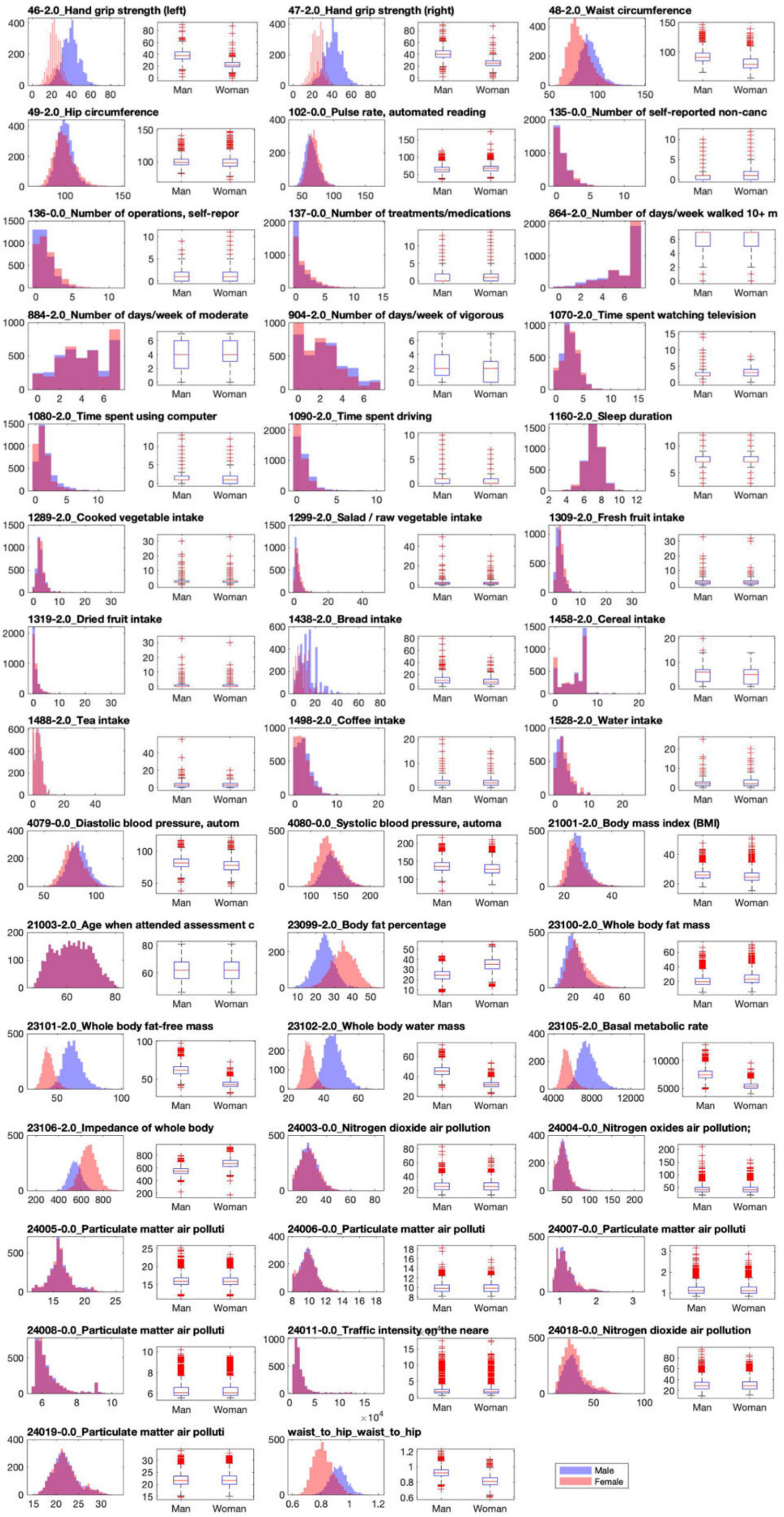

Figure B. Distribution of risk factors. Part 2. Histograms and boxplots depicting the distribution of risk factors for women and men in the analyzed samples. Data on each boxplot corresponds to the histogram to the left.

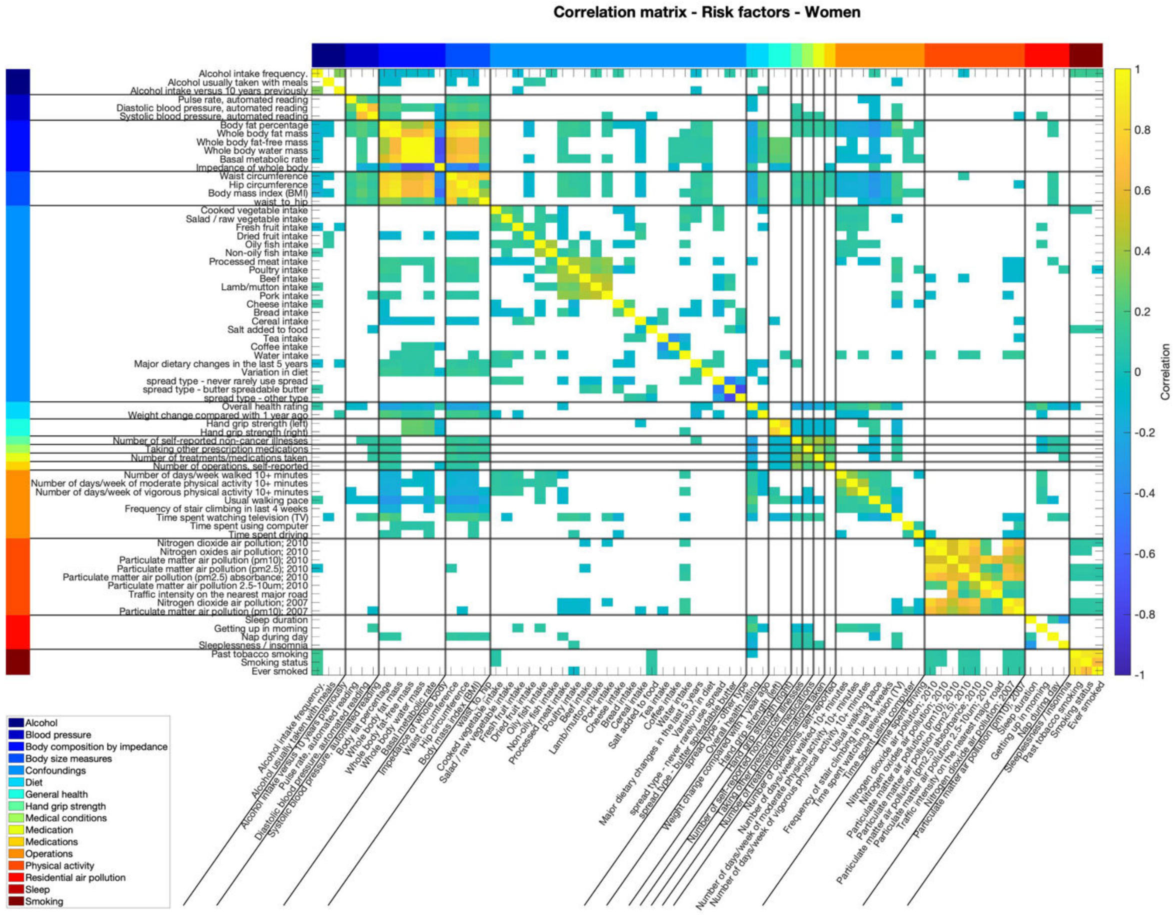

Figure C. Correlation matrix of risk factors in women. Intercorrelation among risk factors in the subsample of women. Color bar depicts the Pearson's correlation coefficient. Only significant associations after correction for multiple comparisons (Bonferroni) are shown. The colored bands indicate the categories to which the risk factors belong. The data needed to generate this figure can be found in S7_Data.

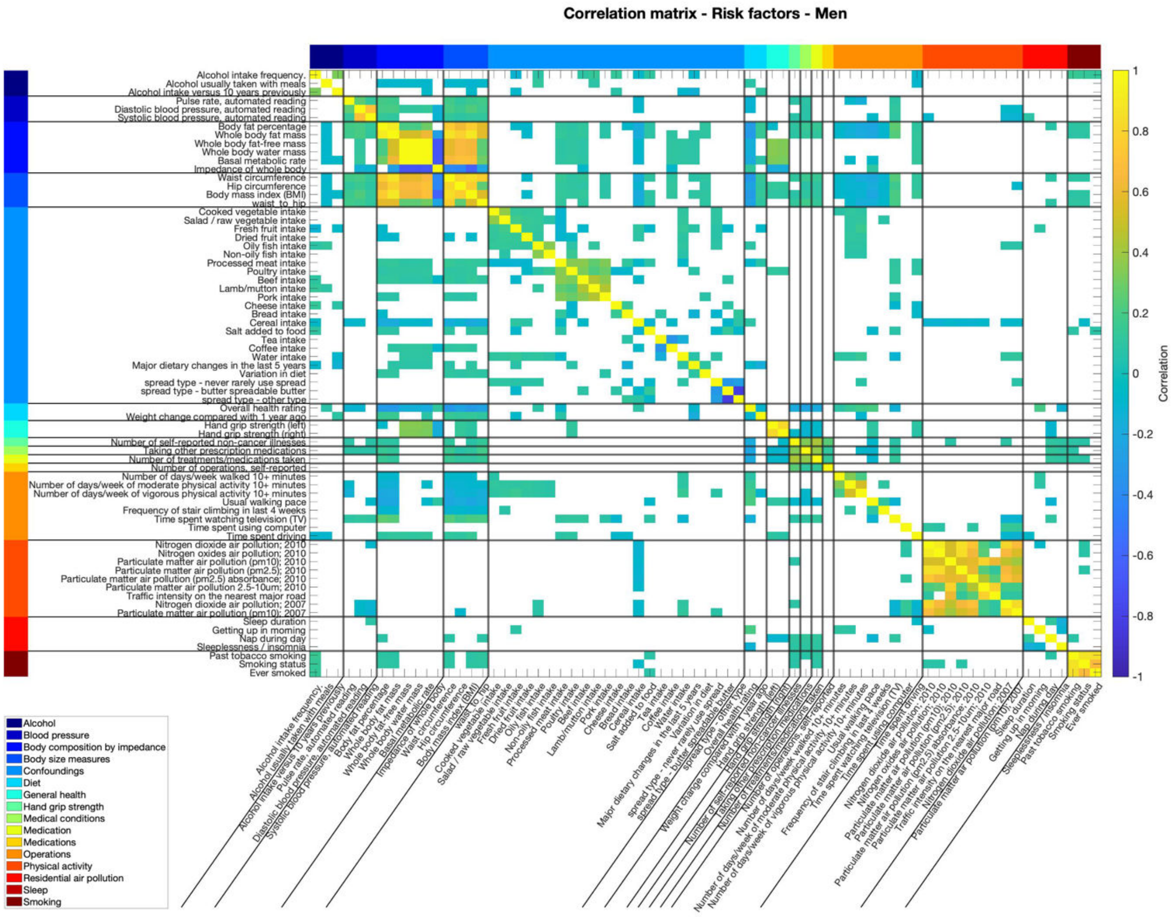

Figure D. Correlation matrix of risk factors in men. Intercorrelation among risk factors in the subsample of men. Color bar depicts the Pearson's correlation coefficient. Only significant associations after correction for multiple comparisons (Bonferroni) are shown. The colored bands indicate the categories to which the risk factors belong. The data needed to generate this figure can be found in S8_Data.

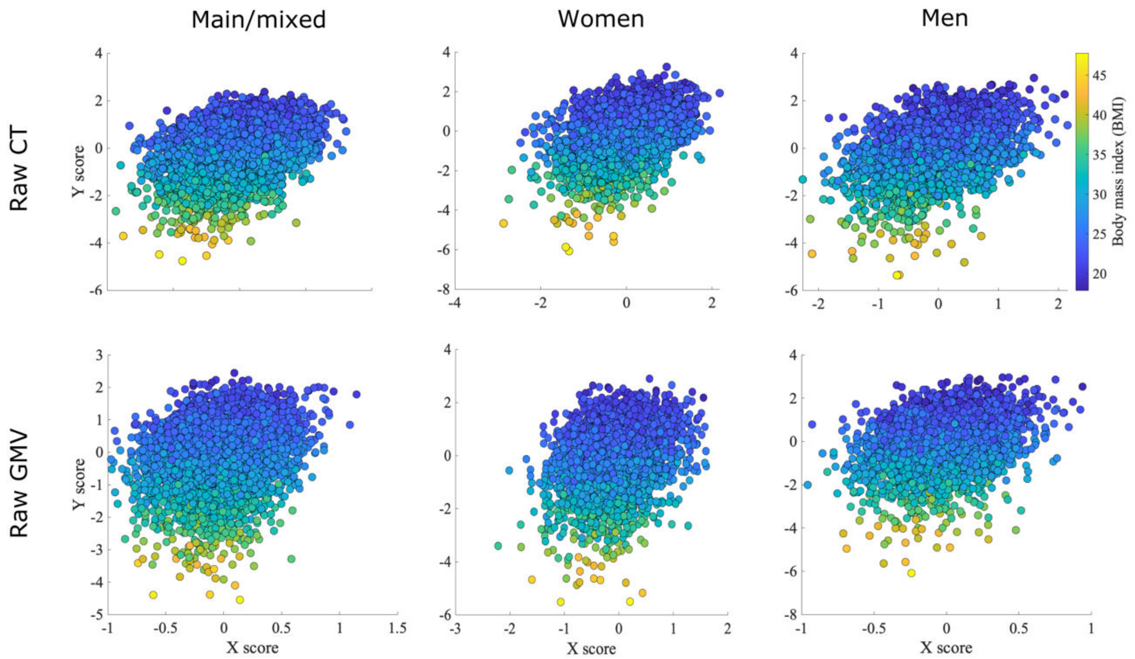

Figure E. Latent dimension of cardiometabolic health. Each scatterplot shows the brain structural (X) and risk factors (Y) scores averaged over the splits for each model. Each dot represents one participant. CT: cortical thickness. GMV: grey matter volume

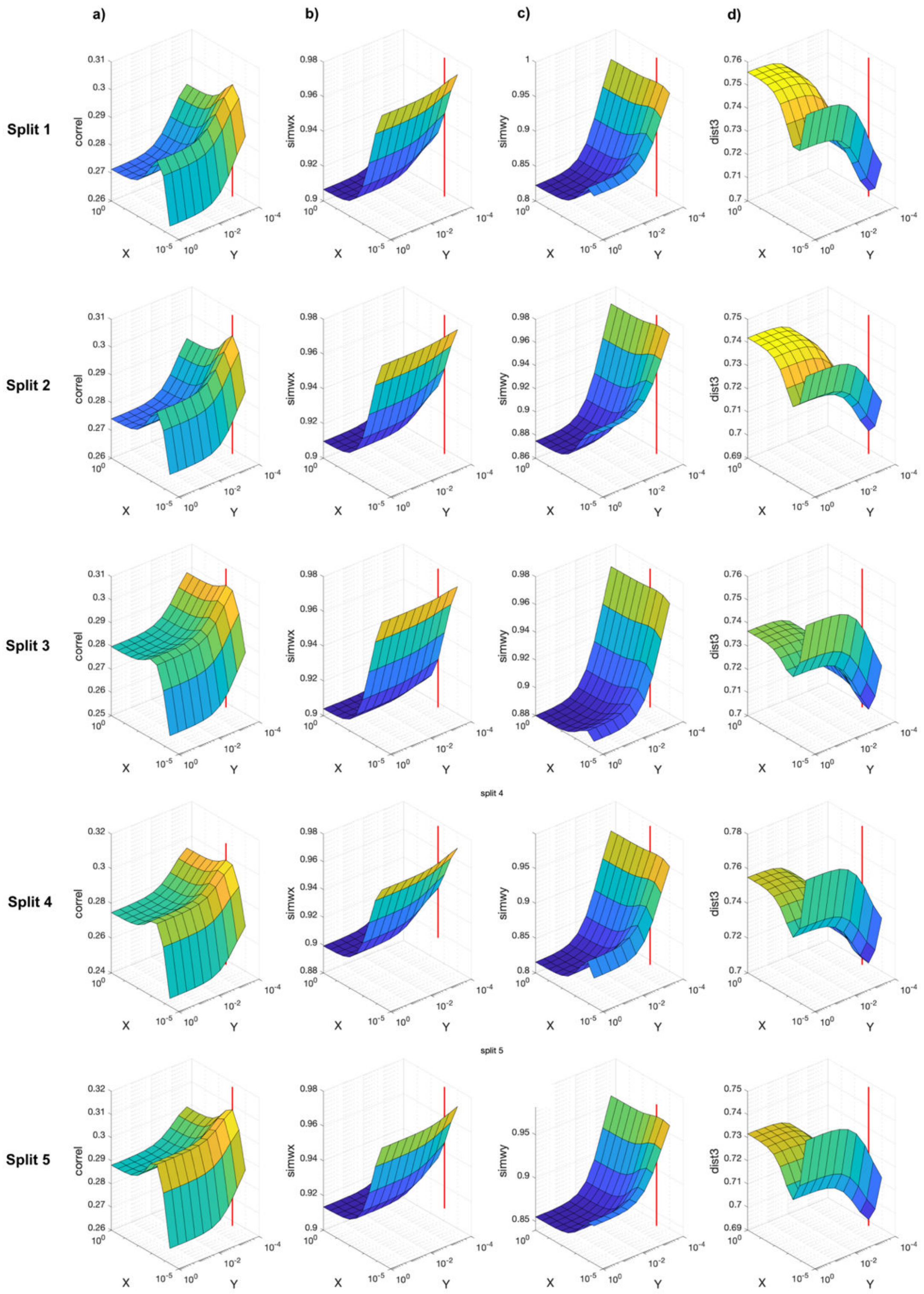

Figure F. Model optimization for the latent dimension of cardiometabolic health for raw cortical thickness in the main sample. The red line indicates the selected model. The z axis represents the test canonical correlation (column a), the similarity of weights in cortical thickness (column b) and risk factors (column c), and the joint generalizability-stability criteria (column d). The x and y axes represent 1 minus the hyperparameters tested for cortical thickness and risk factors, respectively (1-hyperparameter). The x and y axes are shown in logarithmic scale. The data needed to generate this figure can be found in S9_Data (split 1) S10_Data (split 2), S11_Data (split 3), S12_Data (split 4) and S13_Data (split 5).

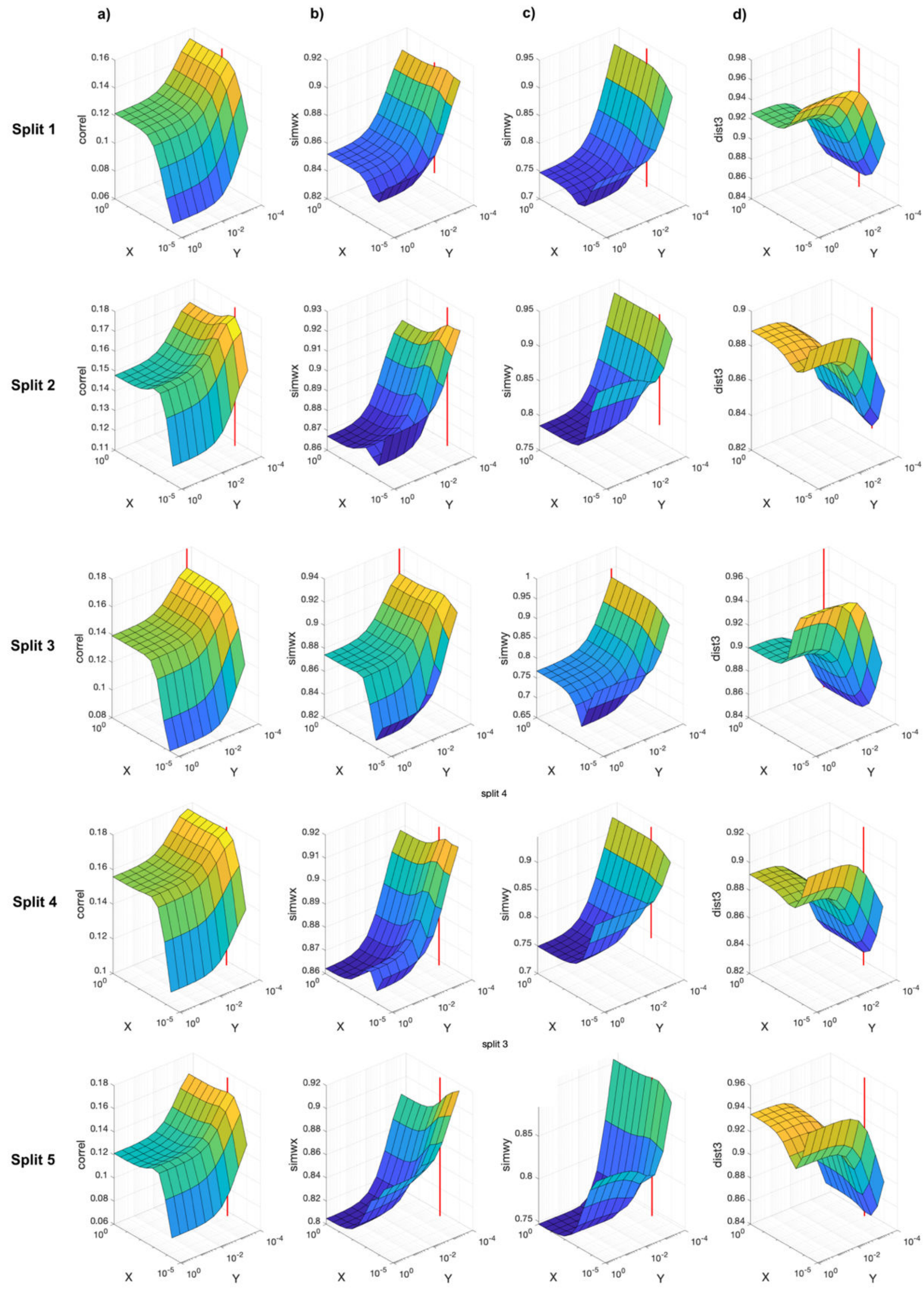

Figure G. Model optimization for the latent dimension of cardiometabolic health for raw grey matter volume in the main sample. The red line indicates the selected model. The z axis represents the test canonical correlation (column a), the similarity of weights in grey matter volume (column b) and risk factors (column c), and the joint generalizability-stability criteria (column d). The x and y axes represent 1 minus the hyperparameters tested for grey matter volume and risk factors, respectively (1-hyperparameter). The x and y axes are shown in logarithmic scale. The data needed to generate this figure can be found in S14_Data (split 1) S15_Data (split 2), S16_Data (split 3), S17_Data (split 4) and S18_Data (split 5).

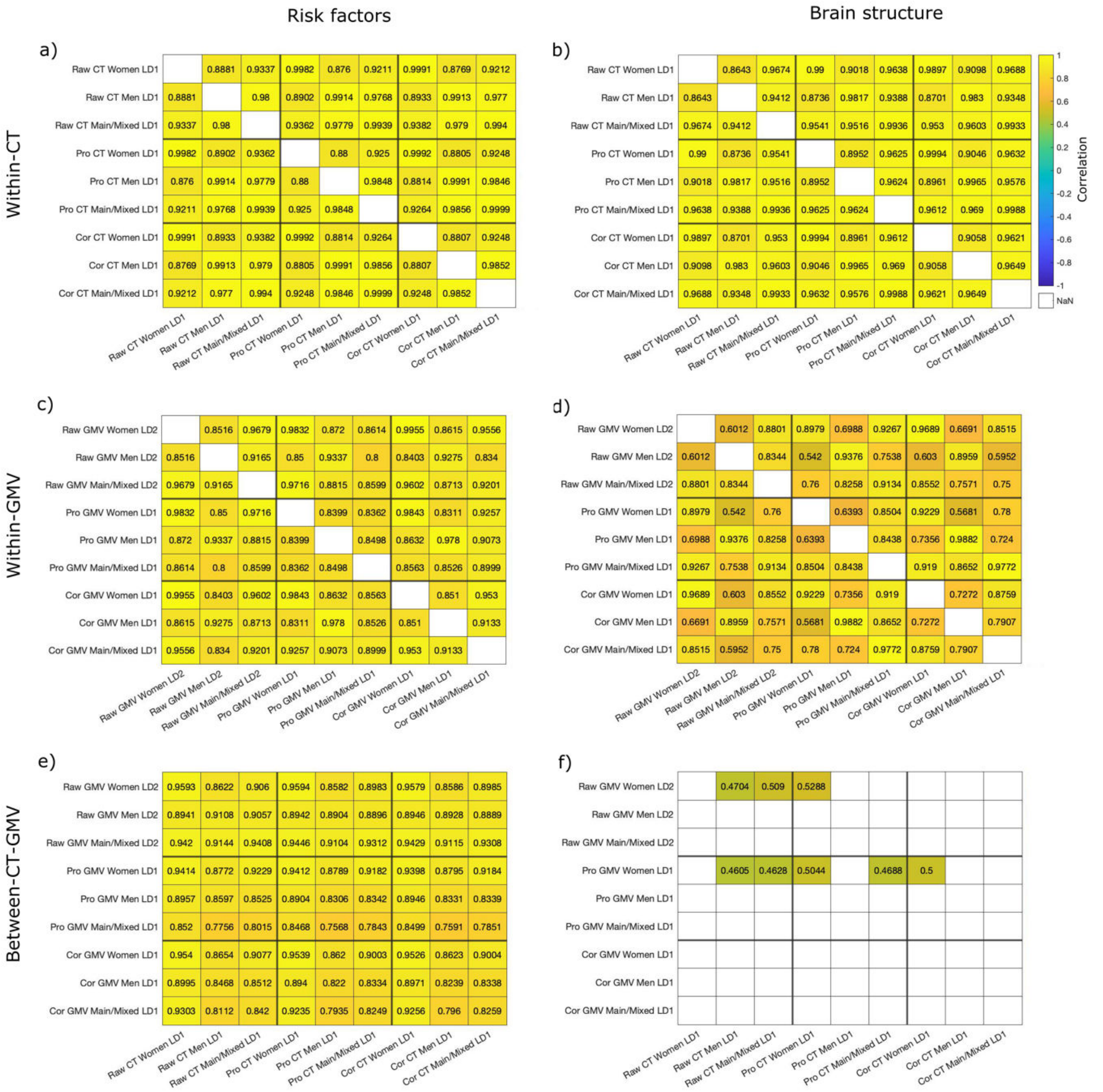

Figure H. Comparison of risk factor loadings and brain loadings for the cardiometabolic health latent dimension across samples and brain structural measures. The first row shows the Spearman correlation of a) risk factors loadings and b) brain loadings for the latent dimension of cardiometabolic health yielded with cortical thickness. The second row shows the Spearman correlation of c) risk factors loadings and d) brain loadings for the latent dimension of cardiometabolic health yielded with grey matter volume. Only comparisons that were significant after Bonferroni correction are shown. The data needed to generate this figure can be found in S19-S24_Data.

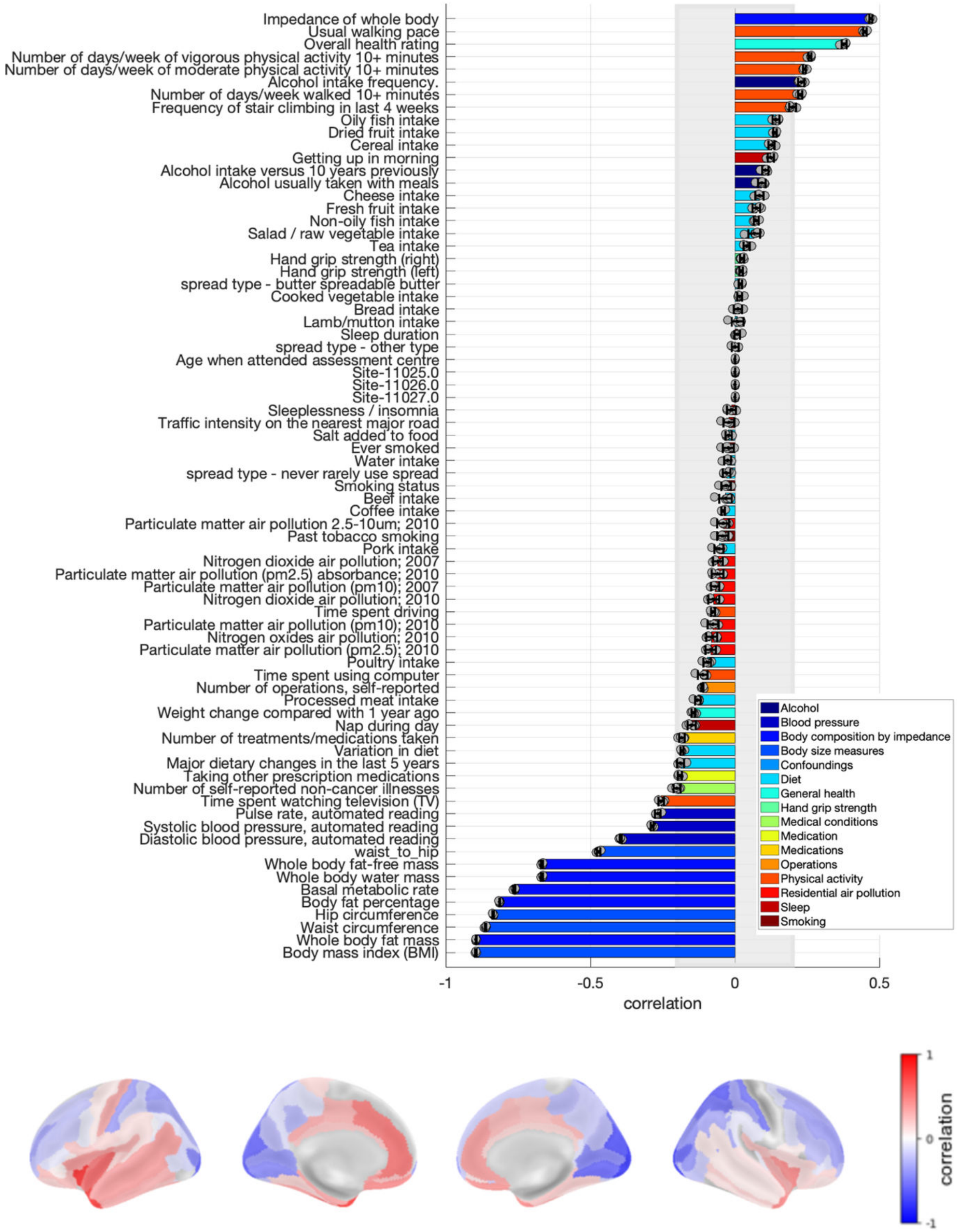

Figure I. Loadings of the latent dimension of cardiometabolic health for raw cortical thickness in women. Risk factors loadings and brain loadings. Shown loadings represent the average over the five outer splits. Error bars depict one standard deviation. The shadowed zone marks loadings between −0.2 and 0.2. The data needed to generate this figure can be found in S25_Data.

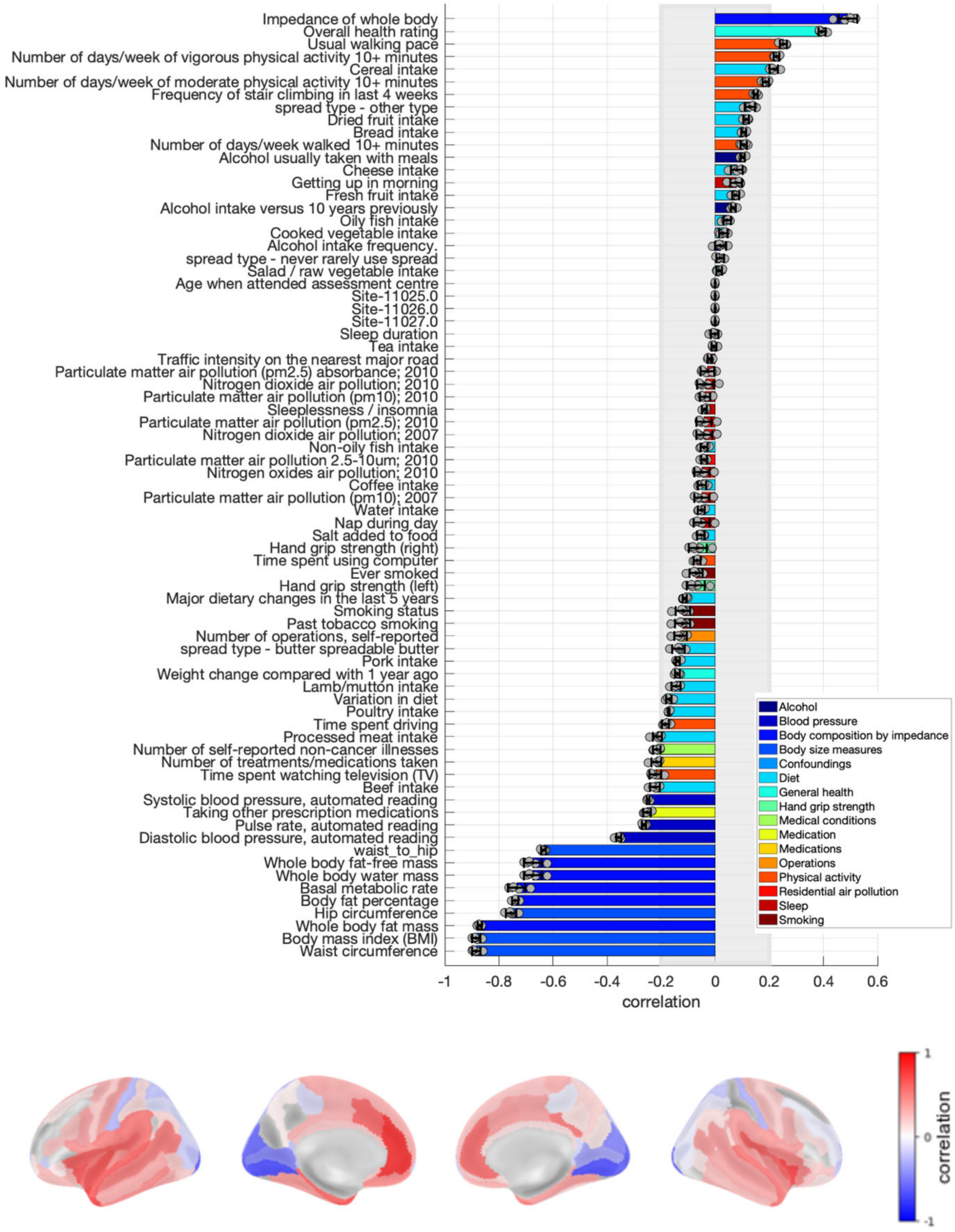

Figure J. Loadings of the latent dimension of cardiometabolic health for raw cortical thickness in men. Risk factors loadings and brain loadings. Shown loadings represent the average over the five outer splits. Error bars depict one standard deviation. The shadowed zone marks loadings between −0.2 and 0.2. The data needed to generate this figure can be found in S26_Data.

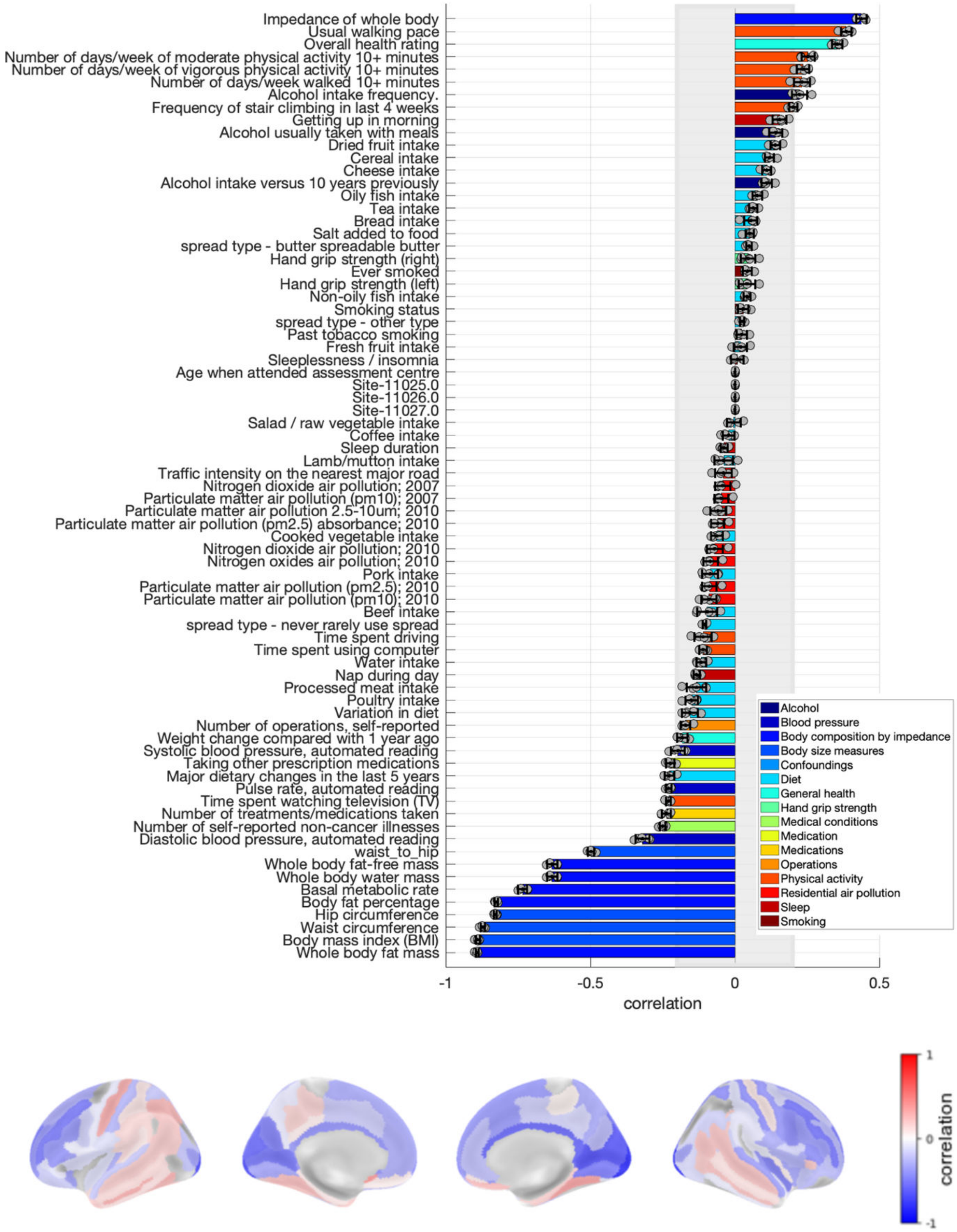

Figure K. Loadings of the latent dimension of cardiometabolic health for raw grey matter volume in women. Risk factors loadings and brain loadings. Shown loadings represent the average over the five outer splits. Error bars depict one standard deviation. The shadowed zone marks loadings between −0.2 and 0.2. The data needed to generate this figure can be found in S27_Data.

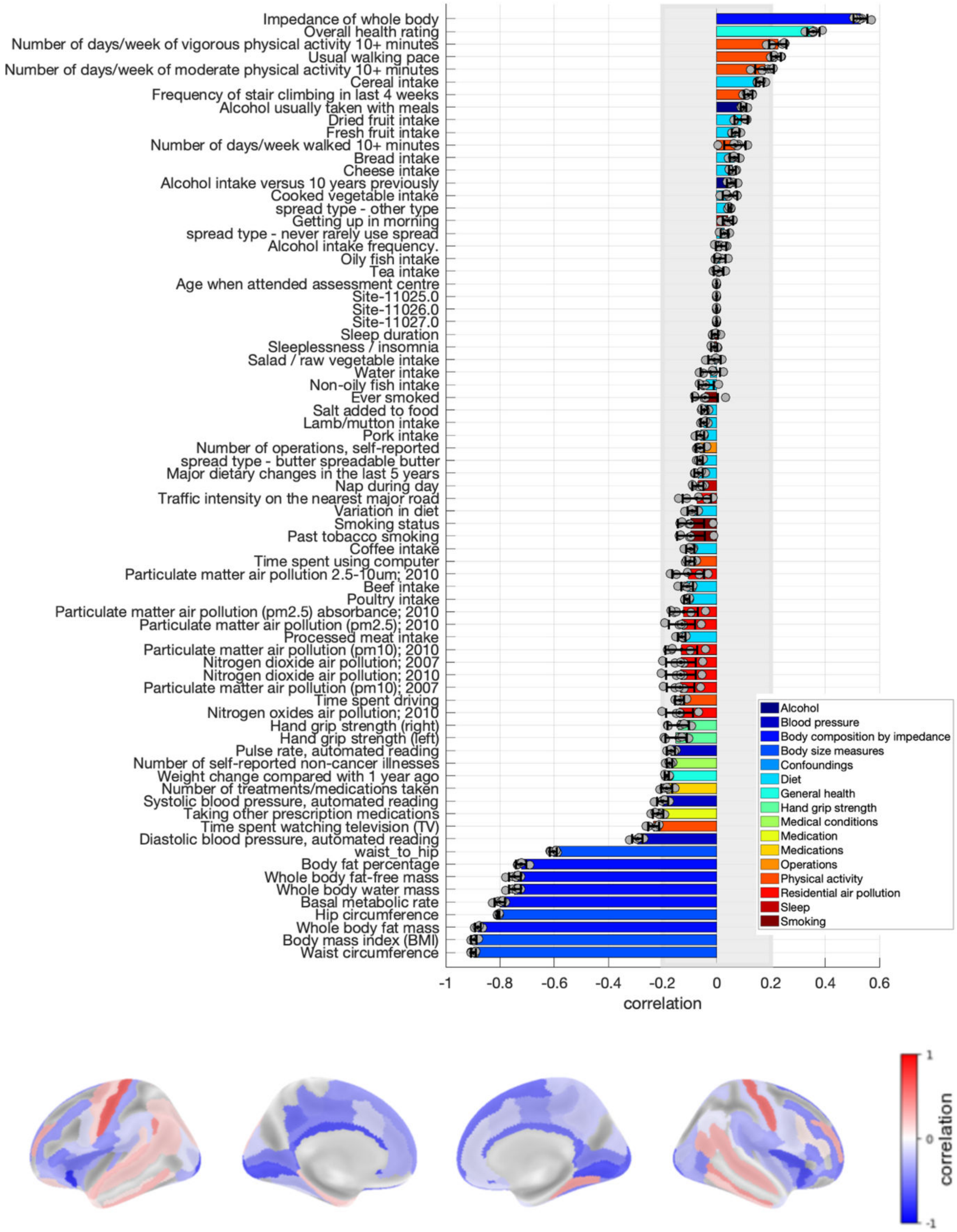

Figure L. Loadings of the latent dimension of cardiometabolic health for raw grey matter volume in men. Risk factors loadings and brain loadings. Shown loadings represent the average over the five outer splits. Error bars depict one standard deviation. The shadowed zone marks loadings between −0.2 and 0.2. The data needed to generate this figure can be found in S28_Data.

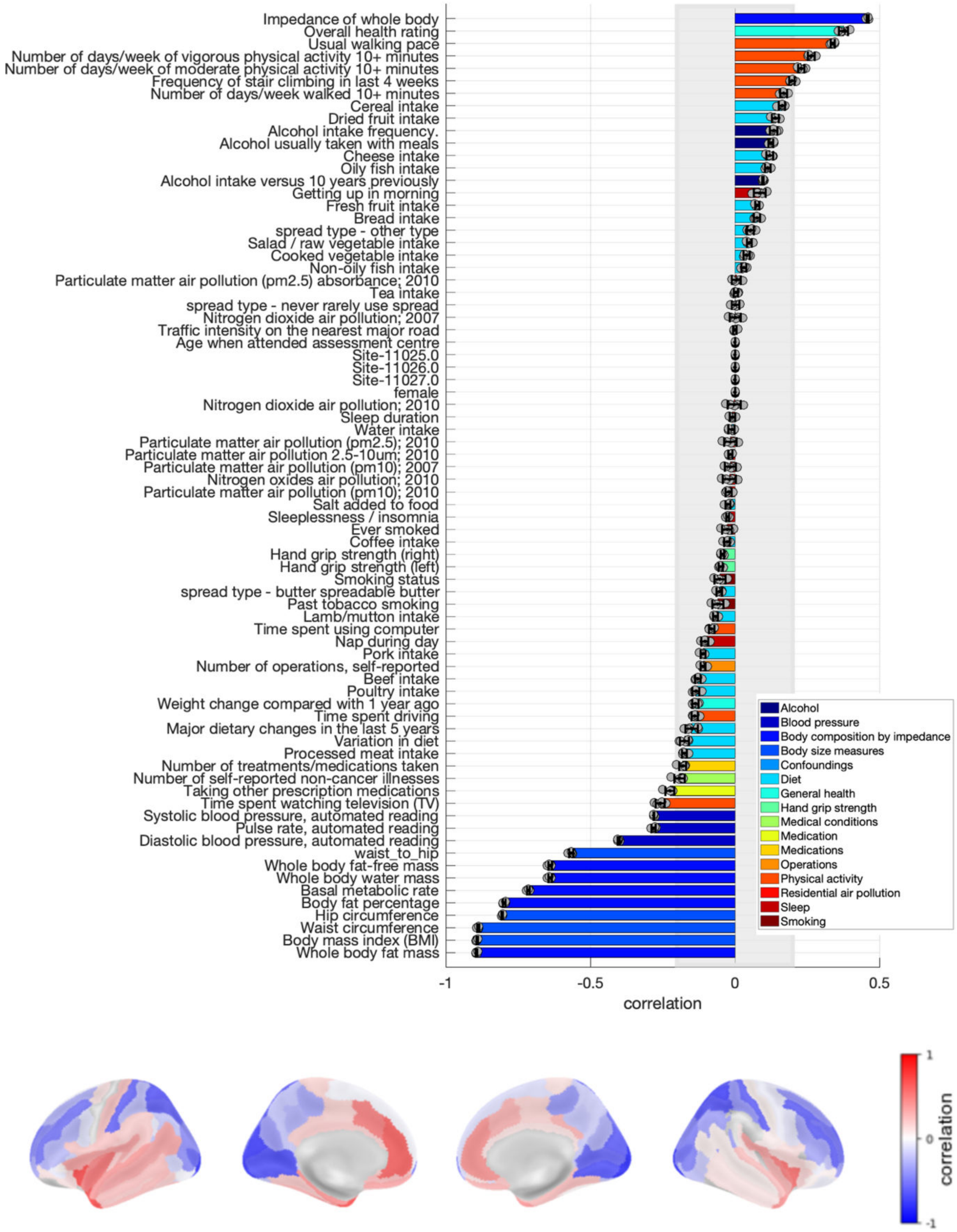

Figure M. Loadings of the latent dimension of cardiometabolic health for proportional cortical thickness in the main sample. Risk factors loadings and brain loadings. Shown loadings represent the average over the five outer splits. Error bars depict one standard deviation. The shadowed zone marks loadings between −0.2 and 0.2. The data needed to generate this figure can be found in S29_Data.

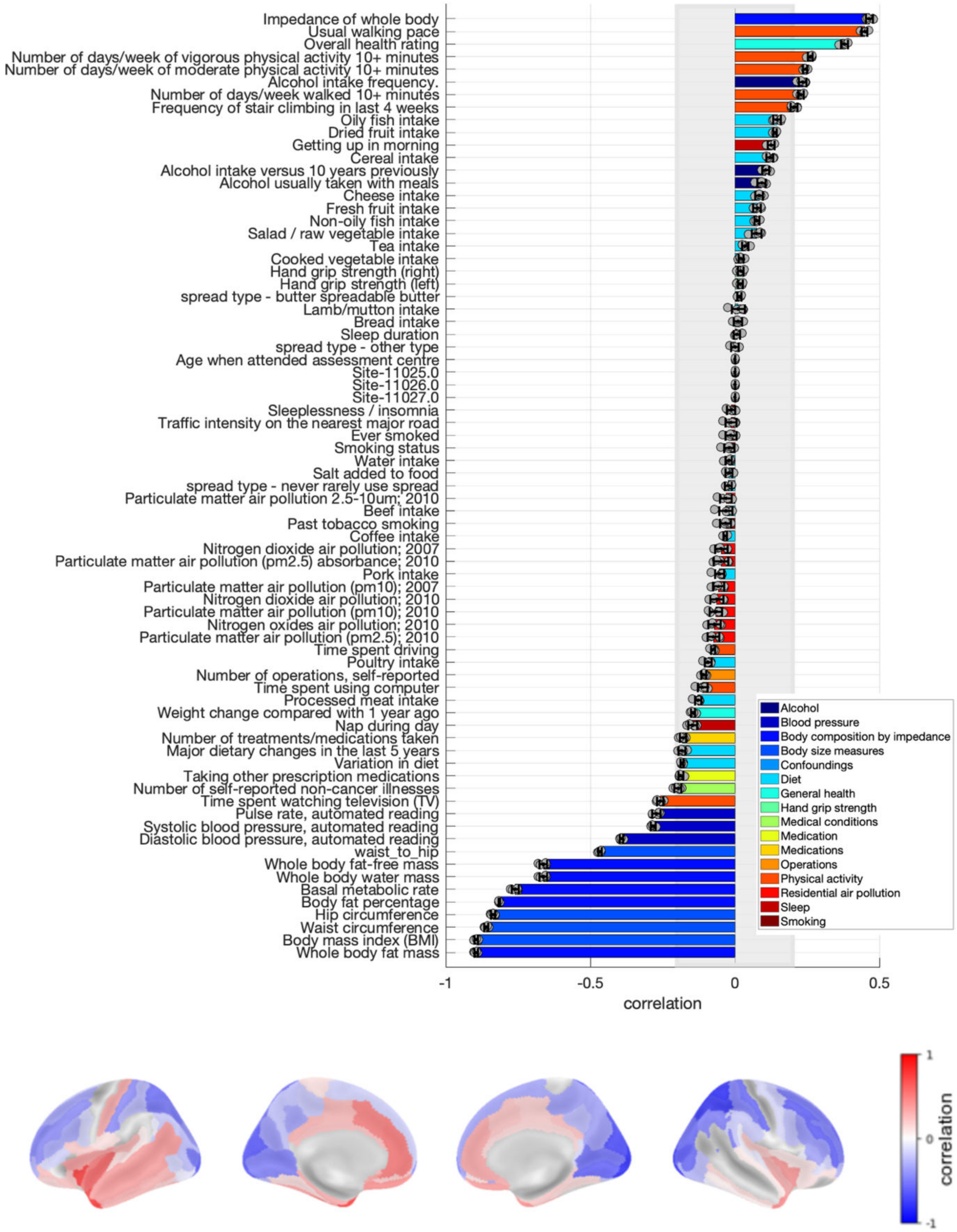

Figure N. Loadings of the latent dimension of cardiometabolic health for proportional cortical thickness in women. Risk factors loadings and brain loadings. Shown loadings represent the average over the five outer splits. Error bars depict one standard deviation. The shadowed zone marks loadings between −0.2 and 0.2. The data needed to generate this figure can be found in S30_Data.

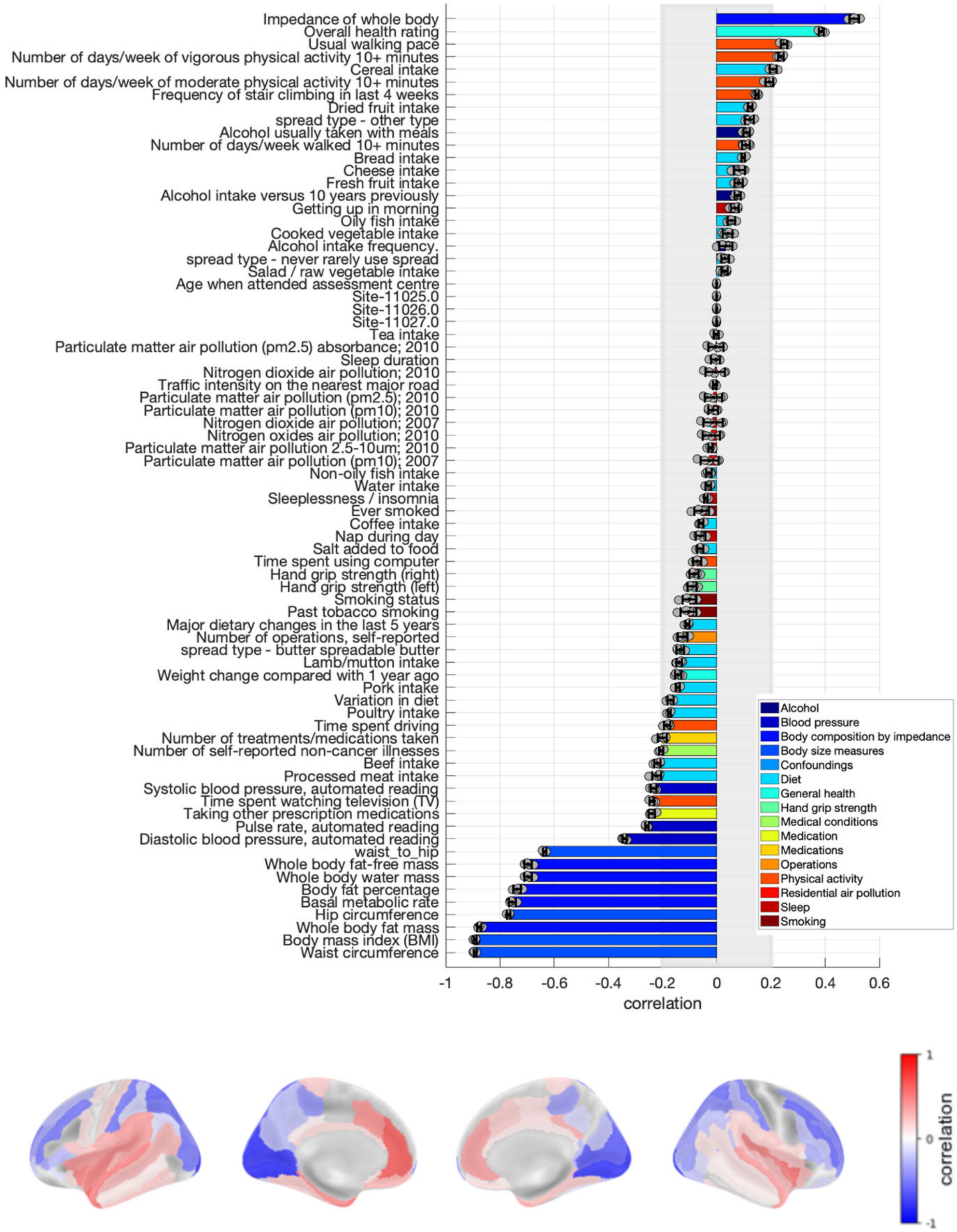

Figure O. Loadings of the latent dimension of cardiometabolic health for proportional cortical thickness in men. Risk factors loadings and brain loadings. Shown loadings represent the average over the five outer splits. Error bars depict one standard deviation. The shadowed zone marks loadings between −0.2 and 0.2. The data needed to generate this figure can be found in S31_Data.

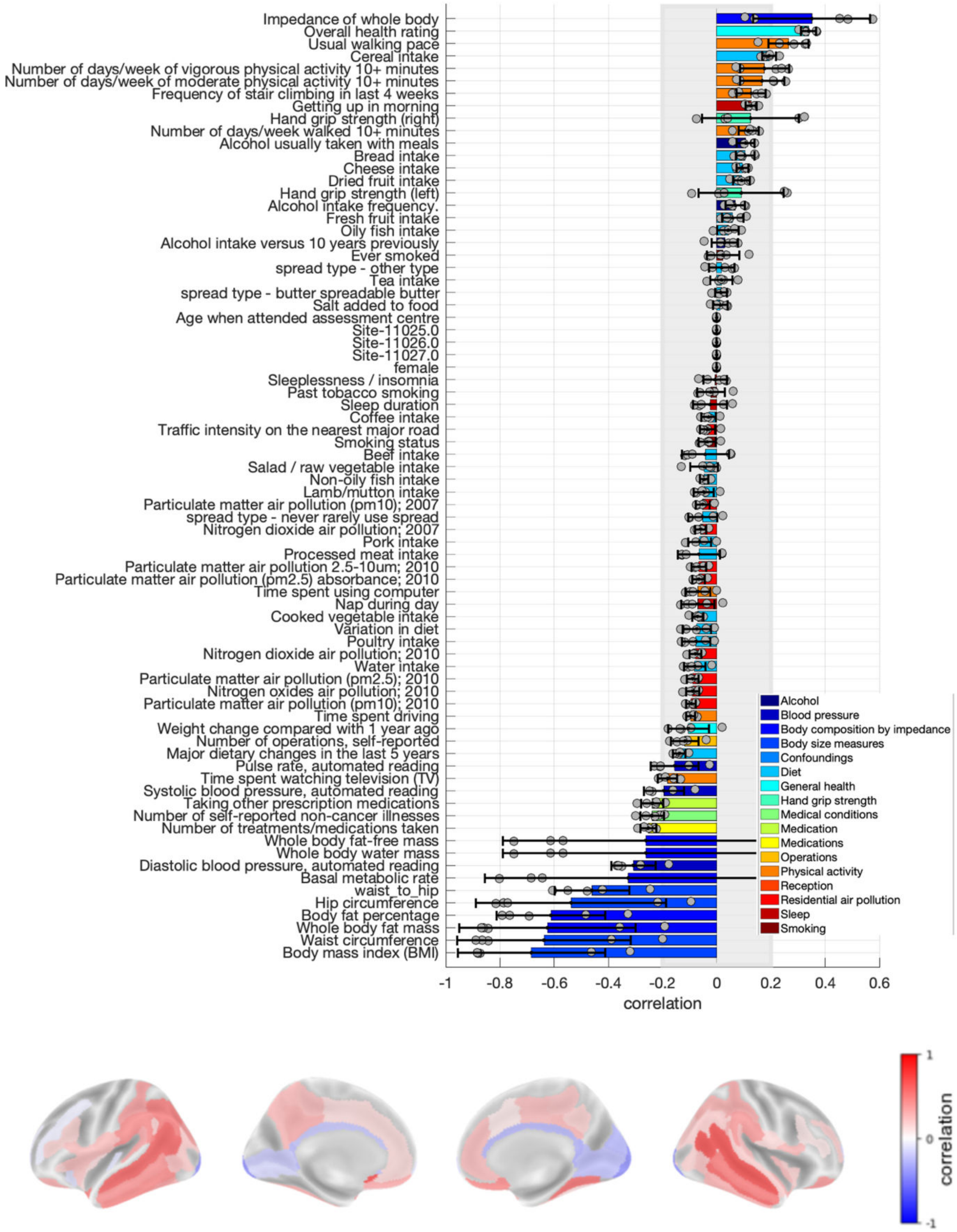

Figure P. Loadings of the latent dimension of cardiometabolic health for proportional grey matter volume in the main sample. Risk factors loadings and brain loadings. Shown loadings represent the average over the five outer splits. Error bars depict one standard deviation. The shadowed zone marks loadings between −0.2 and 0.2. The data needed to generate this figure can be found in S32_Data.

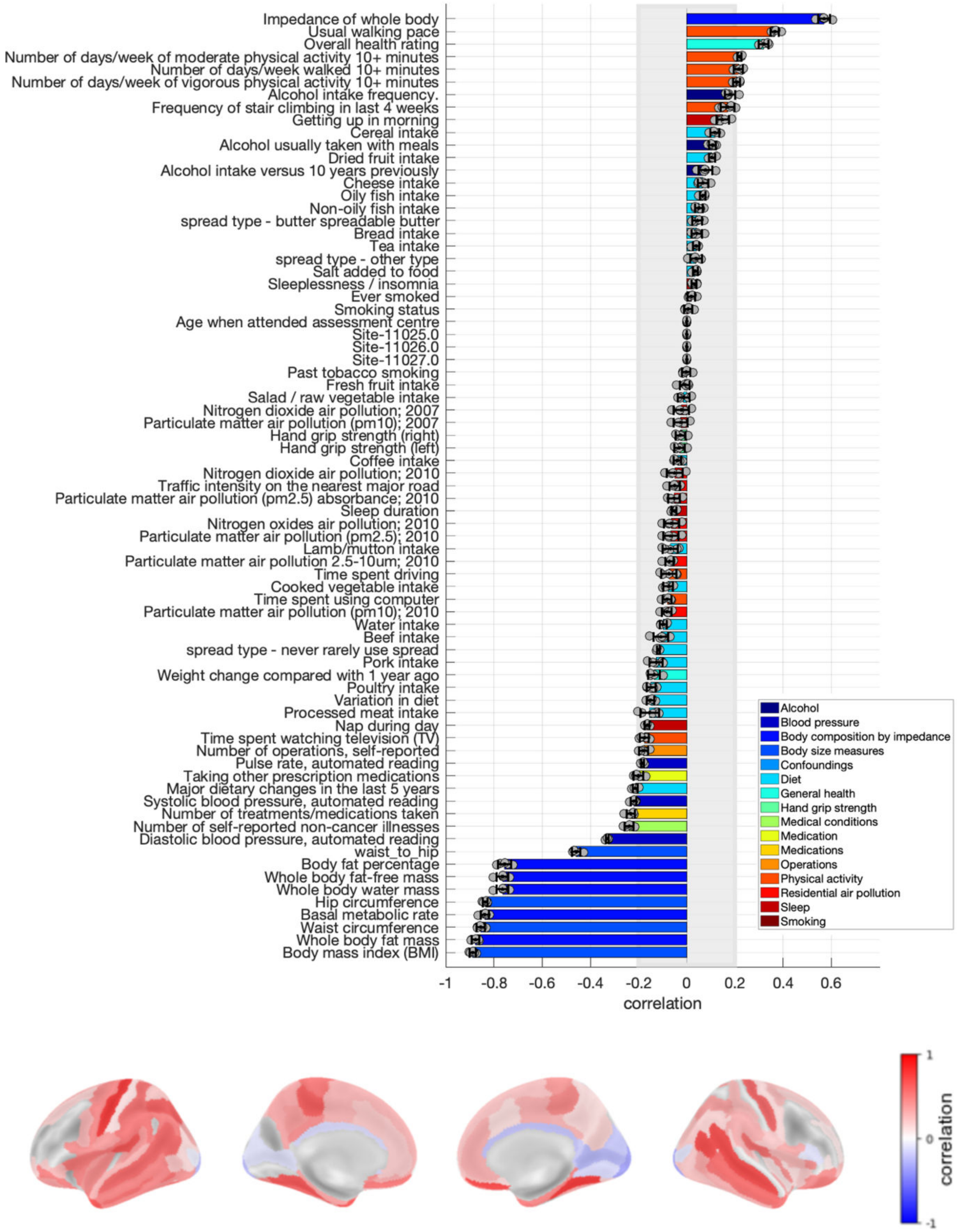

Figure Q. Loadings of the latent dimension of cardiometabolic health for proportional grey matter volume in women. Risk factors loadings and brain loadings. Shown loadings represent the average over the five outer splits. Error bars depict one standard deviation. The shadowed zone marks loadings between −0.2 and 0.2. The data needed to generate this figure can be found in S33_Data.

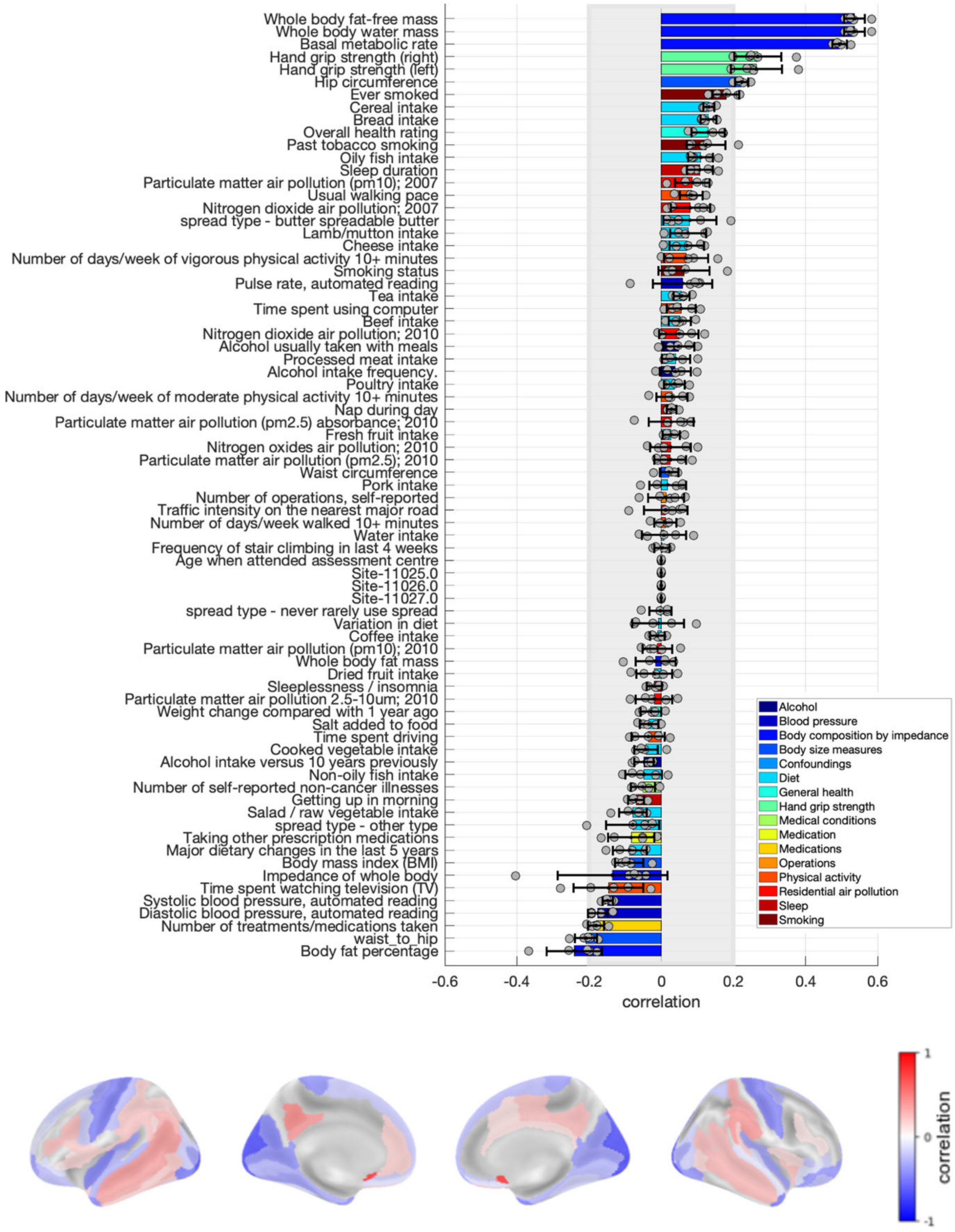

Figure R. Loadings of the latent dimension of cardiometabolic health for proportional grey matter volume in men. Risk factors loadings and brain loadings. Shown loadings represent the average over the five outer splits. Error bars depict one standard deviation. The shadowed zone marks loadings between −0.2 and 0.2. The data needed to generate this figure can be found in S34_Data.

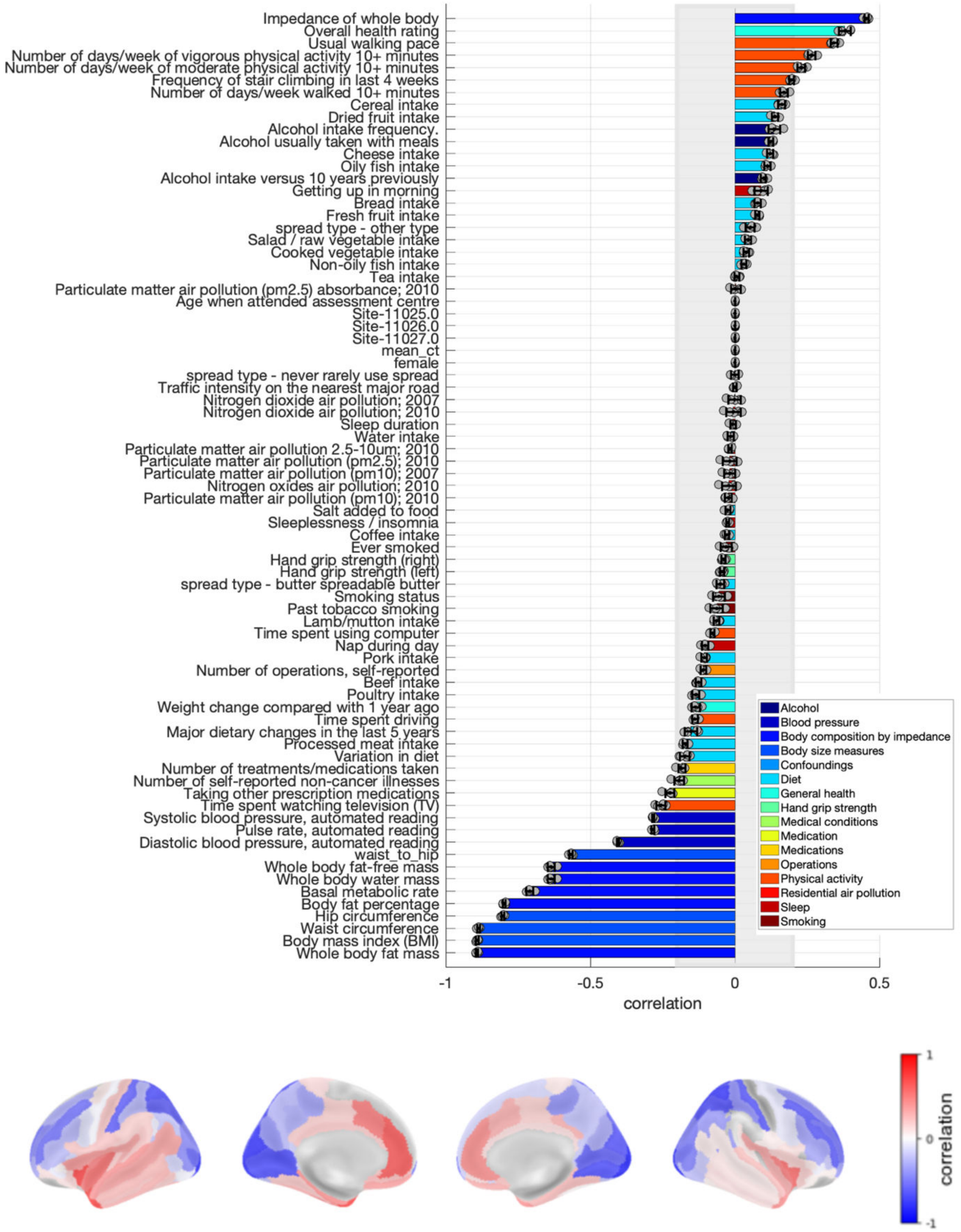

Figure S. Loadings of the latent dimension of cardiometabolic health for corrected cortical thickness in the main sample. Risk factors loadings and brain loadings. Shown loadings represent the average over the five outer splits. Error bars depict one standard deviation. The shadowed zone marks loadings between −0.2 and 0.2. The data needed to generate this figure can be found in S35_Data.

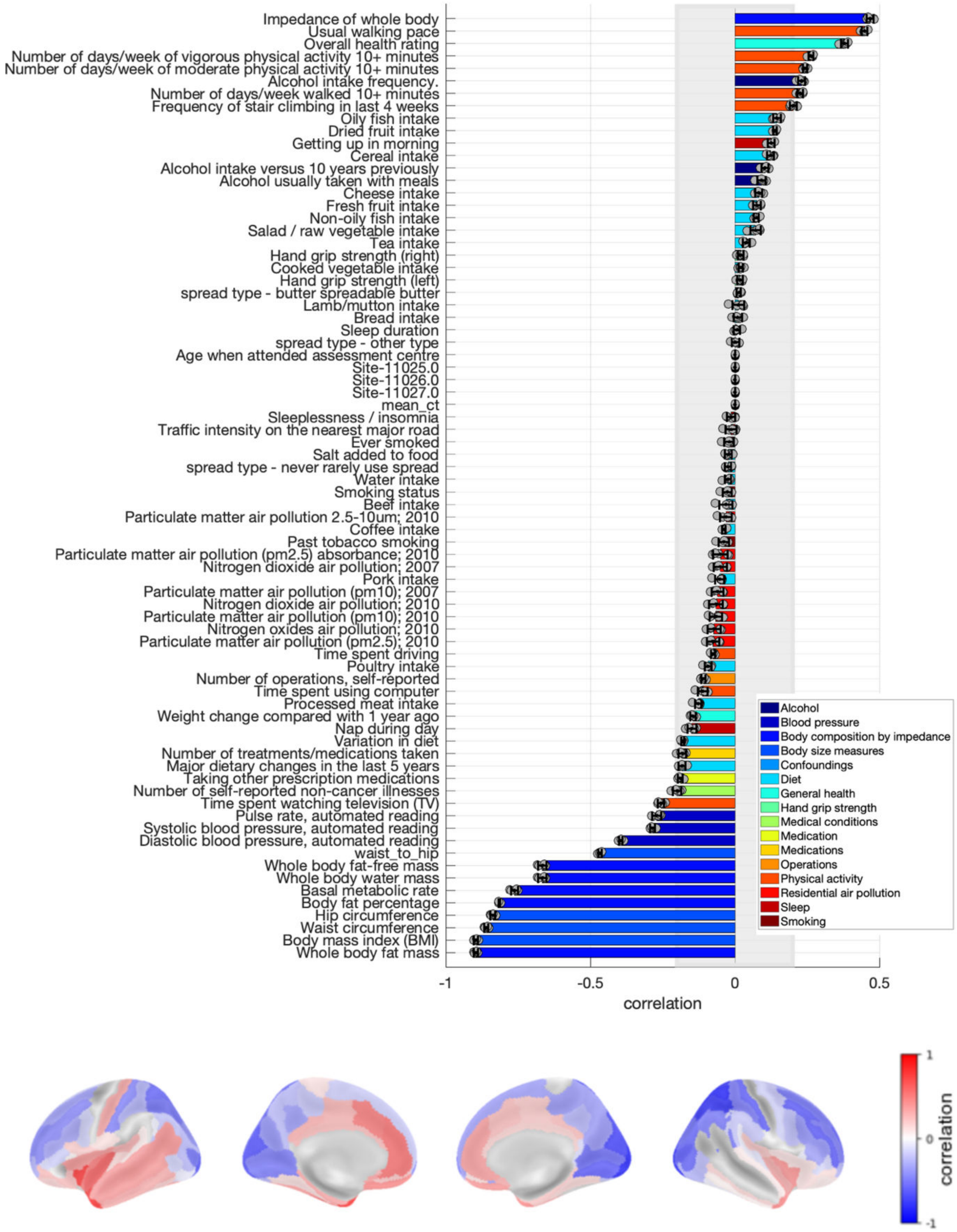

Figure T. Loadings of the latent dimension of cardiometabolic health for corrected cortical thickness in women. Risk factors loadings and brain loadings. Shown loadings represent the average over the five outer splits. Error bars depict one standard deviation. The shadowed zone marks loadings between −0.2 and 0.2. The data needed to generate this figure can be found in S36_Data.

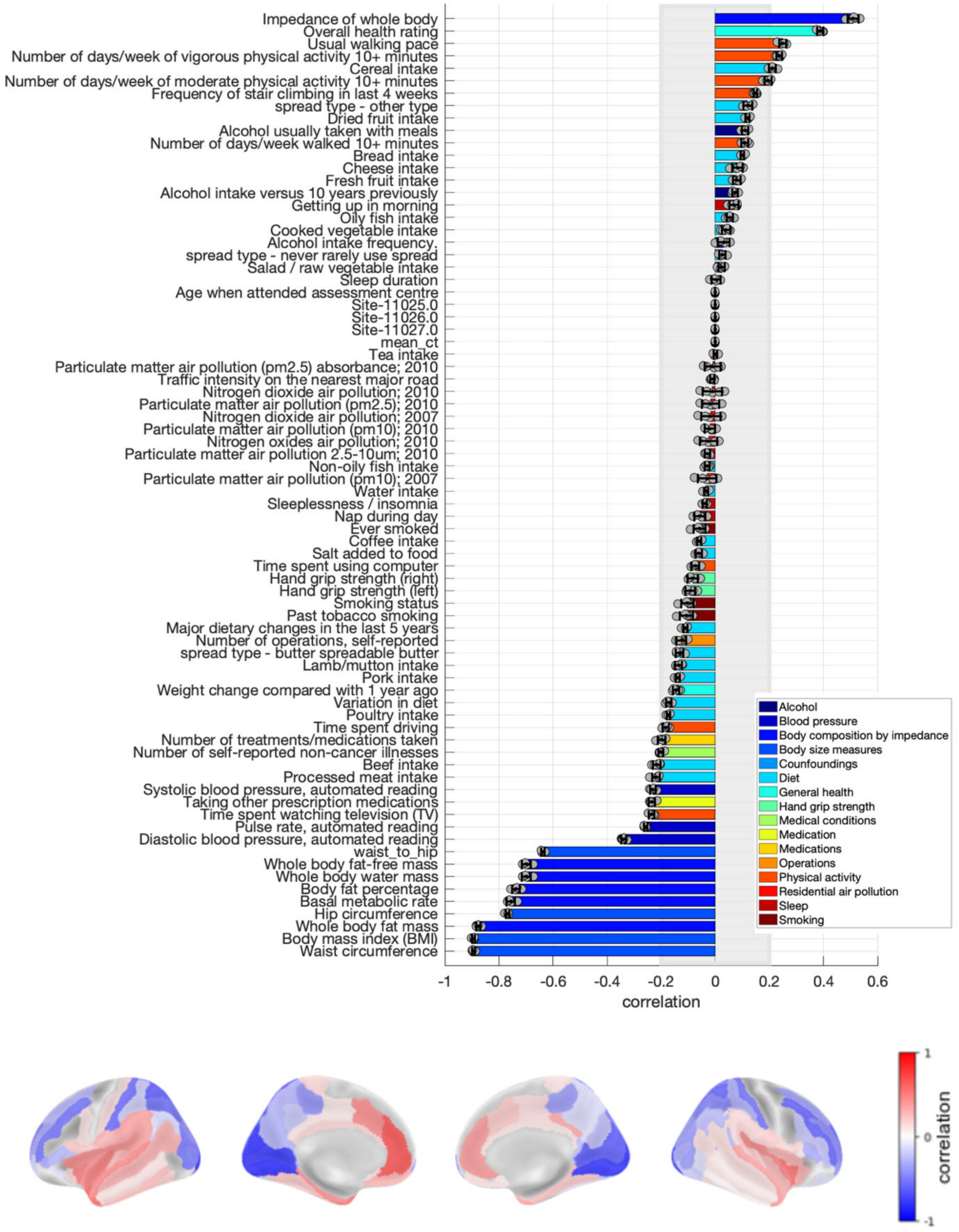

Figure U. Loadings of the latent dimension of cardiometabolic health for corrected cortical thickness in men. Risk factors loadings and brain loadings. Shown loadings represent the average over the five outer splits. Error bars depict one standard deviation. The shadowed zone marks loadings between −0.2 and 0.2. The data needed to generate this figure can be found in S37_Data.

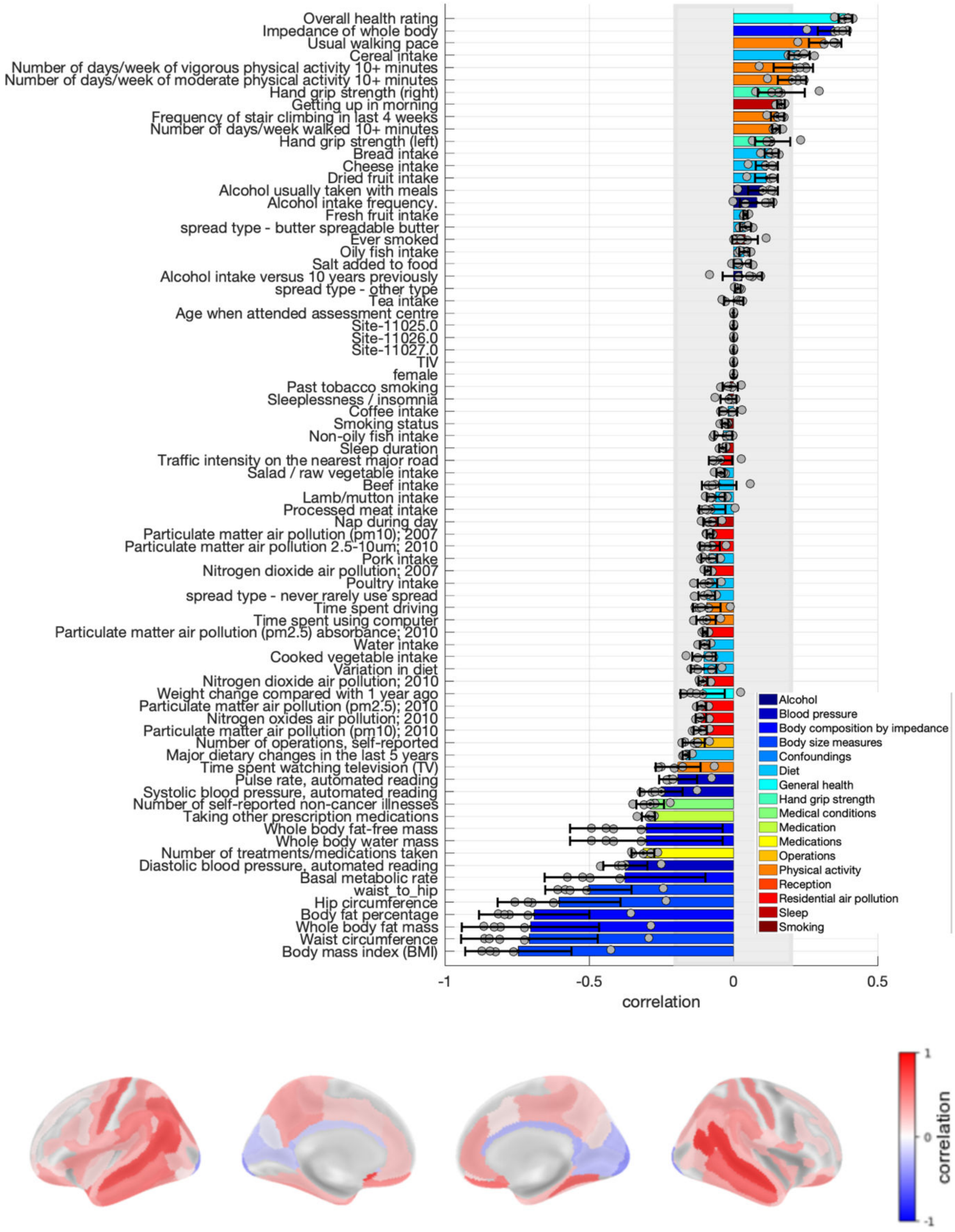

Figure V. Loadings of the latent dimension of cardiometabolic health for corrected grey matter volume in the main sample. Risk factors loadings and brain loadings. Shown loadings represent the average over the five outer splits. Error bars depict one standard deviation. The shadowed zone marks loadings between −0.2 and 0.2. The data needed to generate this figure can be found in S38_Data.

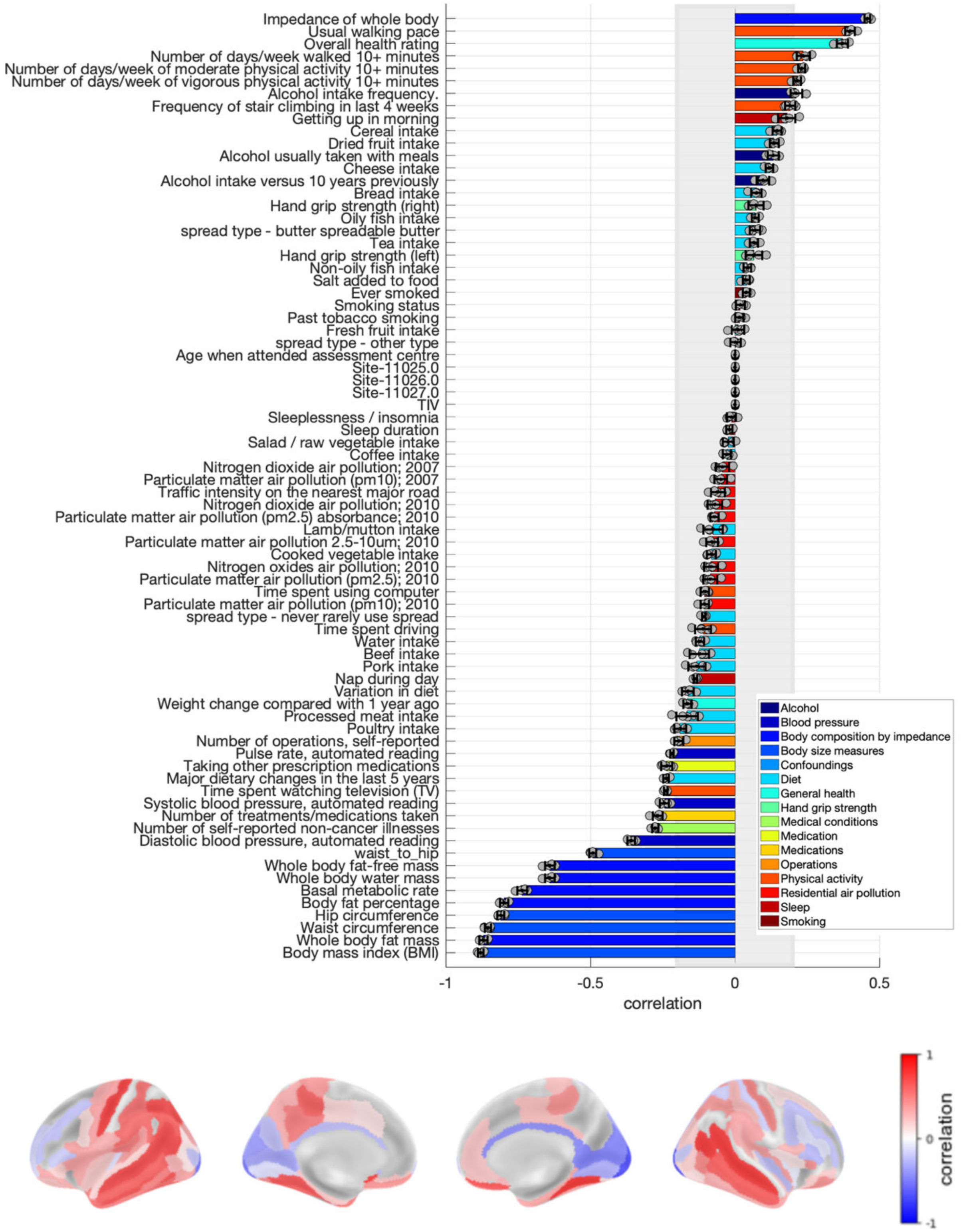

Figure W. Loadings of the latent dimension of cardiometabolic health for corrected grey matter volume in women. Risk factors loadings and brain loadings. Shown loadings represent the average over the five outer splits. Error bars depict one standard deviation. The shadowed zone marks loadings between −0.2 and 0.2. The data needed to generate this figure can be found in S39_Data.

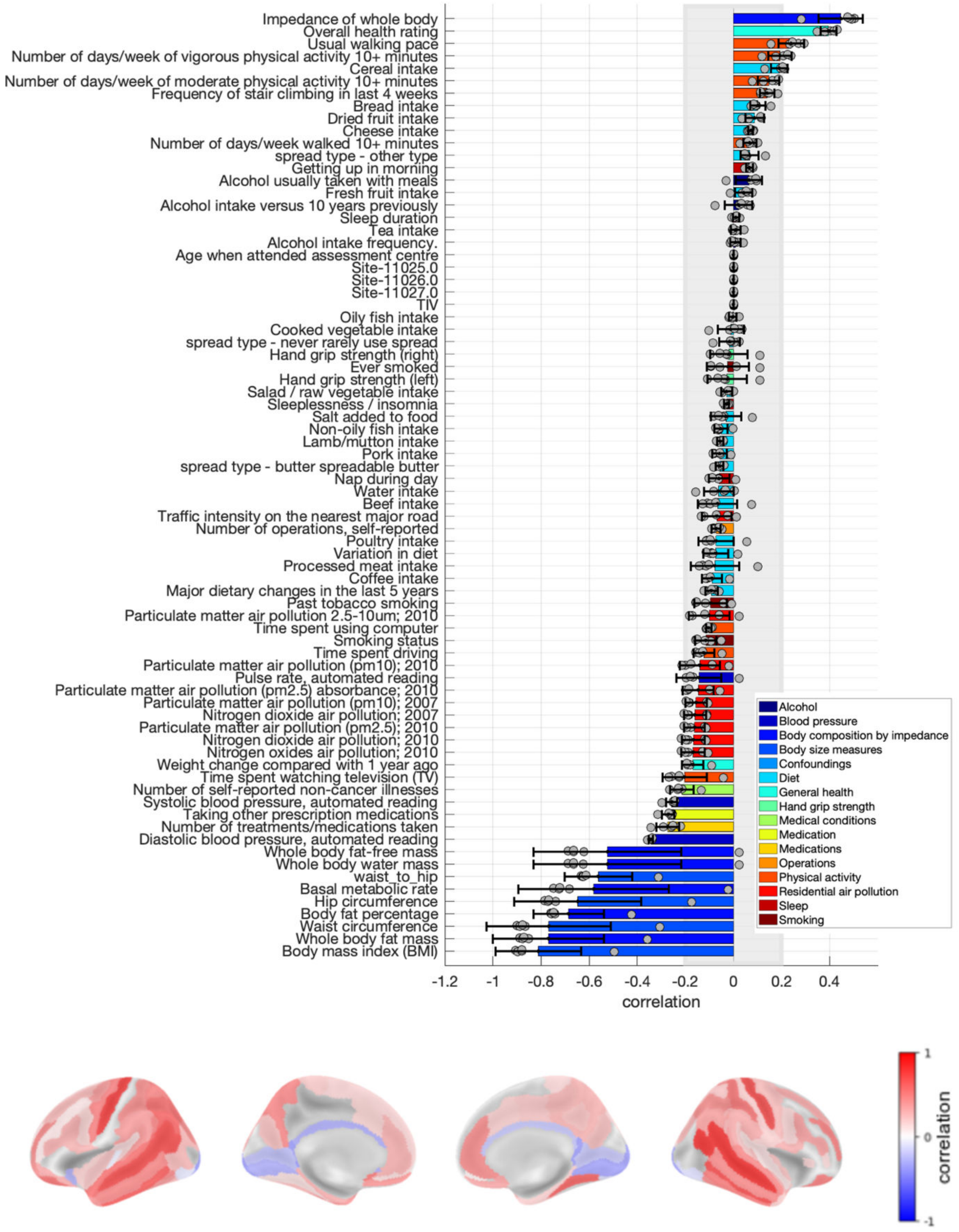

Figure X. Loadings of the latent dimension of cardiometabolic health for corrected grey matter volume in men. Risk factors loadings and brain loadings. Shown loadings represent the average over the five outer splits. Error bars depict one standard deviation. The shadowed zone marks loadings between −0.2 and 0.2. The data needed to generate this figure can be found in S40_Data.

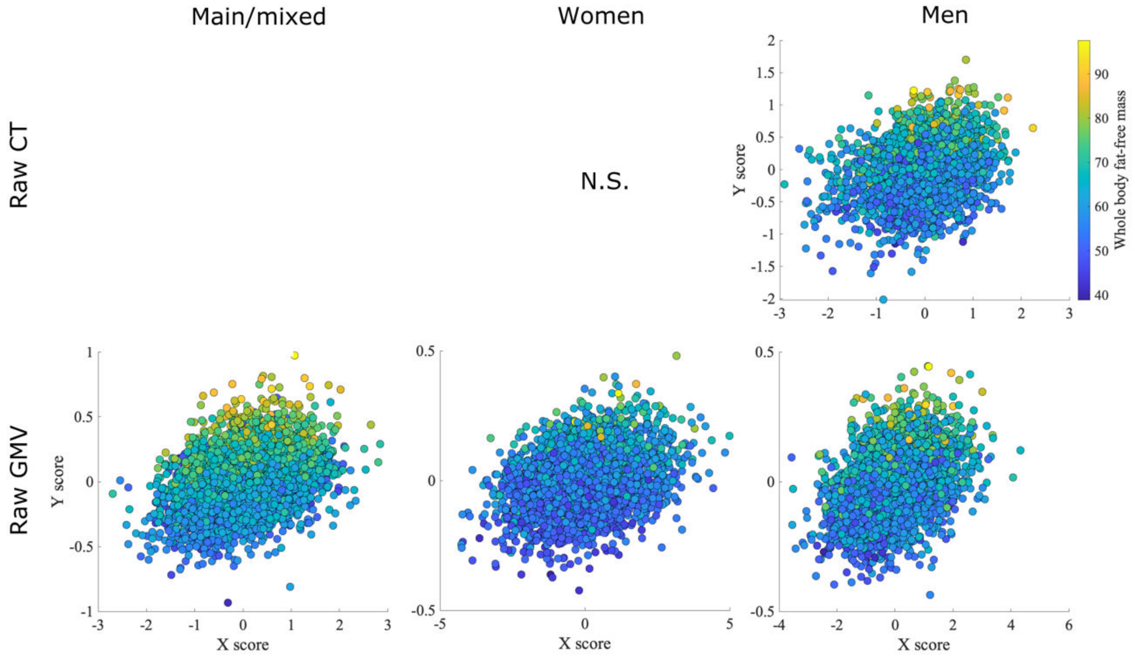

Figure Y. Latent dimension of physical robustness. Each scatterplot shows the brain structural (X) and risk factors (Y) scores averaged over the splits for each model. Each dot represents one participant. CT: cortical thickness. GMV: grey matter volume

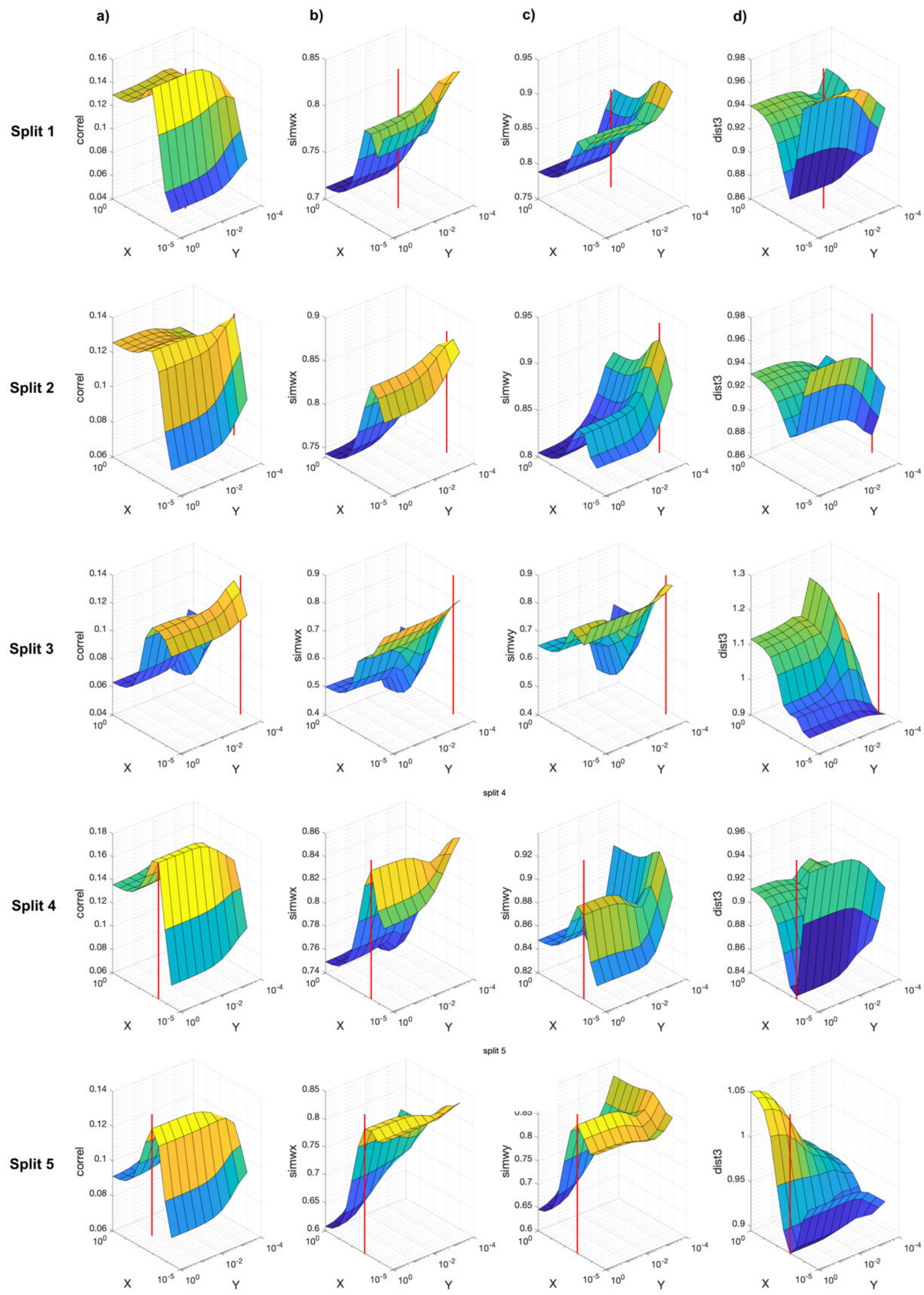

Figure Z. Model optimization for the latent dimension of physical robustness for raw cortical thickness in the sample of men. The red line indicates the selected model. The z axis represents the test canonical correlation (column a), the similarity of weights in cortical thickness (column b) and risk factors (column c), and the joint generalizability-stability criteria (column d). The x and y axes represent 1 minus the hyperparameters tested for cortical thickness and risk factors, respectively (1-hyperparameter). The x and y axes are shown in logarithmic scale. The data needed to generate this figure can be found in S41_Data (split 1) S42_Data (split 2), S43_Data (split 3), S44_Data (split 4) and S45_Data (split 5).

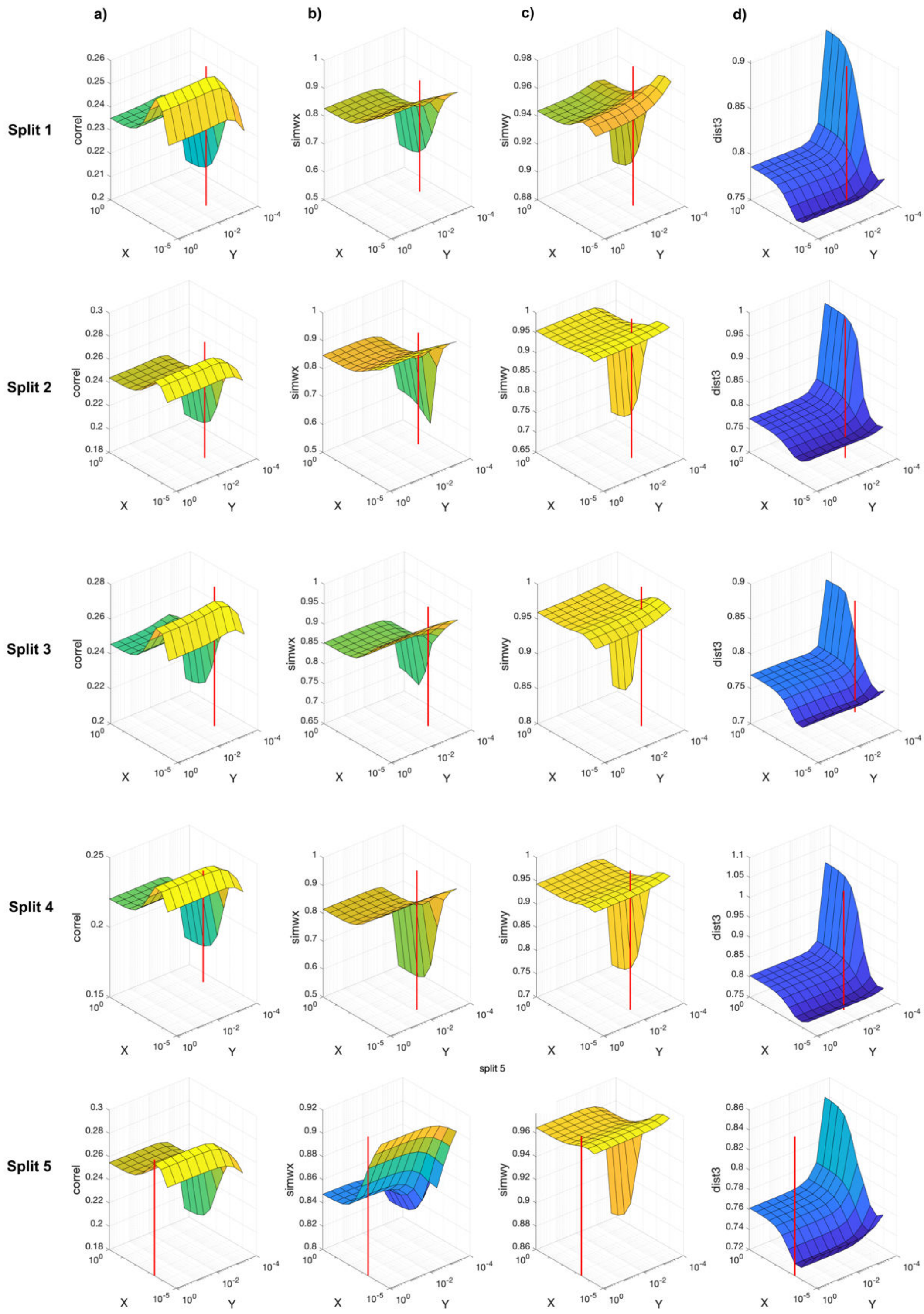

Figure AA. Model optimization for the latent dimension of physical robustness for raw grey matter volume in the main sample. The red line indicates the selected model. The z axis represents the test canonical correlation (column a), the similarity of weights in grey matter volume (column b) and risk factors (column c), and the joint generalizability-stability criteria (column d). The x and y axes represent 1-minus the hyperparameters tested for grey matter volume and risk factors, respectively (1-hyperparameter). The x and y axes are shown in logarithmic scale. The data needed to generate this figure can be found in S46_Data (split 1) S47_Data (split 2), S48_Data (split 3), S49_Data (split 4) and S50_Data (split 5).

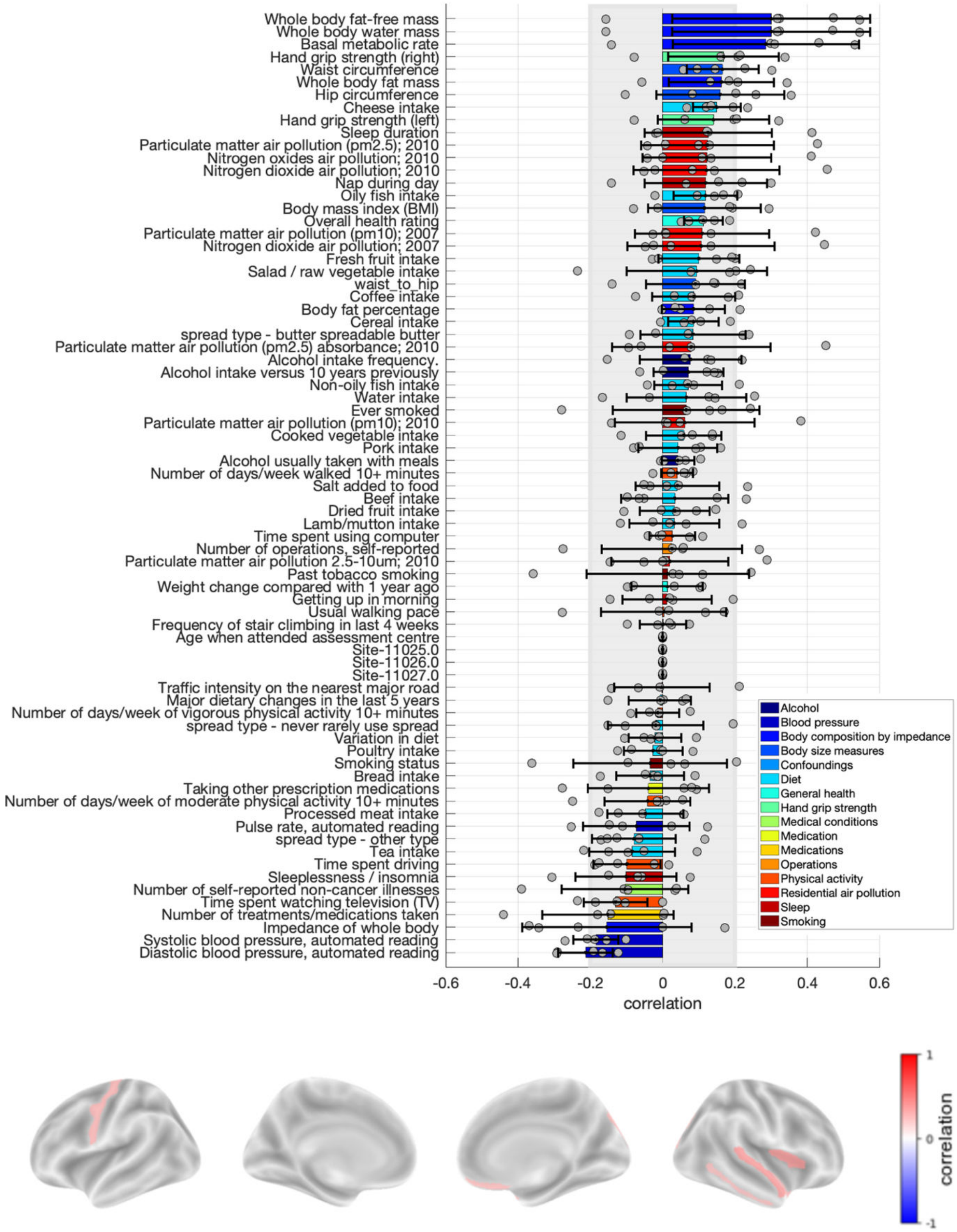

Figure AB. Loadings of the latent dimension of physical robustness for raw cortical thickness in women. Risk factors loadings and brain loadings. Shown loadings represent the average over the five outer splits. Error bars depict one standard deviation. The shadowed zone marks loadings between −0.2 and 0.2. Note that this latent dimension was significant only in one split out of five. The data needed to generate this figure can be found in S51_Data.

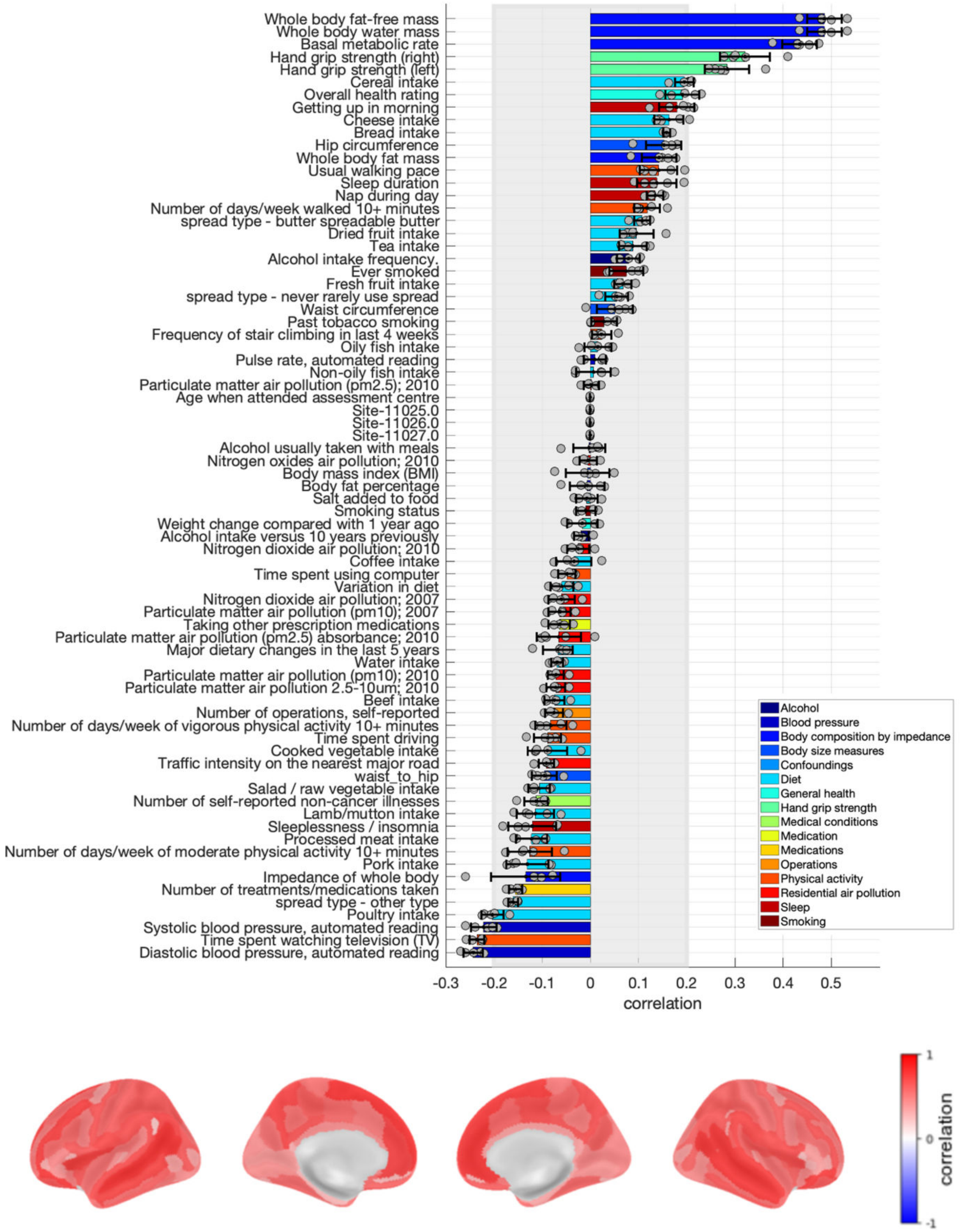

Figure AC. Loadings of the latent dimension of physical robustness for raw grey matter volume in women. Risk factors loadings and brain loadings. Shown loadings represent the average over the five outer splits. Error bars depict one standard deviation. The shadowed zone marks loadings between −0.2 and 0.2. The data needed to generate this figure can be found in S52_Data.

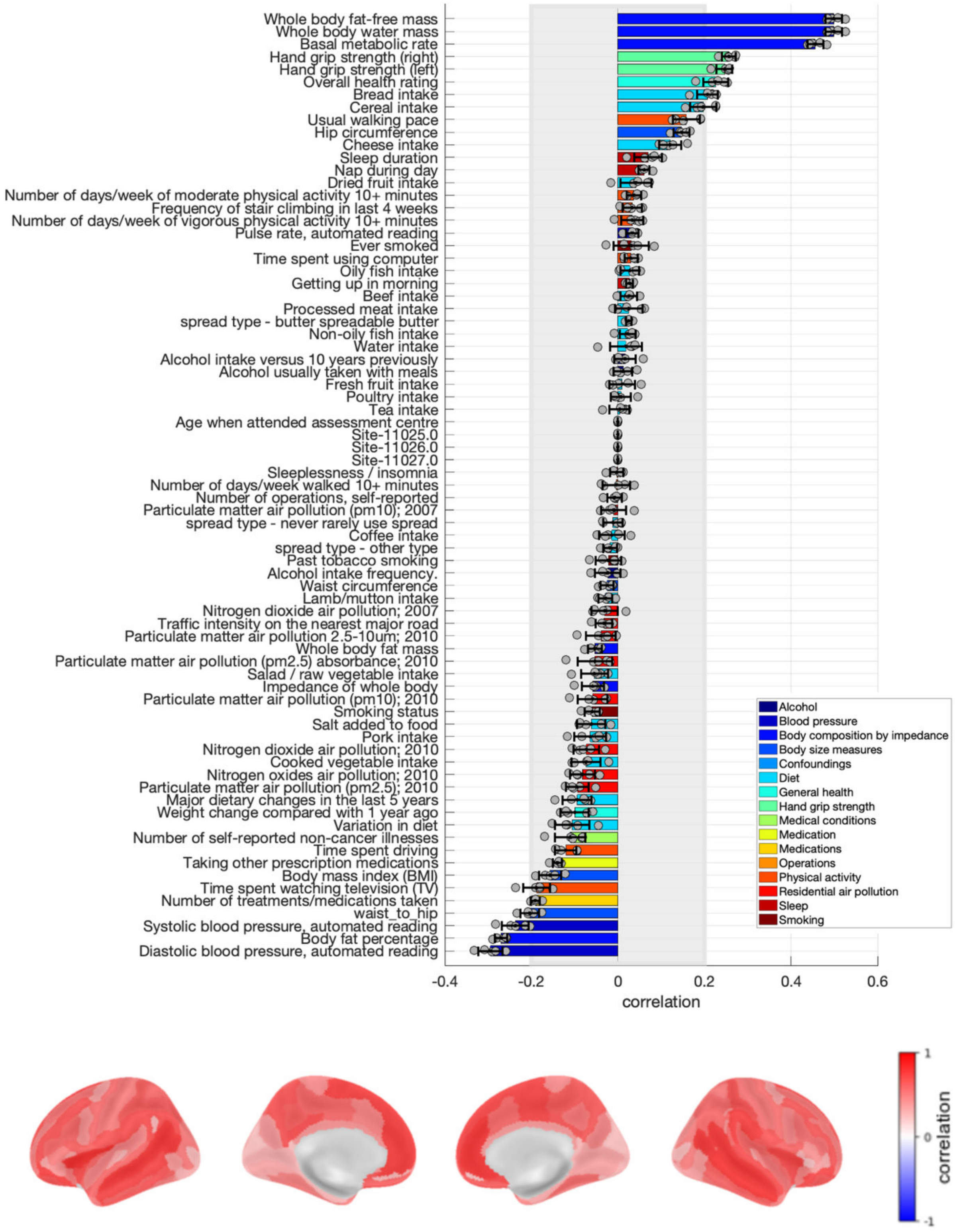

Figure AD. Loadings of the latent dimension of physical robustness for raw grey matter volume in men. Risk factors loadings and brain loadings. Shown loadings represent the average over the five outer splits. Error bars depict one standard deviation. The shadowed zone marks loadings between −0.2 and 0.2. The data needed to generate this figure can be found in S53_Data.

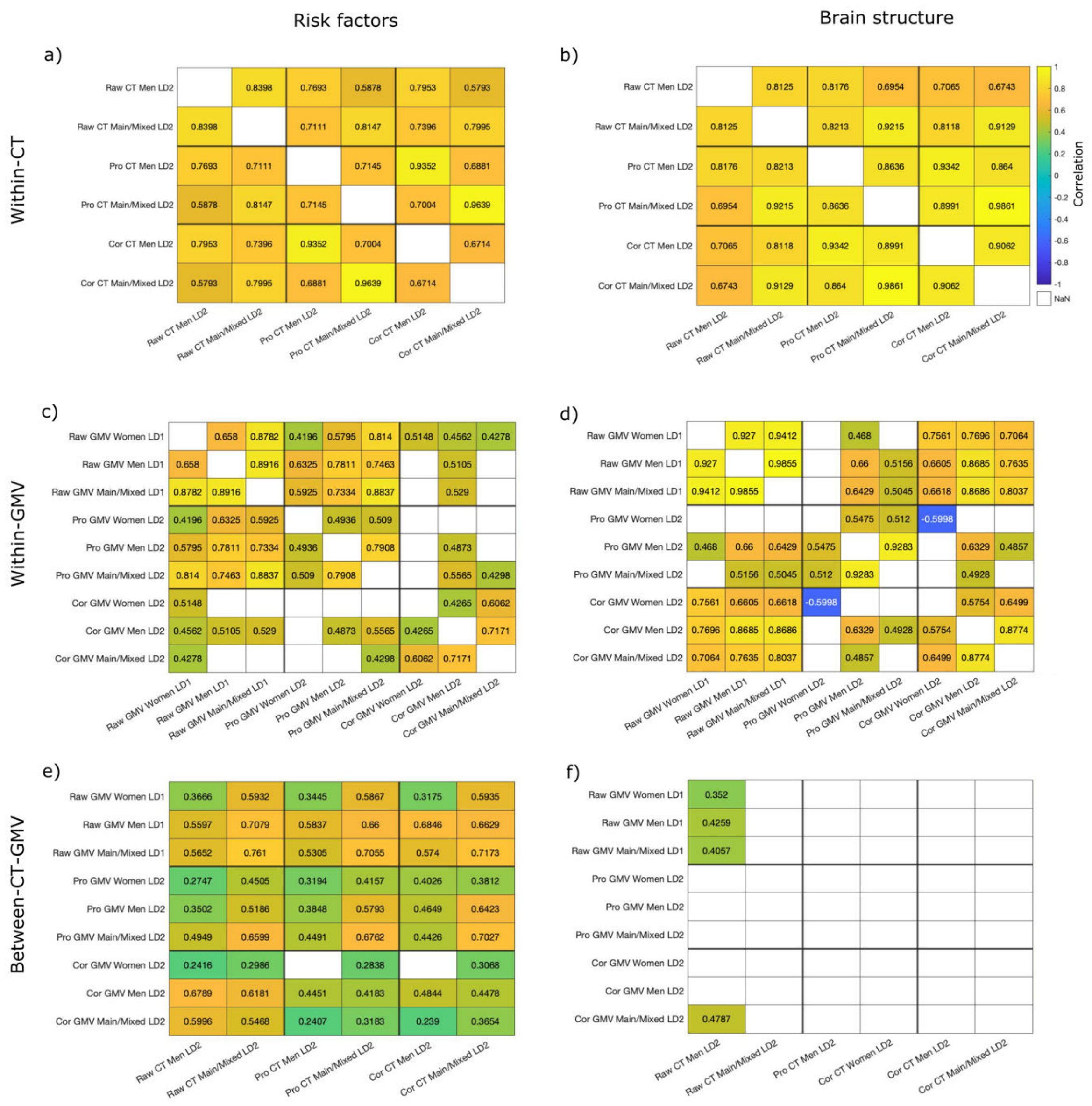

Figure AE. Comparison of risk factor loadings and brain loadings for the physical robustness latent dimension across samples and brain structural measures. The first row shows the Spearman correlation of a) risk factors loadings and b) brain loadings for the latent dimension of physical robustness yielded with cortical thickness. The second row shows the correlation of c) risk factors loadings and d) brain loadings for the latent dimension of physical robustness yielded with grey matter volume. Only comparisons that were significant after Bonferroni correction are shown. The data needed to generate this figure can be found in S54-S59_Data.

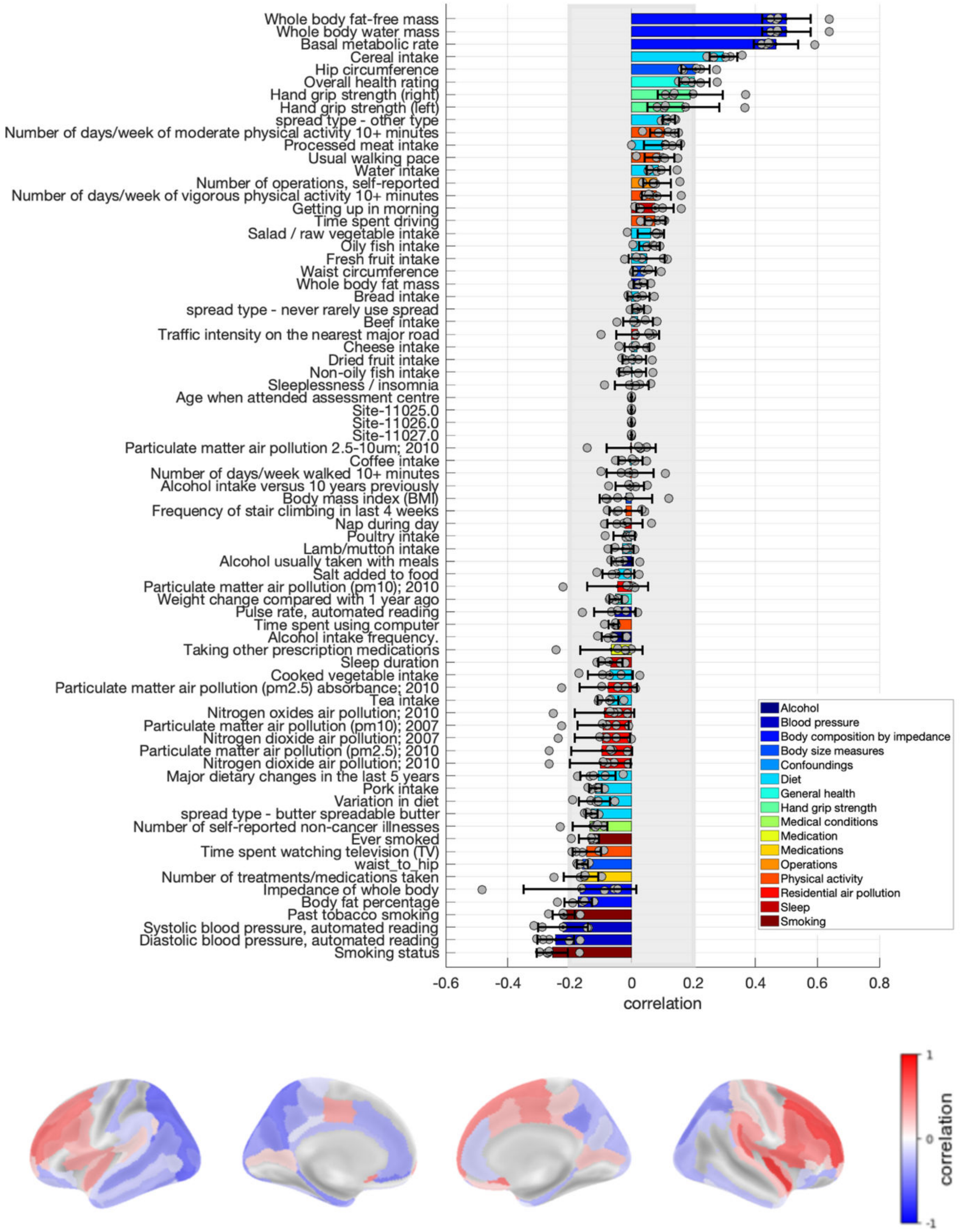

Figure AF. Loadings of the latent dimension of physical robustness for proportional cortical thickness in men. Risk factors loadings and brain loadings. Shown loadings represent the average over the five outer splits. Error bars depict one standard deviation. The shadowed zone marks loadings between −0.2 and 0.2. The data needed to generate this figure can be found in S60_Data.

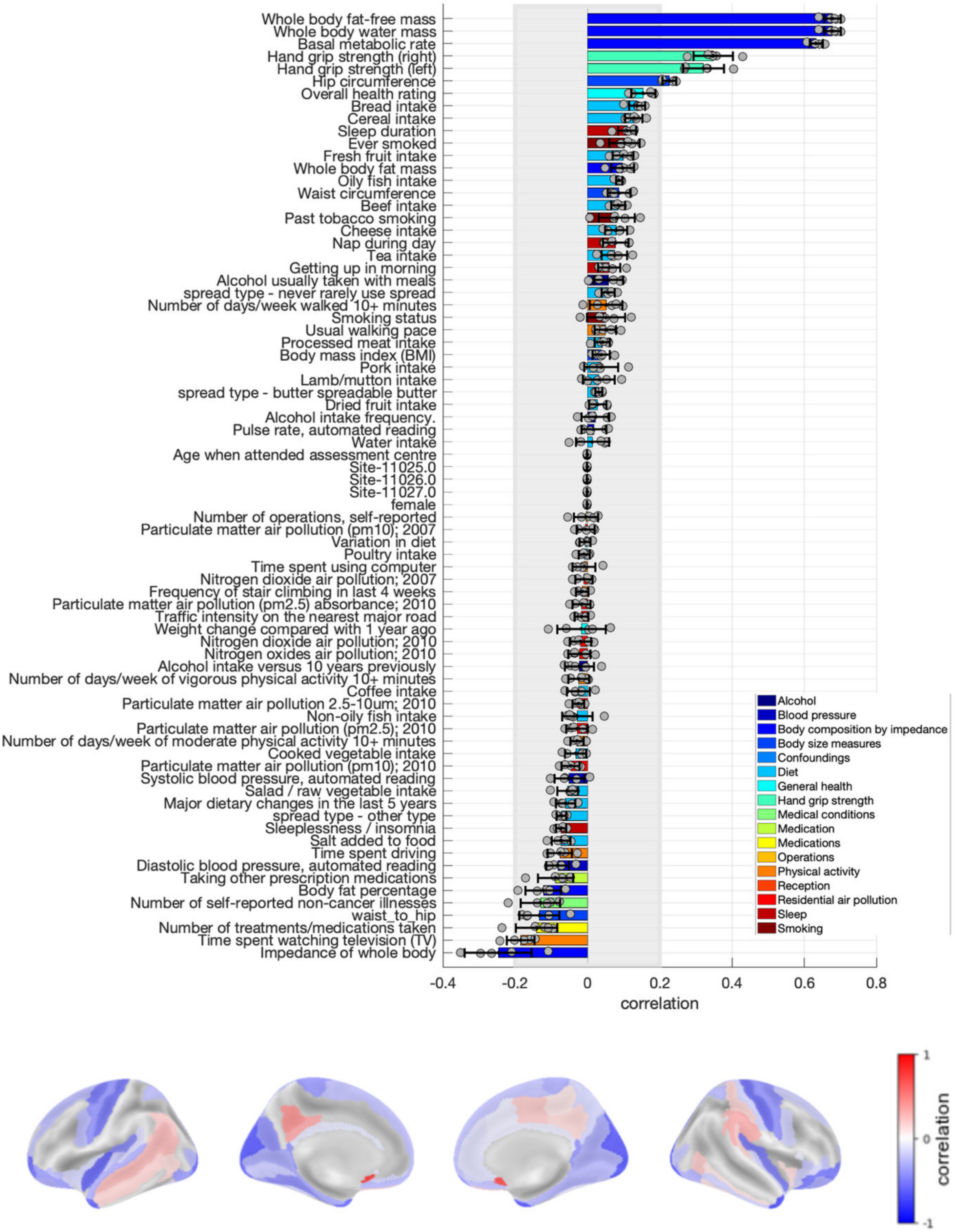

Figure AG. Loadings of the latent dimension of physical robustness for proportional grey matter volume in the main sample. Risk factors loadings and brain loadings. Shown loadings represent the average over the five outer splits. Error bars depict one standard deviation. The shadowed zone marks loadings between −0.2 and 0.2. The data needed to generate this figure can be found in S61_Data.

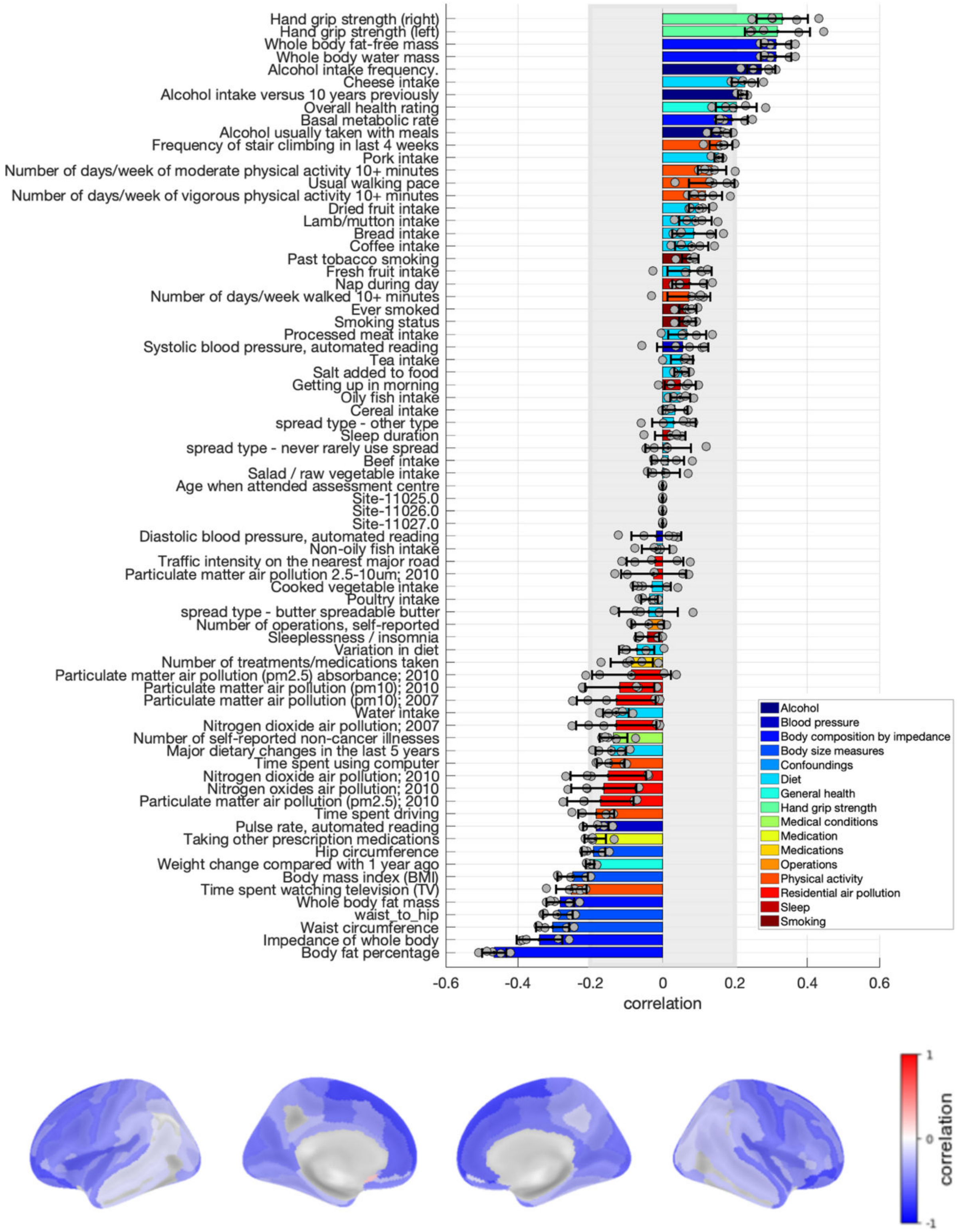

Figure AH. Loadings of the latent dimension of physical robustness for proportional grey matter volume in women. Risk factors loadings and brain loadings. Shown loadings represent the average over the five outer splits. Error bars depict one standard deviation. The shadowed zone marks loadings between −0.2 and 0.2. The data needed to generate this figure can be found in S62_Data.

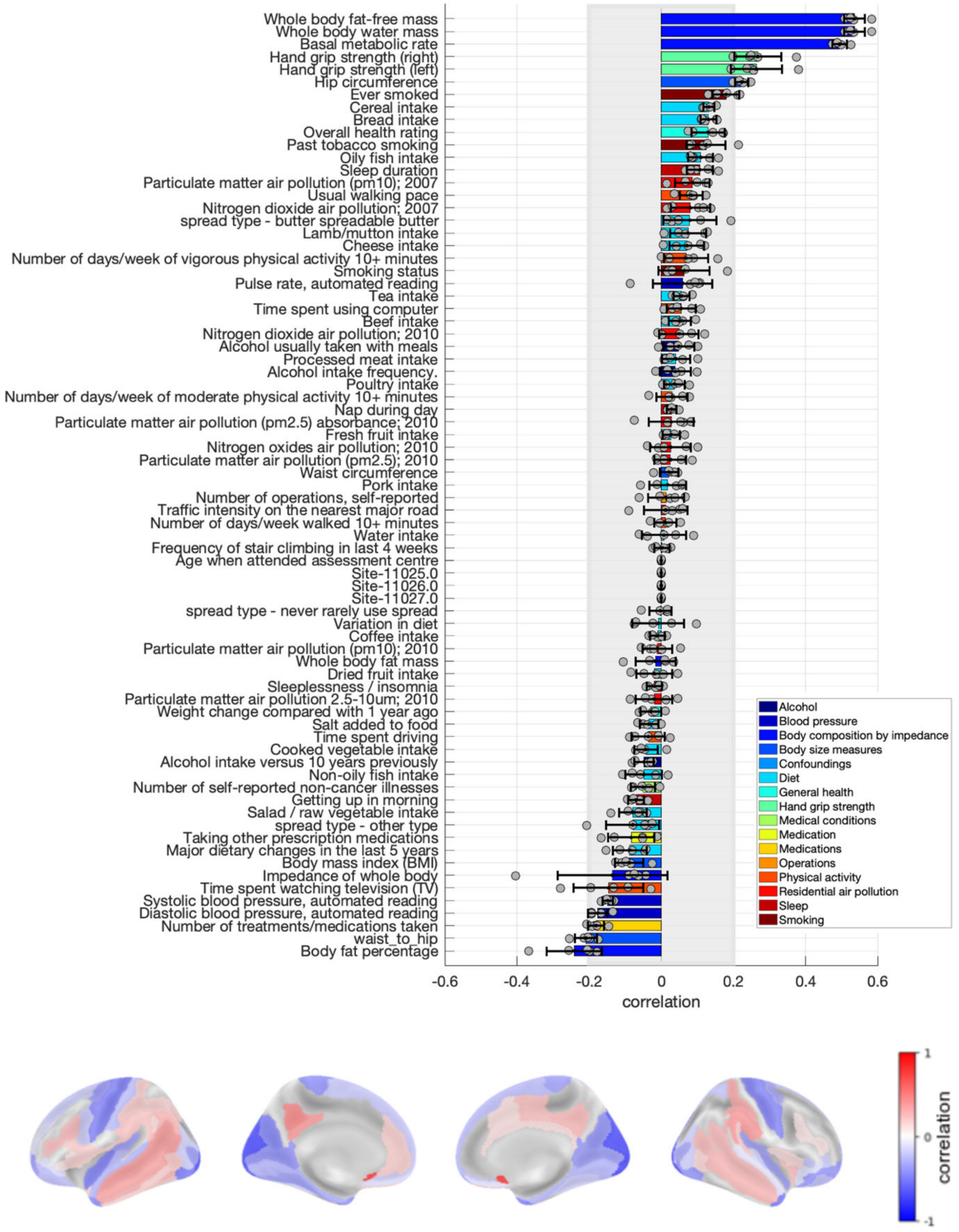

Figure AI. Loadings of the latent dimension of physical robustness for proportional grey matter volume in men. Risk factors loadings and brain loadings. Shown loadings represent the average over the five outer splits. Error bars depict one standard deviation. The shadowed zone marks loadings between −0.2 and 0.2. The data needed to generate this figure can be found in S63_Data.

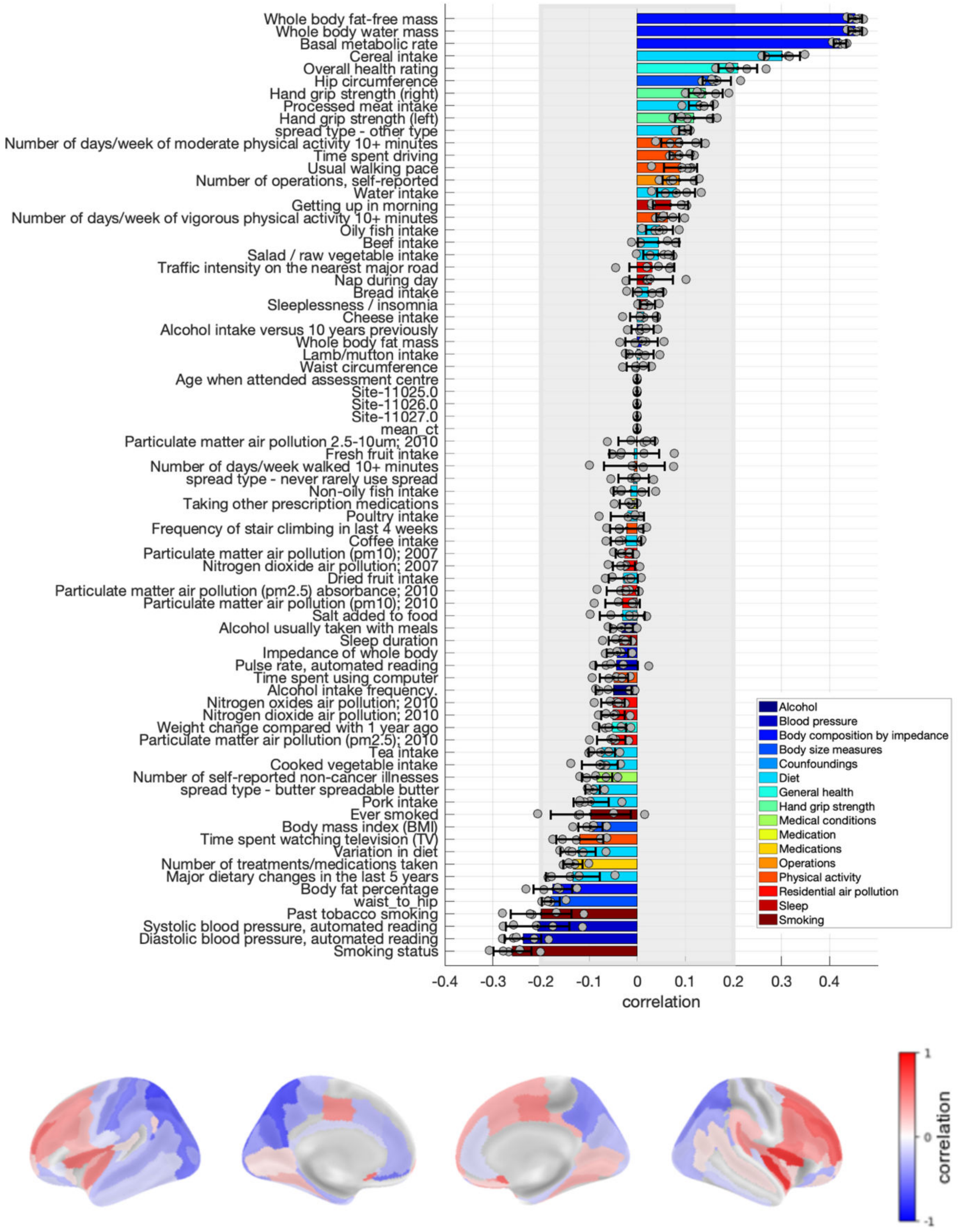

Figure AJ. Loadings of the latent dimension of physical robustness for corrected cortical thickness in men. Risk factors loadings and brain loadings. Shown loadings represent the average over the five outer splits. Error bars depict one standard deviation. The shadowed zone marks loadings between −0.2 and 0.2. The data needed to generate this figure can be found in S64_Data.

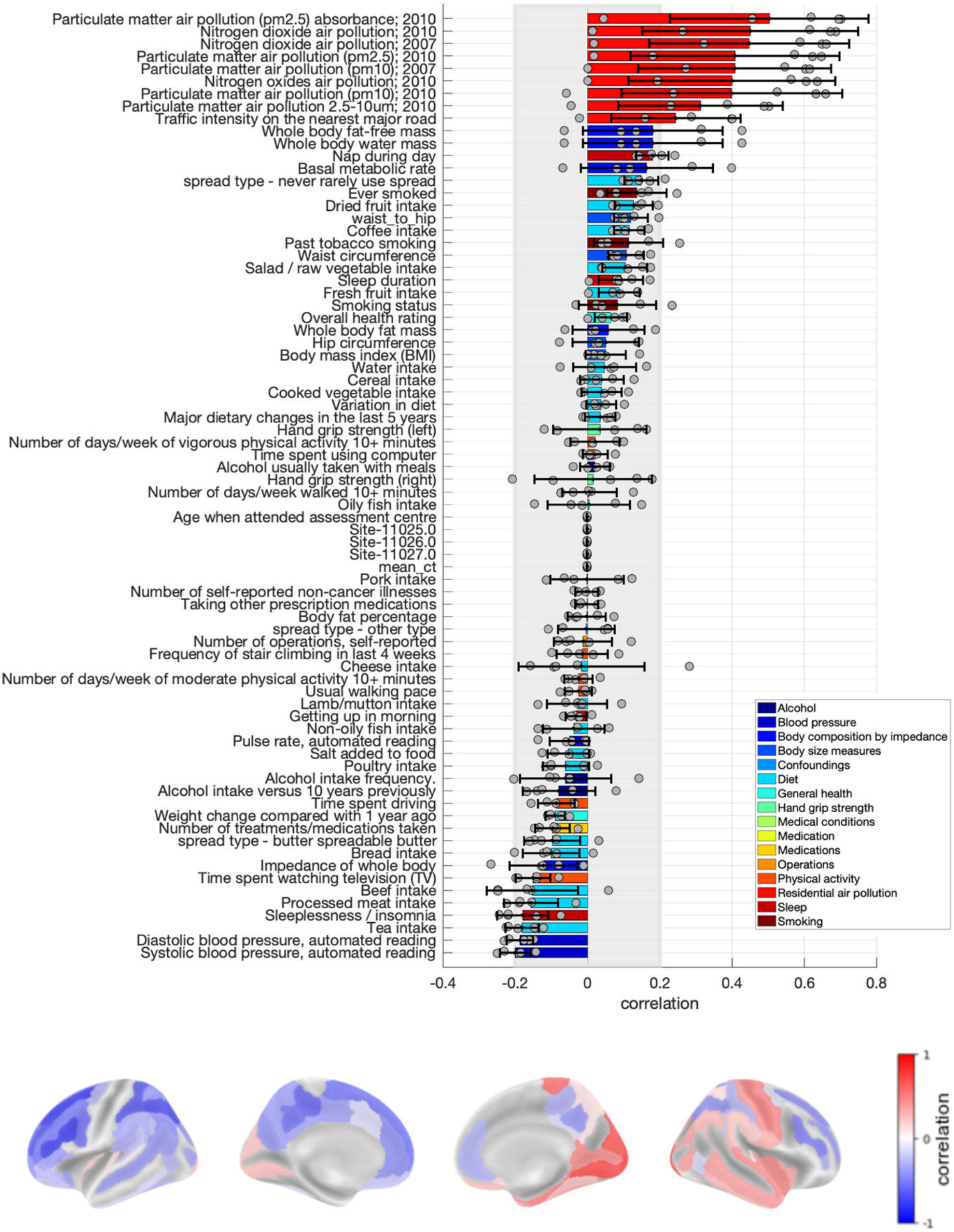

Figure AK. Loadings of the latent dimension of physical robustness for corrected cortical thickness in women. Risk factors loadings and brain loadings. Shown loadings represent the average over the five outer splits. Error bars depict one standard deviation. The shadowed zone marks loadings between −0.2 and 0.2. Note that this latent dimension was significant only in one split out of five. The data needed to generate this figure can be found in S65_Data.

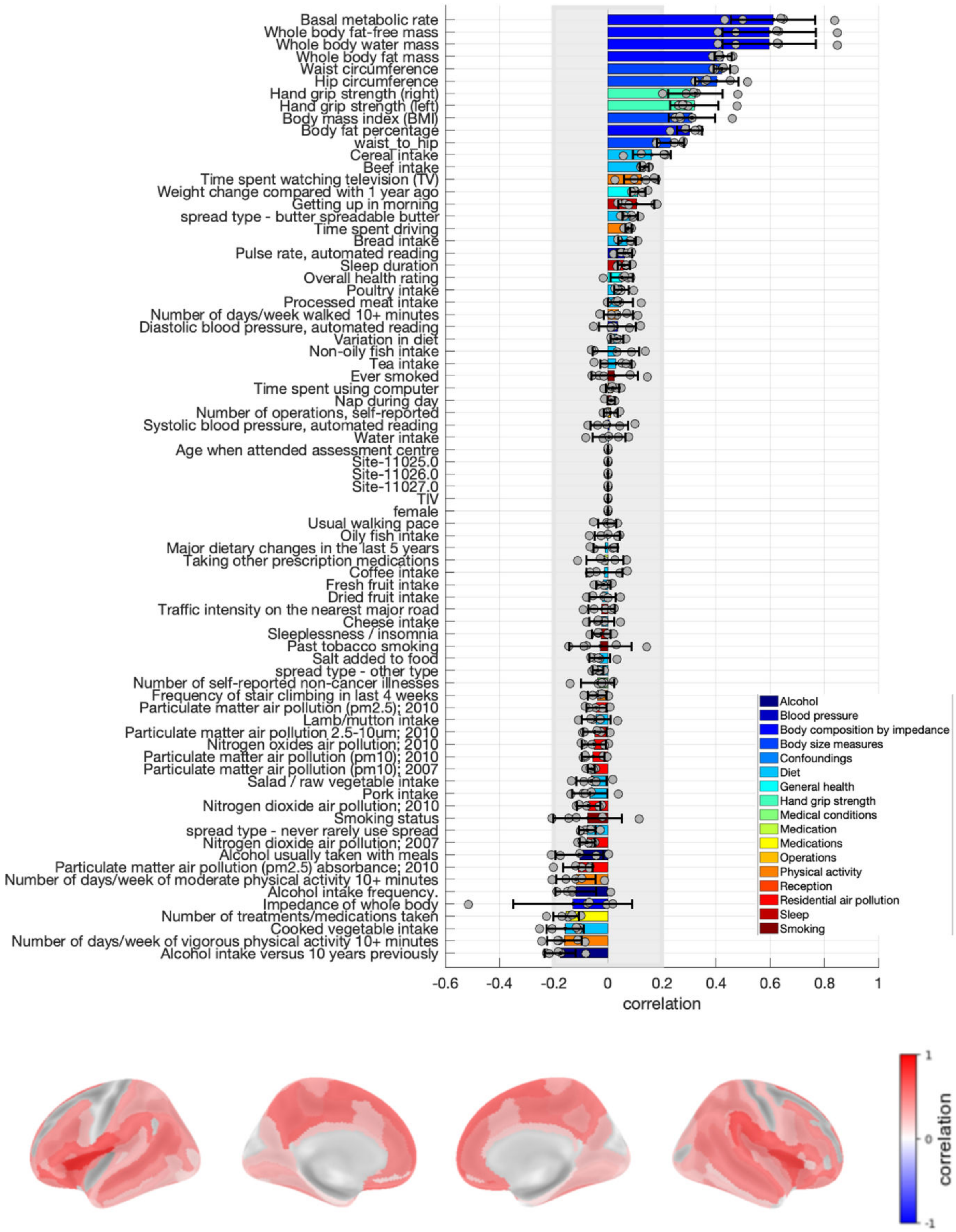

Figure AL. Loadings of the latent dimension of physical robustness for corrected grey matter volume in the main sample. Risk factors loadings and brain loadings. Shown loadings represent the average over the five outer splits. Error bars depict one standard deviation. The shadowed zone marks loadings between −0.2 and 0.2. The data needed to generate this figure can be found in S66_Data.

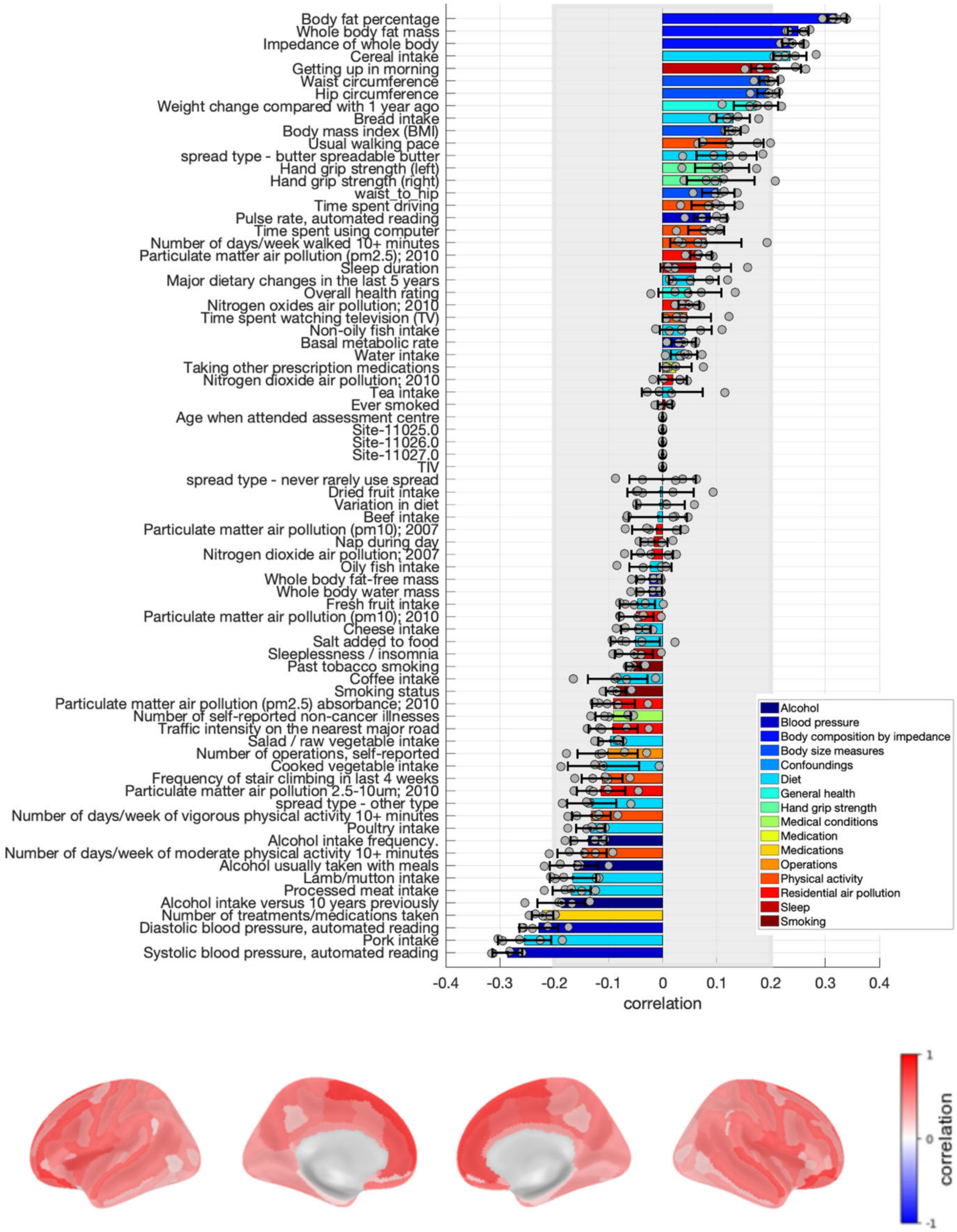

Figure AM. Loadings of the latent dimension of physical robustness for corrected grey matter volume in women. Risk factors loadings and brain loadings. Shown loadings represent the average over the five outer splits. Error bars depict one standard deviation. The shadowed zone marks loadings between −0.2 and 0.2. The data needed to generate this figure can be found in S67_Data.

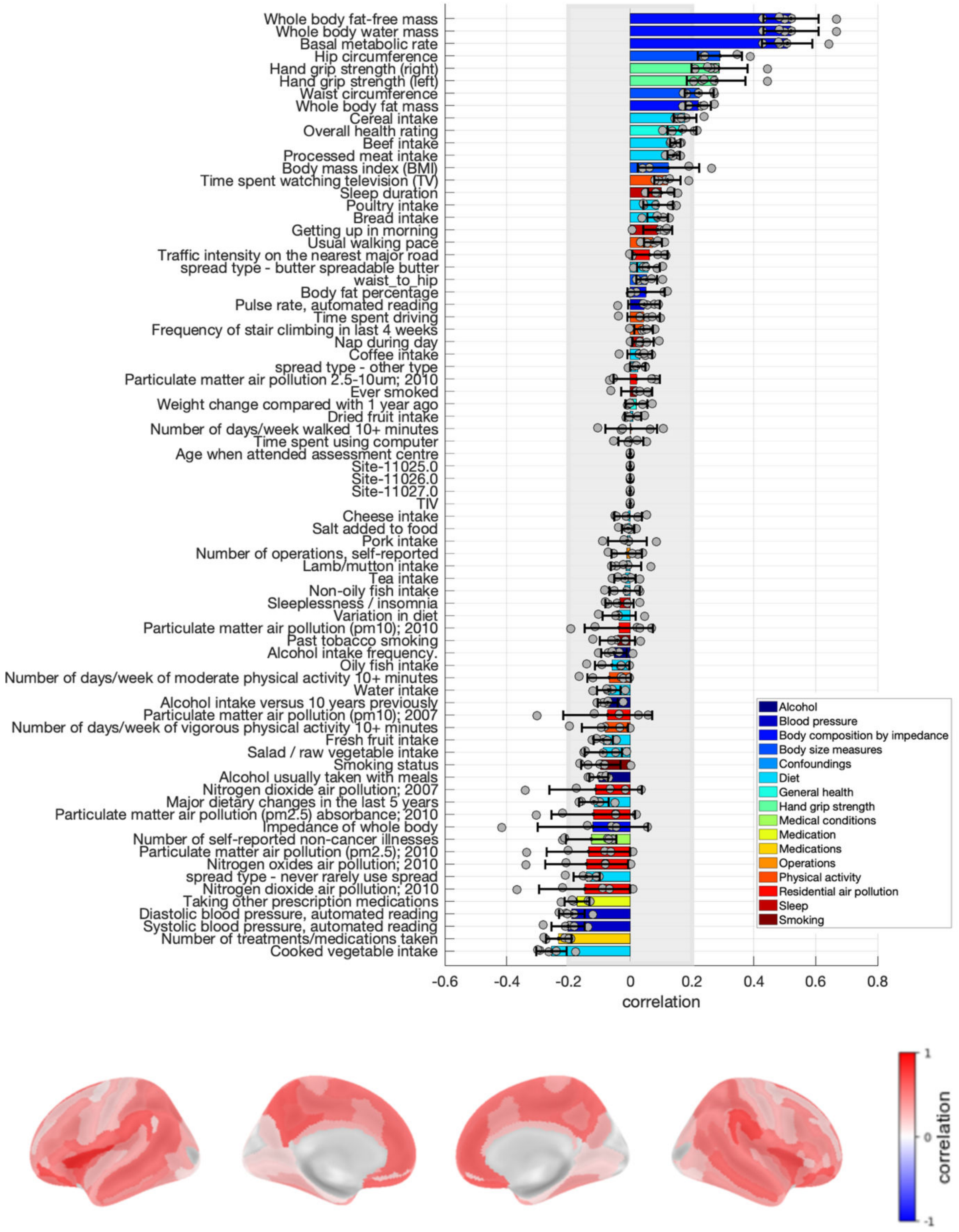

Figure AN. Loadings of the latent dimension of physical robustness for corrected grey matter volume in men. Risk factors loadings and brain loadings. Shown loadings represent the average over the five outer splits. Error bars depict one standard deviation. The shadowed zone marks loadings between −0.2 and 0.2. The data needed to generate this figure can be found in S68_Data.

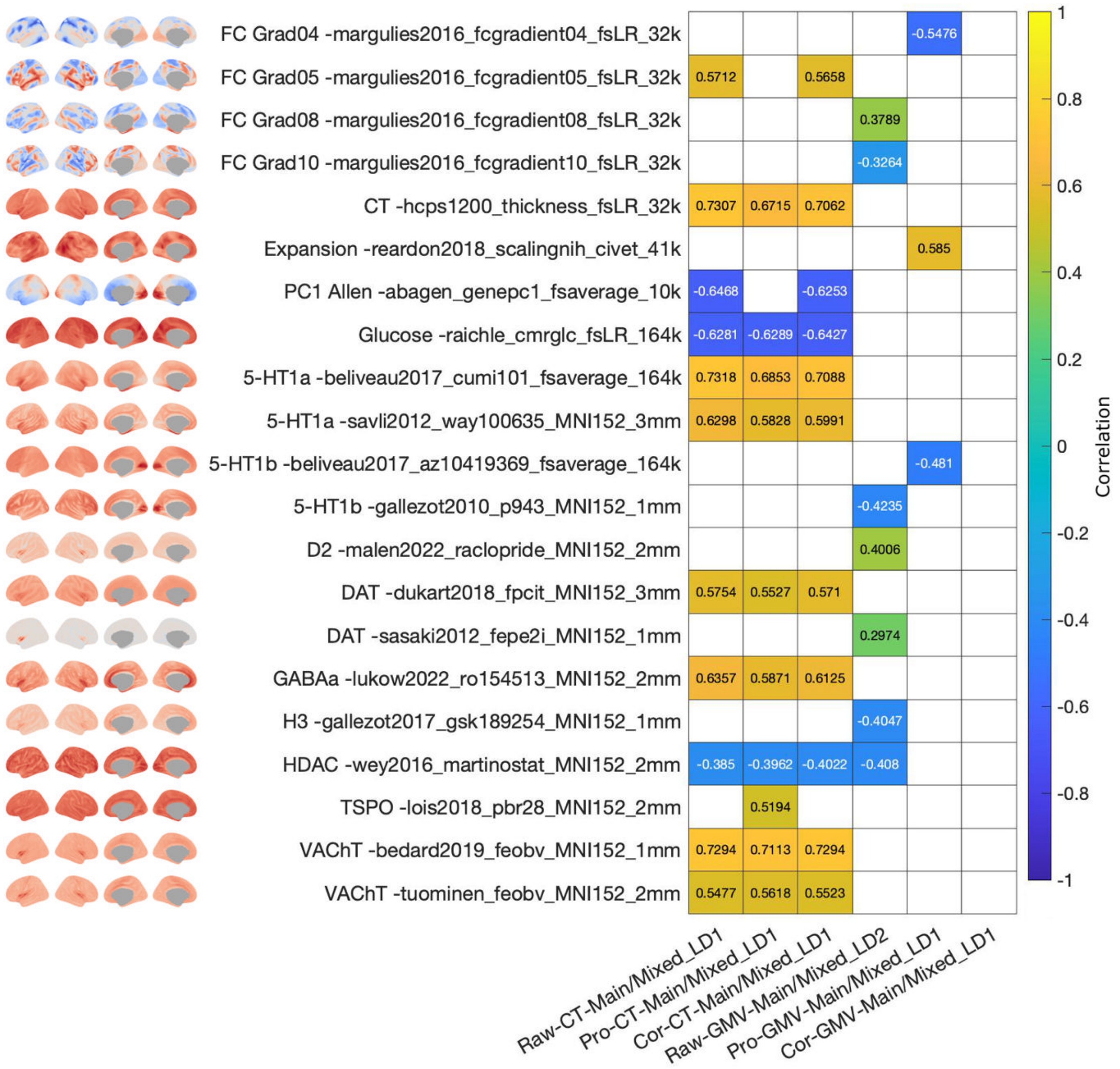

Figure AO. Association of brain structural loadings with brain maps for the latent dimension of cardiometabolic health. The association between the brain pattern of the latent dimension and brain maps was assessed with spin test. Only data for brain maps that yielded a significant association with at least one map of loadings are shown. White tiles represent non-significant associations. The data needed to generate this figure can be found in S5_Data.

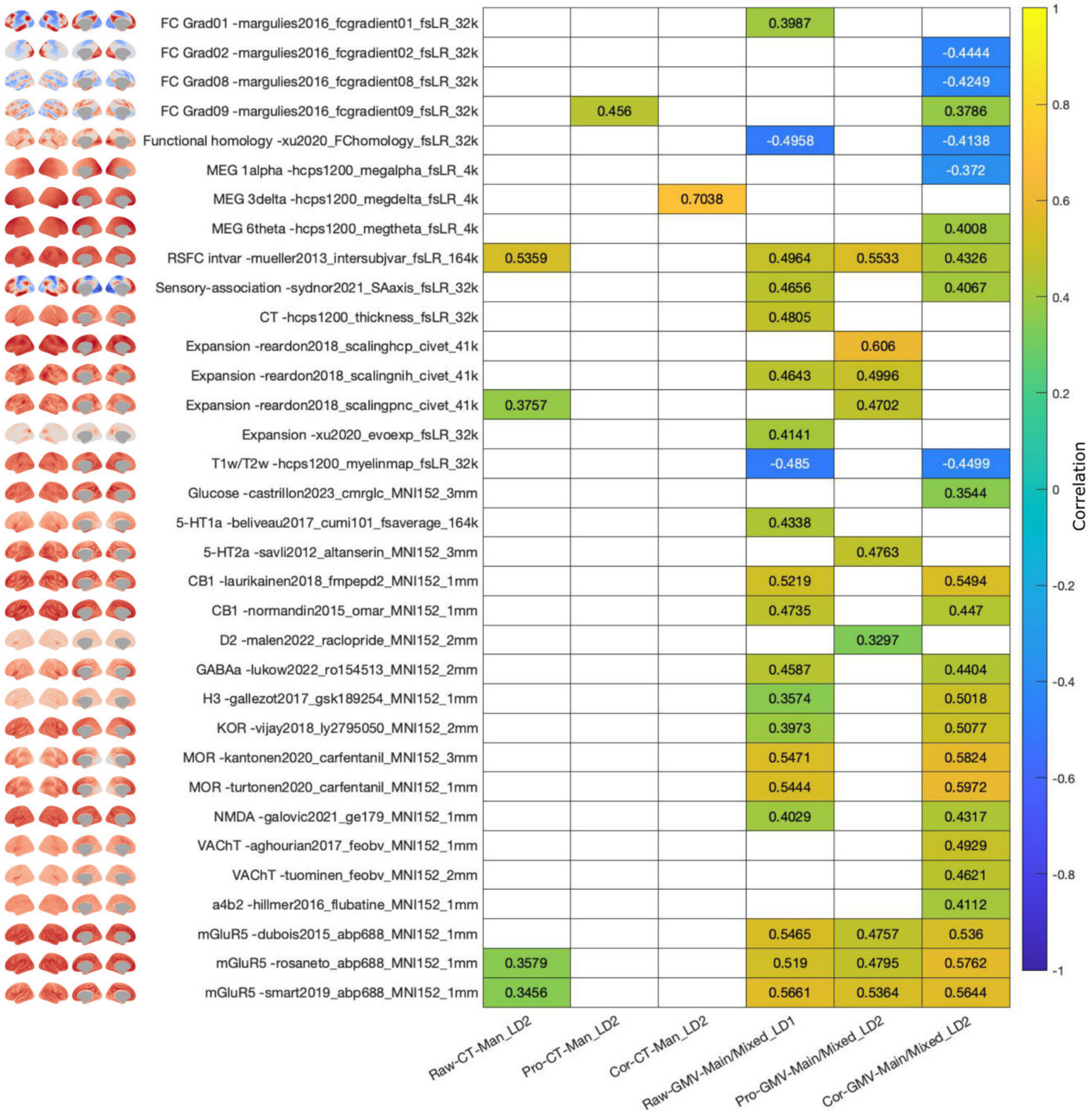

Figure AP. Association of brain structural loadings with brain maps for the latent dimension of physical robustness. The association between the brain pattern of the latent dimension and brain maps was assessed with spin test. Only data for brain maps that yielded a significant association with at least one map of loadings are shown. White tiles represent non-significant associations. The data needed to generate this figure can be found in S6_Data.

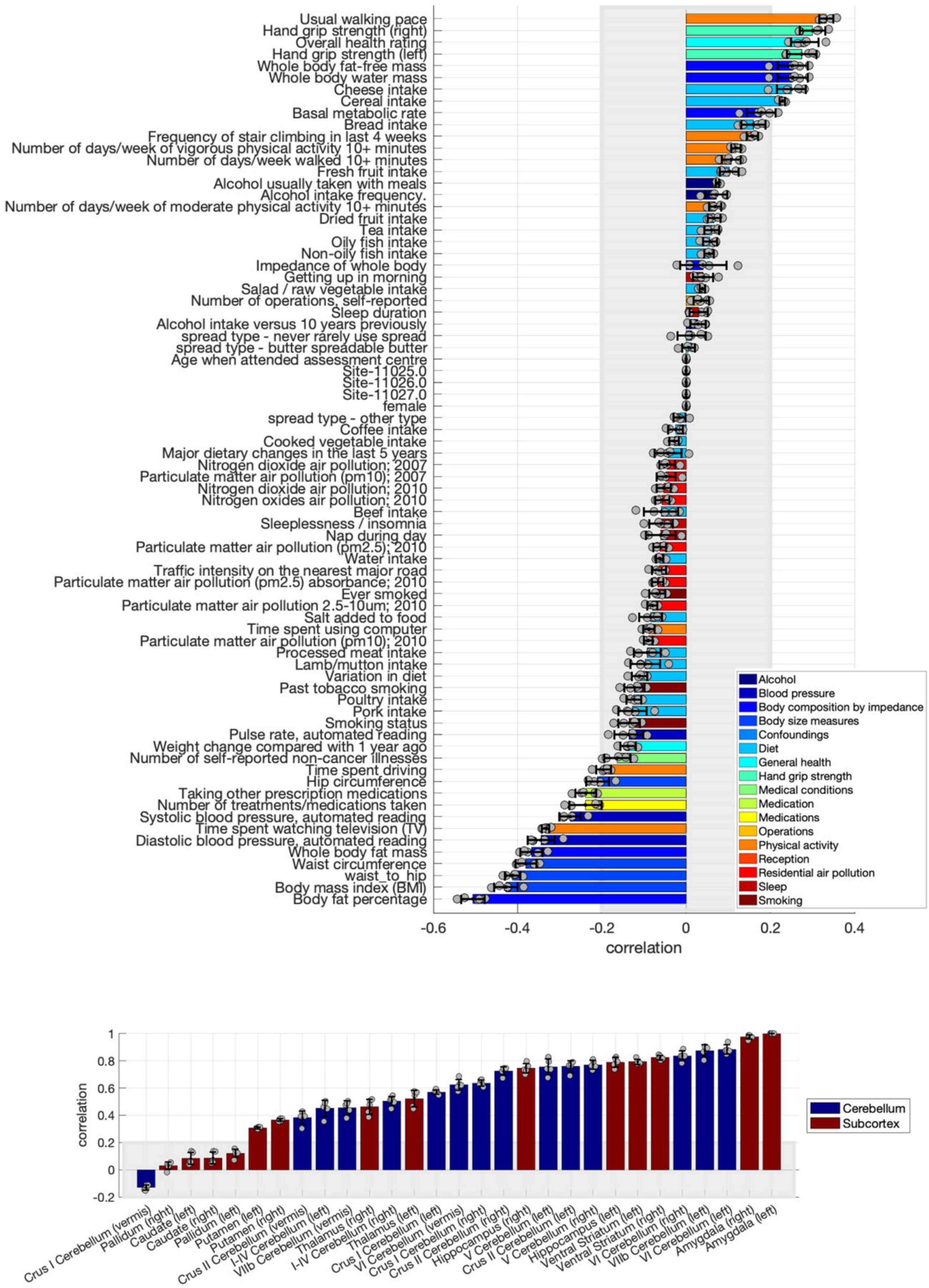

Figure AQ. Loadings of the first latent dimension linking risk factors to subcortical and cerebellar volumes. Risk factors loadings and brain loadings. Shown loadings represent the average over the five outer splits. Error bars depict one standard deviation. The shadowed zone marks loadings between −0.2 and 0.2. The data needed to generate this figure can be found in S69_Data.

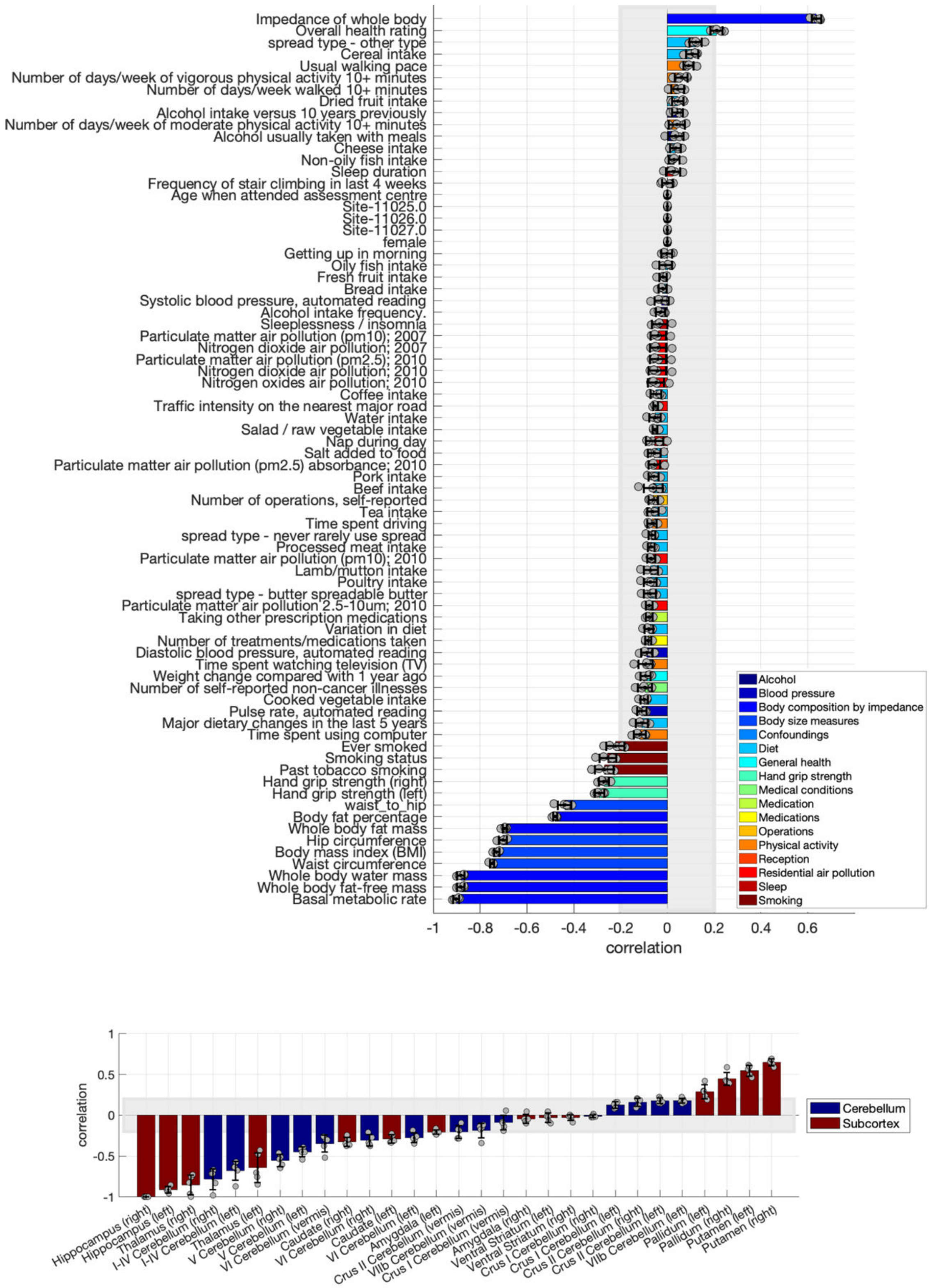

Figure AR. Loadings of the second latent dimension linking risk factors to subcortical and cerebellar volumes. Risk factors loadings and brain loadings. Shown loadings represent the average over the five outer splits. Error bars depict one standard deviation. The shadowed zone marks loadings between −0.2 and 0.2. The data needed to generate this figure can be found in S70_Data.

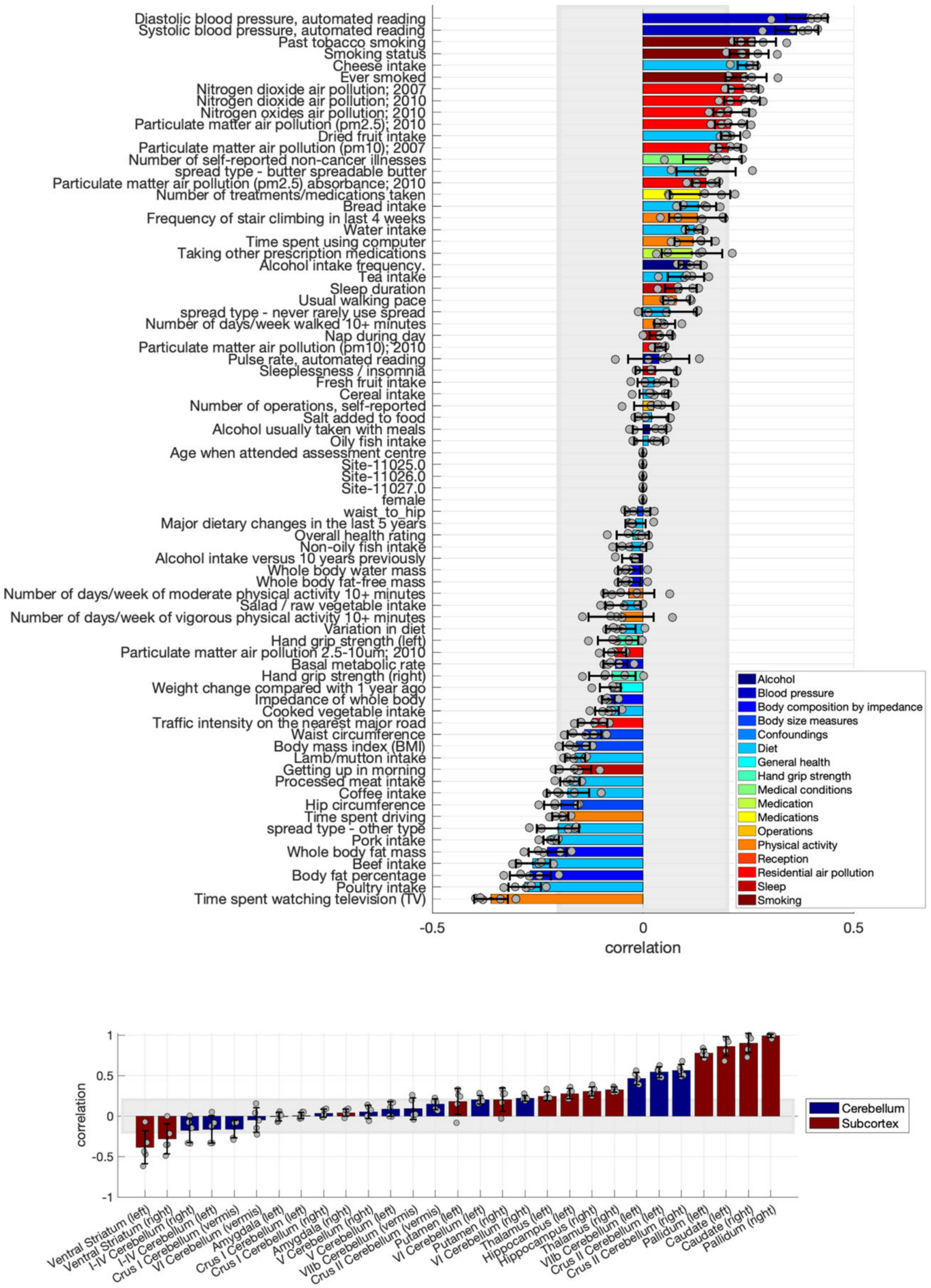

Figure AS. Loadings of the third latent dimension linking risk factors to subcortical and cerebellar volumes. Risk factors loadings and brain loadings. Shown loadings represent the average over the five outer splits. Error bars depict one standard deviation. The shadowed zone marks loadings between −0.2 and 0.2. The data needed to generate this figure can be found in S71_Data.

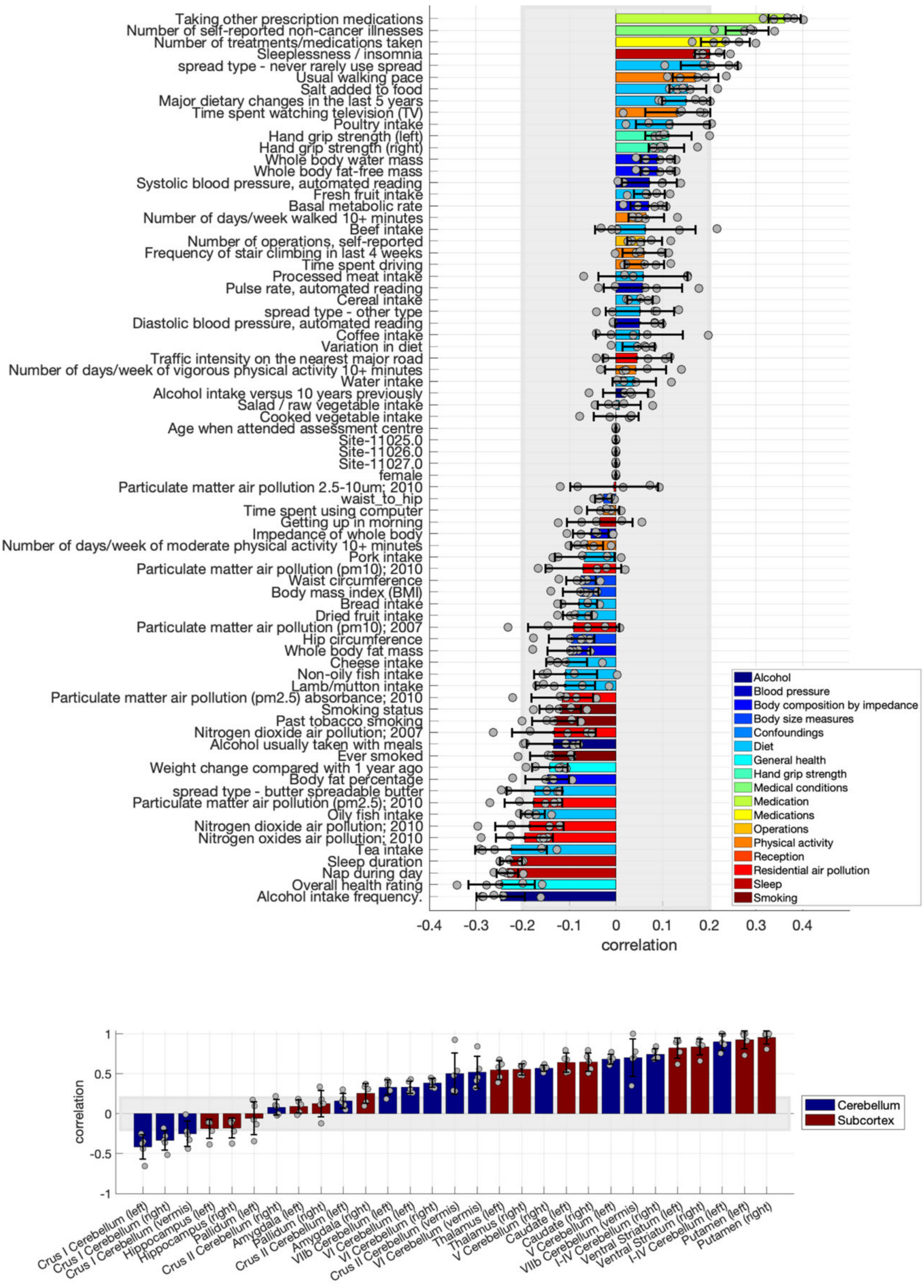

Figure AT. Loadings of the fourth latent dimension linking risk factors to subcortical and cerebellar volumes. Risk factors loadings and brain loadings. Shown loadings represent the average over the five outer splits. Error bars depict one standard deviation. The shadowed zone marks loadings between −0.2 and 0.2. The data needed to generate this figure can be found in S72_Data.

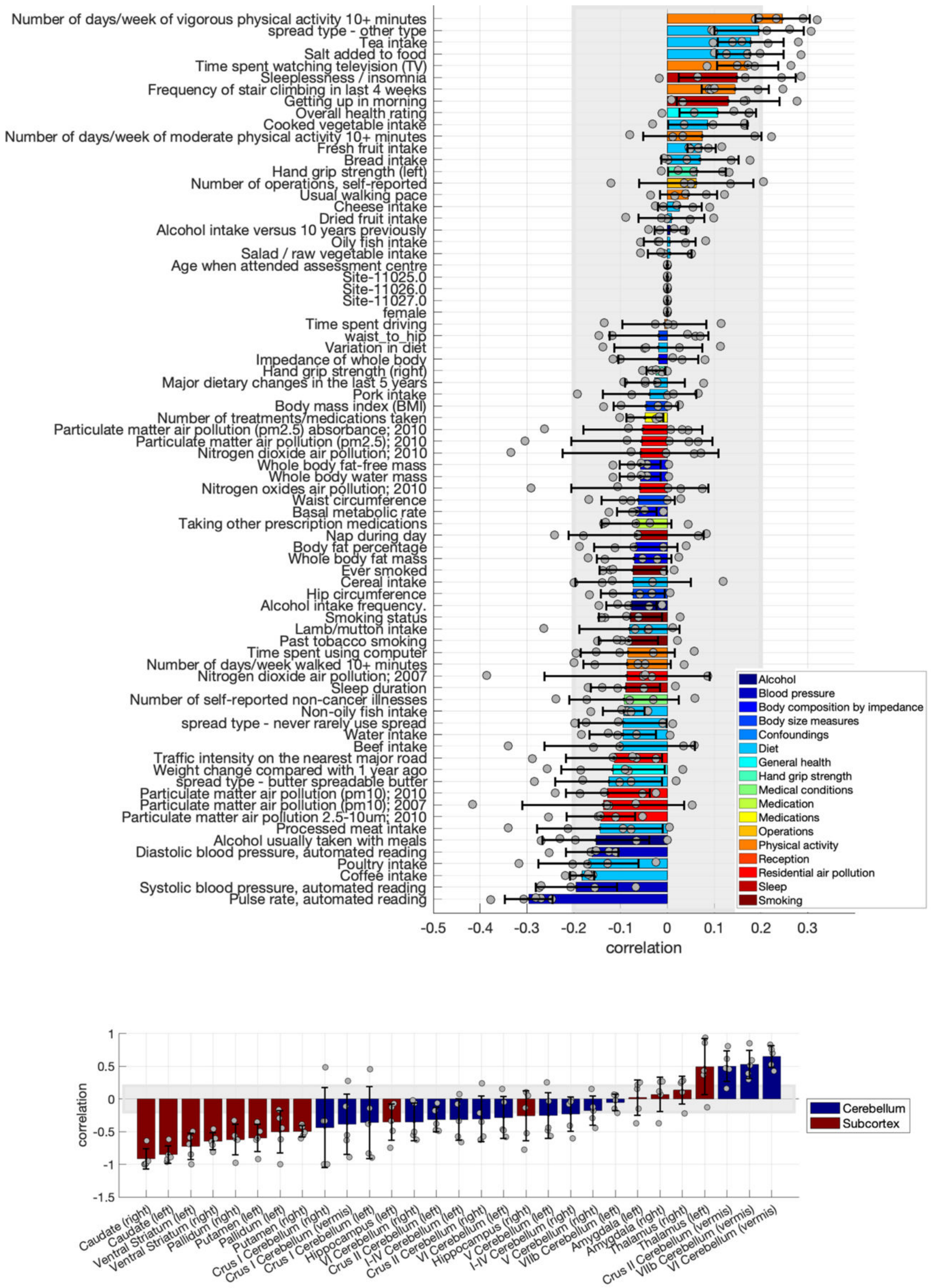

Figure AU. Loadings of the fifth latent dimension linking risk factors to subcortical and cerebellar volumes. Risk factors loadings and brain loadings. Shown loadings represent the average over the five outer splits. Error bars depict one standard deviation. The shadowed zone marks loadings between −0.2 and 0.2. The data needed to generate this figure can be found in S73_Data.

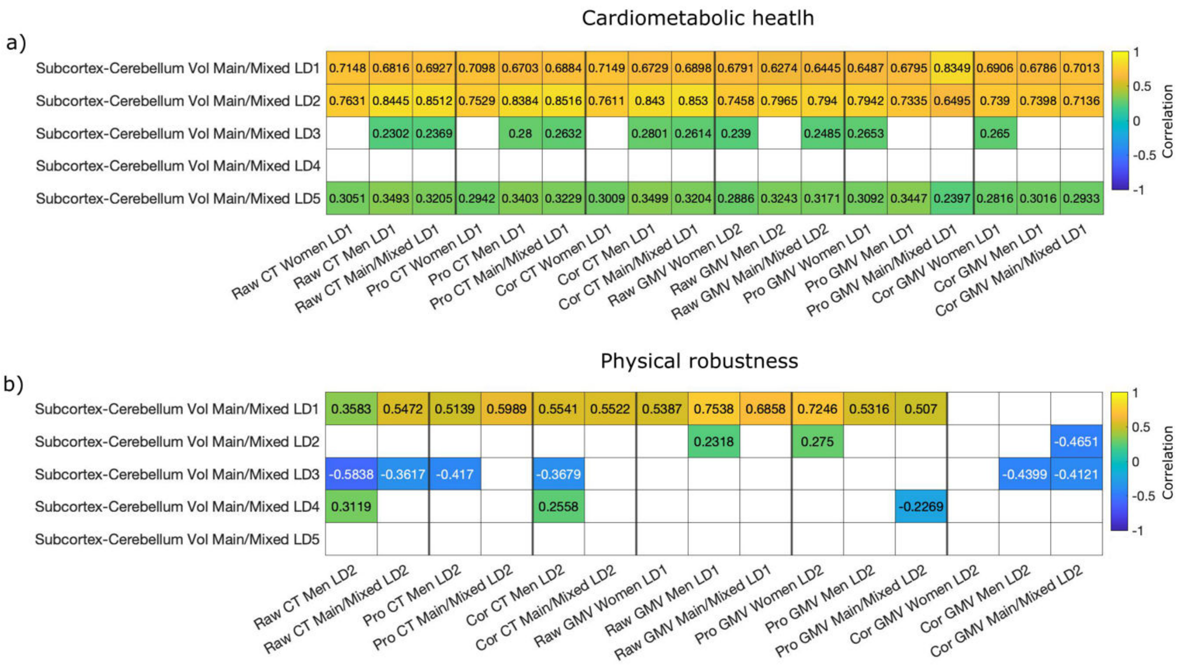

Figure AV. Comparison of risk factor loadings between latent dimensions yielded with subcortex-cerebellum and with CT and GMV in cortex. Panel a shows associations of risk factors for the latent dimension of cardiometabolic health. Panel b shows associations of risk factors for the latent dimension of physical robustness. Only associations that remain significant after Bonferroni correction are shown. White tiles represent non-significant associations. The data needed to generate this figure can be found in S74_Data (panel a) and S75_Data (panel b).

5. Bibliography

1. Kim SE, Lee JS, Woo S, Kim S, Kim HJ, Park S, et al. Sex-specific relationship of cardiometabolic syndrome with lower cortical thickness. Neurology. 2019;93(11):e1045–57.
2. Miller AA, Spencer SJ. Obesity and neuroinflammation: A pathway to cognitive impairment. Brain, Behavior, and Immunity. 2014 Nov;42:10–21.
3. Dresser R. Wanted single, white male for medical research. The Hastings Center Report. 1992;22(1):24–9.
4. Hodes GE, Kropp DR. Sex as a biological variable in stress and mood disorder research. Nature Mental Health. 2023;1(7):453–61.
5. Beery AK, Zucker I. Sex bias in neuroscience and biomedical research. Neuroscience & Biobehavioral Reviews. 2011;35(3):565–72.
6. Alfaro-Almagro F, Jenkinson M, Bangerter NK, Andersson JLR, Sotiropoulos SN, Jbabdi S, et al. Image processing and Quality Control for the first 10,000 brain imaging datasets from UK Biobank. NeuroImage. 2018;166(October 2017):400–24.
7. Sasse L, Nicolaisen-Sobesky E, Dukart J, Eickhoff SB, Götz M, Hamdan S, et al. Overview of leakage scenarios in supervised machine learning. J Big Data. 2025 May 29;12(1):135.
