# Supplementary material for: Cardiometabolic health and physical robustness map onto distinct patterns of brain structure and neurotransmitter systems
Source: PLoS Biol. 2025 Nov 21;23(11):e3003498. doi: 10.1371/journal.pbio.3003498 (PMC12671889; doi:10.1371/journal.pbio.3003498)
Supplement: Attachment — Submitted filename: ReviewersComments_v11.docx [file pbio.3003498.s005.docx]

We thank the Editor and the Reviewers for their valuable comments which have importantly helped us to improve our manuscript. Following the reviewers’ suggestions, in this revised version, we have considerably extended our results by adding analyses linking the same set of risk factors to grey matter volume (GMV). In addition, we have included supplementary analyses linking the same set of risk factors with subcortical and cerebellar volumes. We have also investigated associations of the risk factors scores with a peripheral marker of inflammation: C-reactive protein (CRP), strengthening the multi-level and systemics-view of our approach. Furthermore, we have provided additional details on the methods for clarification as requested by our reviewers. Finally, in addition to these new analyses, results and methodological details, we have further integrated our results with previous literature and provided a more holistic interpretation of our results.

In this response letter, the comments from the Reviewers appears in **black bold** font.

Our responses appear in *black italic* font.

Sections from our manuscript appear in *blue italic* font.

Considering the changes in the manuscript, we have changed the title to:

Risk factors, brain structure, and neurotransmitter systems: a large-scale multivariate study

**Reviewer #1:**

**The research presented uses regularized canonical correlation analysis (RRCA) to investigate relationships between factors related to cardiometabolic health and cortical thickness (CT). The weightings for significant imaging latent dimensions were then compared to atlases relating to neurotransmitters, brain metabolism, genetics and brain structure and function. Analyses were repeated for males, females, and all participants using raw, proportional, and corrected CT. The authors concentrate on the first latent dimension, which they link to "cardiometabolic health". This factor has the same sign of loadings for body fat, body water, and fat-free mass. Interestingly, the second latent dimension (Figure S9) shows opposite signs for fat-free and water mass compared to body fat percentage, although this dimension seems to be driven more by men (Figure S20) than by women (Figure S19). The authors state that "the second and third latent dimensions were also significant. However, the results point towards less cross-sex shared variability for the second and third latent dimensions given that they were unstable in the sex specific subsamples". It seems that in analyzing men and women separately, there is no guarantee of correspondence between the same latent dimension in the two groups.**

**Major comments**

**R1#1. It appears that men show a much more robust second latent dimension (Figure S20) than women (Figure S19). Men also show a fairly robust third latent dimension based on residential air pollution and smoking (Figure S21), although I don't see the corresponding data for women. There is an existing literature suggesting differential effects of air pollution on males and females which may or may not support the findings reported. Rather than dismissing this cross-sex variability, I would encourage the authors to embrace these interesting findings. This could be done by either calculating the loadings based on both groups, and then reporting the corresponding correlations for men and women, or by reporting and discussing separate RCCA of each group.**

*We appreciate the reviewer’s observation about the cross-sex variability in some of our results. We have now highlighted the cross-sex stability or variability of the reported latent dimensions across the manuscript. Please, note that according to other comments from reviewers we have added several analyses based on additional data. In particular, we have added analyses linking the set of risk factors to grey matter volume (GMV). We have addressed the cross-sex stability or variability within these additional analyses as well.*

*Following the reviewer’s comment, we have moved the second latent dimension to the main paper. This latent dimension appears stable across sexes when examining GMV, but stable or significant only in men when focusing on cortical thickness (CT). This is explained in lines 335-360:*

## *Latent dimension linking physical robustness to brain structure*

*Our results showed another significant latent dimension linking risk factors to raw CT (r_range_ = 0.09-0.12, p_range_ = 0.005-0.005) (Figure 3, Figures S25-S26, Table S4). Importantly, when studying raw CT, this latent dimension was significant and stable in the sample of men but not in the sample of women (see Supplementary results 2.5). For this reason, in the next steps we focus on the sample of men when considering this latent dimension with respect to CT. Also, note that this latent dimension corresponded to the second yielded latent dimension for all models except for the models for raw GMV, in which it was yielded as first latent dimension. This latent dimension captured variability in physical robustness (as opposed to physical frailty), with a positive pole associated to for instance whole body fat-free mass, grip strength and overall health rating, and a negative pole associated with smoking and air pollution.*

***Figure 3. Loadings of the latent dimension of physical robustness.*** *Risk factor loadings and brain loadings in the men sample for cortical thickness (left) and the main/mixed sample for and grey matter volume (right). The arrows depict the Spearman correlation of the loadings across models. The loadings represent the average over the five outer splits. Error bars depict one standard deviation. The shadowed zone marks loadings between −0.2 and 0.2.*

*This latent dimension of physical robustness was associated with a specific brain pattern of CT and a specific brain pattern of GMV (Figure 3), though the statistical comparison showed a marginally significant Spearman correlation indicating that they do share variance (r = 0.41, p = 0.046). However, this significant comparison was yielded only for the case of raw CT and raw GMV, but not for corrected brain structural measures (Figure S31-f). Conceptually, our results indicated that higher physical robustness was associated with higher CT, especially in superior frontal areas and insula. Moreover, the physical robustness pattern was associated to higher GMV, especially in the anterior cingulate cortex, medial superior frontal areas, orbitofrontal cortex, and temporal lobe.*

*We also moved the neurobiological characterization of such latent dimension to the main manuscript, in lines 399-422:*

## *Neurobiological characterization of the pattern of brain structural loadings associated with physical robustness*

*The brain patterns yielded for the latent dimension of physical robustness also showed several significant associations with brain maps (Table S11, Figure 5, Figure S42). The CT pattern yielded in the sample of men was positively associated with glutamate receptor mGluR5 (34,69), RSFC intersubject variability (70), and developmental area scaling (71). The GMV pattern was positively associated with the serotonin receptors 5-HT1a (54), with glutamate receptors mGluR5 (34,69,72) and NMDA (34,73–75), with histamine receptor H3 (68), with opioid receptors MOR (76,77) and KOR (78), with cannabinoid receptor CB1 (79,80), and with receptor GABAa (57). In addition, the GMV pattern was positively associated with the 1^st^ gradient of RSFC (60), intersubject variability of RSFC (70), cortical thickness (59), developmental area scaling (71), evolutionary expansion (81) and sensory-association axis (82). Moreover, the GMV pattern was negatively associated with T1w/T2w (59) and with functional homology (81).*

***Figure 5. Association of brain structural loadings with neuromaps for the latent dimension of physical robustness.*** *Only data for neuromaps that yielded a significant association with at least one of the shown loadings maps are depicted. Colorbar represents the Spearman correlation. White tiles represent non-significant associations.*

*Conceptually, this indicates that the latent dimension captured a brain axis in which cortical regions whose CT increased the most with the pattern of physical robustness (red regions in Figure 3) were those with highest binding potential for mGluR5. In addition, regions whose GMV increased the most with physical robustness (red regions) were those with highest binding potential for 5-HT1a, mGluR5, NMDA, H3, MOR, KOR, CB1 and GABAa.*

*Based on the overall profile of this latent dimension (capturing better variability in brain structure in men than in women and having most important loadings for muscles-mass/strength variables)), we refer to this latent dimension as “physical robustness” across the manuscript.*

*In order to clearly communicate in which results this latent dimension was unstable and not significant in women, details are provided in the Supplementary results in sections 2.5-2.7. For instance, in lines 182-185 regarding raw CT (Supplementary materials section 2.5):*

*However, in the sample of women it was significant in only one split (r = 0.103, p = 0.01) out of five (r_range_ = 0.013-0.027, p_range_ = 0.384-0.999). Considering that, and that the loadings were unstable for most of the risk factors loadings and brain regions in the sample of women (Figure S28), for the latent dimension of physical robustness and cortical thickness we focus on the sample of men.*

*And in lines 193-195 regarding proportional CT (Supplementary materials):*

*However, in the sample of women it was not significant (r_range_ = 0.003-0.060, p_range_ = 0.220-0.999). For this reason, for the latent dimension of physical robustness and cortical thickness we focus on the sample of men.*

*And in lines 205-208 regarding corrected CT (Supplementary materials):*

*However, in the sample of women it was significant in only one split (r = 0.108, p = 0.01) out of five (r_range_ = 0.003-0.047, p_range_ = 0.469-0.999). Considering that, and that the loadings were quite unstable (Figure S37), for the latent dimension of physical robustness and cortical thickness we focus on the sample of men.*

*Finally, we include a paragraph in the discussion addressing this point as well as the potential role of air pollution on the findings, in lines 502-529:*

*Our results also show that interindividual variability in physical robustness (fat-free mass and strength) is associated with interindividual variability in brain structure, in both men and women for GMV, but only robustly in men for CT. At the brain level, this CT pattern in men shows highest loadings mainly across the whole lateral frontal cortex and the superior medial frontal gyrus. The pattern of GMV associated to physical robustness also shows high loadings on medial frontal regions, along with lateral temporal regions. Accordingly, this GMV pattern is also significantly related to cortical patterns of developmental area scaling, along with evolutionary expansion and sensory-association axis. This may suggest common mechanisms underlying physical health (likely musculoskeletal health) and brain health in key regions for human high-level cognition (such as the frontal cortex). Interestingly, time spent watching television appears on the negative pole of these dimensions suggesting that sedentary lifestyles may threaten this aspect of human brain health.*

*It should be noted that the variability of physical robustness in women appears to be linked to GMV but not to CT, while in men it is associated to both structural markers. This sex-variability could be explained by the risk factors profile in association with CT in men capturing a negative pole of air pollution and smoking. Previous studies have pointed towards sex-variability in the effect of air pollution on health and particularly on brain and behavior (95–99). This could be due to sex-specific exposures to pollutants, with more interindividual variability in exposures in men allowing us to observe associations with health outcomes. In line with this hypothesis, the distribution of variables related to air pollution in our sample of men include a few more extreme values than in women (Figure S2). Alternatively, a potential sex-specific effect of air pollutants could also be due to different biological aspects being differentially affected in men in comparison to women, or to sex-specific pathways underpinning the association between air pollution and brain structure. The mechanisms underpinning this difference should be explored in future studies. Moreover, studies analyzing the association between health variables and neuroimaging should consider this and perform sex-specific analyses. In the current stage of knowledge, we can only speculate from our results that air pollution and smoking may represent a specific threat for men brain health in high-level associations regions.*

*Regarding the third latent dimension, even though it is significant, the effect sizes of the canonical correlation are very small (r = -0.001-0.15). For this reason, we do not focus on this latent dimension in this manuscript.*

**Minor comments**

**R1#2. Loadings Figures. Given that there only 68 risk factors available, it would seem more consistent to include all loadings in each diagram, even if they are not "stable". The brain loadings are important to the interpretation of the results. It may be better to include only medial and lateral views, but to make them larger.**

*We agree with the reviewer on the raised point. We have modified all figures showing loadings to a) show stable and unstable risk factor loadings and b) to show brain loadings in medial and lateral views increasing the size. These include Figures 2-5 in the main paper as well as Figures S9-S24, S28-S30, S32-40 and S43-S47.*

*Figures 2-3 are copied below:*

***Figure 2. Loadings of the latent dimension of cardiometabolic health.*** *Risk factor loadings and brain loadings in the main/mixed sample for cortical thickness (left) and grey matter volume (right). The arrows depict the Spearman correlation of the loadings across models. The loadings represent the average over the five outer splits. Error bars depict one standard deviation. The shadowed zone marks loadings between −0.2 and 0.2.*

***Figure 3. Loadings of the latent dimension of physical robustness.*** *Risk factor loadings and brain loadings in the men sample for cortical thickness (left) and the main/mixed sample for and grey matter volume (right). The arrows depict the Spearman correlation of the loadings across models. The loadings represent the average over the five outer splits. Error bars depict one standard deviation. The shadowed zone marks loadings between −0.2 and 0.2.*

**R1#3. It would be useful to be able to visually assess the associations between cortical thickness loadings and neuromaps (Figure 3), maybe with representative brain images side-by-side.**

*We thank the reviewer, the point raised will convey more information for the readers. We have added representative brain images of neuromaps in Figures 4-5 as well as Figures S41-S42. In addition, we have replace the previous barplots by heatmaps, which can summarize more information in a more easily readable fashion.*

***Figure 4. Association of brain structural loadings with neuromaps for the latent dimension of cardiometabolic health****. Only data for neuromaps that yielded a significant association with at least one of the shown loadings maps are depicted. Colorbar represents the Spearman correlation. White tiles represent non-significant associations.*

***Figure 5. Association of brain structural loadings with neuromaps for the latent dimension of physical robustness.*** *Only data for neuromaps that yielded a significant association with at least one of the shown loadings maps are depicted. Colorbar represents the Spearman correlation. White tiles represent non-significant associations.*

### ***Figure S41. Association of brain structural loadings with brain maps for the latent dimension of cardiometabolic health.*** *The association between the brain pattern of the latent dimension and brain maps was assessed with spin test. Only data for brain maps that yielded a significant association with at least one map of loadings are shown.* *White tiles represent non-significant associations.*

### ***Figure S42. Association of brain structural loadings with brain maps for the latent dimension of physical robustness.*** *The association between the brain pattern of the latent dimension and brain maps was assessed with spin test. Only data for brain maps that yielded a significant association with at least one map of loadings are shown. White tiles represent non-significant associations.*

**R1#4. The p-values reported in the main paper and the supplementary materials appear to be quantized in steps of 0.005, e.g. "rrange=0.30-0.34, p=0.005-0.005", Tables S4-S12.**

*We thank the reviewer for the careful examination of our work. It is correct that the p-values are quantized. This is because they are yielded via permutation test. In this case, the number of permutations used affects how small or big are the “steps” between the valid p-values that can be obtained, causing the observed quantization:*

$$p = \#(nulls > observed) / (n\_permutations +1)$$

*Where* $n\#(nulls > observed)$ *represent the number of permutations that yield a null association that is higher than the observed one.*

**R1#5. The ordering of risk factors in Figures S3 and S4 could be changed to better reflect the domains so that, for example, BMI and waist circumference are close together.**

*We agree with the reviewer that the suggested modification will improve the reading of such figures. Accordingly, we have ordered the risk factors per category and indicated them in the figure using the same color labels as in the figures showing the risk factor loadings (such as Figure 2):*

### ***Figure S3. Correlation matrix of risk factors in women.*** *Intercorrelation among risk factors in the subsample of women. Color bar depicts the Pearson’s correlation coefficient. Only significant associations after correction for multiple comparisons (Bonferroni) are shown. The colored bands indicate the categories to which the risk factors belong.*

***Figure S4. Correlation matrix of risk factors in men.*** *Intercorrelation among risk factors in the subsample of men. Color bar depicts the Pearson’s correlation coefficient. Only significant associations after correction for multiple comparisons (Bonferroni) are shown. The colored bands indicate the categories to which the risk factors belong.*

**R1#6. The authors suggest in the abstract that "regular monitoring of cardiometabolic risk factors should be considered in healthcare practice". While I don't disagree with this statement, it is already clinically well established that risk factors such as smoking, obesity, and hypertension cause neurovascular disease (often visible on routine MRI) that will almost certainly impact brain health (including stroke risk), and presumably cortical thickness.**

*We thank the reviewer for the valuable comment. We have removed that sentence and rephrased in the following way in the Summary (lines 73-76):*

*In this perspective, future studies should further examine the effects of risk/protective factors on different brain regions in order to deepen our understanding of the clinical significance of such increased and decreased CT and GMV.*

**R1#7. Table 1. Given a consistent number of regions, I would expect a monotonic relationship between the correlation coefficient and p-value. The numbers in this table look odd, for example row 16 has r=0.55, p=0.26 but row 19 has r=0.55, p<0.001. Again, there appears some quantization of p-vales (e.g. p=0.26 appears four times in the first column).**

*We thank the reviewer for raising this careful and valid point. Please, note that Table 1 is now Table S10 in supplementary materials. Accordingly, a similar table corresponding to the latent dimension that was added following suggestions from Reviewers and Editor appears as Table S11.*

*The results included in such tables (and all comparisons of brain patterns) are not derived from the “standard” Pearson correlation, but from the spin test. The standard Pearson correlation is not appropriate to study correspondance between brain patterns given that brain regions are not independent from each other (brain regions which are closer tend to have similar features) (Alexander-Bloch, et al., 2018 NeuroImage).*

*The spin test overcomes this limitation. In this non-parametric test, one of the brain patterns is projected to a sphere and randomly rotated a number of times (in our case, 1000 times). Then, the second brain map is correlated with each of the rotations of the first brain map, creating a null distribution. Finally, a p-value is computed as the number of iterations that yielded a stronger null result than the original result (original result here refers to the Pearson correlation of the original two maps) divided by the number of permutations plus 1. Hence, in this non-parametric test the p-value does not depend on the strength of the association as in the standard computation of the p-values for the Pearson correlation.*

*Alexander-Bloch, A. F., Shou, H., Liu, S., Satterthwaite, T. D., Glahn, D. C., Shinohara, R. T., ... & Raznahan, A. (2018). On testing for spatial correspondence between maps of human brain structure and function. Neuroimage, 178, 540-551. https://doi.org/10.1016/j.neuroimage.2018.05.070*

**Reviewer #2:**

**This study revealed a latent pattern of brain cortical thickness that associated with a range of cardiometabolic risk factors in a group of healthy individuals participating in the UK Biobank. They further showed that the latent cortical pattern is associated with the spatial distribution of several neurotransmitters. This is an interesting study, and the statistical analysis is robust.**

*We thank the reviewer for his/her positive evaluation of our manuscript.*

**Major comments**

**R2#1. *       My main concern is the lack of interpretation of the revealed CT pattern and their associated cardiometabolic risk factors. Although some interpretation is in place, it is insufficient to understand why increased and decreased CT differentially associated cardiometabolic risk factors across regions.**

*We thank the reviewer for his/her comment. Accordingly, we have now further worked on the interpretation of our results. After integrating additional analyses, we have careful examined the pattern of factors that appear in associations to brain structure across different analytic choices. This allows us to infer that one dimension reflects cardiometabolic health, while another dimension reflects physical robustness (as opposed to physical frailty). We have now renamed the latent dimension accordingly across the manuscript.*

*With respect to that, we have rephrased or added the following sections in the Discussion.*

*Lines 455-472, with respect to the latent dimension of cardiometabolic health:*

*We showed that the variability in cardiometabolic health is related to an axis of variability in CT extending from the insula and cingulate cortex to the occipital lobes and superior parietal regions. Overall, this latent dimension indicates that regions engaged in processing internal information such as emotional and motivational systems (including for instance insula, orbitofrontal cortex, and anterior cingulate cortex) show positive loadings. Accordingly, this CT pattern was significantly related to dopamine and serotonin systems. In contrast, more dorsal regions typically engaged in dorsal attention and executive systems (such as the lateral superior prefrontal and parietal cortex) show negative CT loadings on the latent dimension. Similarly, cardiometabolic health was negatively associated with GMV in the frontal regions. However, it was positively associated with GMV primarily in sensorimotor regions (peri-central regions), but also to a lesser extent to the ventral attention network (lateral parietal and middle temporal regions). This suggests that cardiometabolic health may be differently related to variability in CT and variability in GMV in the population. More concretely, our results suggest that better cardiometabolic health is related to increased CT in emotional and motivational systems along with increased GMV in sensorimotor cortex. Future studies should further elucidate the mechanism behind these associations to identify whether improving cardiometabolic health can promote brain structural health in motivation, emotion and sensorimotor networks and hence improve mental health and motor function in aging.*

*Lines 502-513, with respect to the latent dimension of physical robustness:*

*Our results also show that interindividual variability in physical robustness (fat-free mass and strength) is associated with interindividual variability in brain structure, in both men and women for GMV, but only robustly in men for CT. At the brain level, this CT pattern in men shows highest loadings mainly across the whole lateral frontal cortex and the superior medial frontal gyrus. The pattern of GMV associated to physical robustness also shows high loadings on medial frontal regions, along with lateral temporal regions. Accordingly, this GMV pattern is also significantly related to cortical patterns of developmental area scaling, along with evolutionary expansion and sensory-association axis. This may suggest common mechanisms underlying physical health (likely musculoskeletal health) and brain health in key regions for human high-level cognition (such as the frontal cortex). Interestingly, time spent watching television appears on the negative pole of these dimensions suggesting that sedentary lifestyles may threaten this aspect of human brain health.*

**R2#2. *       Another concern relates to the choice of the three different but related CT measures. The reproducible findings across the three measures are not surprising given that they are correlated. It would be helpful to elaborate the rationale of selecting the three measures and clarify the "different biological properties" mentioned in the manuscript.**

*We thank the reviewer for raising this point and we respectfully aknowledge the expressed perspective. However, some studies show that it is not always the case that correlated variables show similar associations with third variables. One example that supports this point can be illustrated by the new set of results that has been added according to other comments from reviewers. In particular, we have added analyses linking the set of risk factors to grey matter volume (GMV).*

*Regarding the point raised in the current comment, we would like to refer the latent dimension of physical robustness and GMV shown in Figure S31. In panels c and d it is possible to observe that some models did not yield significantly correlated latent dimensions when analysing different methods of brain size correction for grey matter volume (GMV). Moreover, in figures S41 and S42 it is possible to observe that even though certain maps of loadings are correlated, they show different associations with other brain maps:*

### ***Figure S41. Association of brain structural loadings with brain maps for the latent dimension of cardiometabolic health.*** *The association between the brain pattern of the latent dimension and brain maps was assessed with spin test. Only data for brain maps that yielded a significant association with at least one map of loadings are shown. White tiles represent non-significant associations.*

### ***Figure S42. Association of brain structural loadings with brain maps for the latent dimension of physical robustness.*** *The association between the brain pattern of the latent dimension and brain maps was assessed with spin test. Only data for brain maps that yielded a significant association with at least one map of loadings are shown.* White tiles represent non-significant associations.

*Regarding the expression “different biological properties”, what we intend to convey is that for instance, raw cortical thickness (CT) and corrected CT represent different markers of the individual brain phenotype.* *In particular, raw CT represents absolute CT, while corrected CT represents the relative regional value. Relative here is to be understood when taking into account the whole brain value. In other words, a corrected CT value for a given region expresses to which extend it has high or low CT when taking into account the global brain structure. Accordingly, corrected values are more likely to reveal structural patterns that pertain to specific brain regions. However, the drawback of correction or adjustment for head size is that it may discard relevant variance, i.e. variance that pertains to the effect we are interested in. For example, if variability in brain/head size is associated with variability in physical robustness for relevant biological reasons (eg: physically more robust individuals tend to be taller and more massive), adjusting for brain/head size can remove relevant variance related to physical robustness.*

*To clarify these points in the manuscript, we have added this explanation in lines 192-214:*

*We used mean raw CT and mean raw GMV of 148 cortical parcels (Destrieux atlas, a2009s) (45). To check the effect of brain size correction on the results, we also used proportional estimations of CT and GMV (computed subject-wise, dividing the mean raw CT or GMV of each parcel by the mean raw CT or Total Intracranial Volume (TIV), respectively, across all parcels) and CT and GMV corrected for brain size (regressing out mean CT or TIV, respectively, in a cross-validation consistent manner to avoid data leakage) (46). These three different brain structural measures (raw, proportional, or corrected) represent different biological properties and can show different patterns of associations with risk factors. For instance, raw CT represents the absolute CT of a brain region, while proportional CT can be understood as the ratio between a region’s CT and the whole brain’s CT. Along the same line, corrected CT can be seen as the regional value adjusted for the whole brain value, that is, the regional value which is not explained by the total brain value. Accordingly, proportional and corrected values are relative values, i.e. when taking into account the whole brain value. This means that a corrected CT value for a given region expresses to which extend it has high or low CT when taking into account the global brain structure. Accordingly, corrected values are more likely to reveal structural patterns that pertain to specific brain regions. However, the drawback of correction or adjustment for head/brain size is that it may discard relevant variance, i.e. variance that pertains to the effect we are interested in. For example, if variability in brain/head size is associated with variability in physical robustness for relevant biological reasons (e.g. physically more robust individuals tend to be taller and more massive), adjusting for brain/head size can remove relevant variance related to physical robustness. Therefore, row and uncorrected/unadjusted values have pros and cons and offer different insights in the current study.*

**R2#3. *       Similarly, the rationale of choosing CT over volume, surface area or other metrics are unclear. Gray matter volume is also an important marker of brain health.**

*We appreciate the opportunity to clarify this point. We focused on cortical thickness (CT) as our primary morphometric measure because it is considered a sensitive marker of microstructural cortical integrity and has been shown to capture aspects of neuronal loss, dendritic arborization, and myelination that are less influenced by inter-individual differences in head size (for instance, see Vidal-Pineiro, et al., 2020 Scientific reports; Winkler et al., 2010 Neuroimage, references at the end of the response to this comment). Regarding surface area, it is largely determined early in development and is relatively stable across adulthood, therefore that would not be the most relevant structural marker to investigate in associations to risk factors in an aging population.*

*However, we agree that grey matter volume (GMV) is also an important marker of brain health. To address this, we have now added analyses examining GMV alongside CT. The results of these analyses can be found in the Manuscript Results section and specifically in Figures 3 and 5 (see below). These analyses mainly showed a latent dimension of cardiometabolic risk factors that explained interindividual variability in GMV, similar to the first latent dimension that we found in association to CT. They also replicate a latent dimension that seems to pertain to physical robustness and that was previously observed in association to CT in men. Thus, overall, looking at interindividual variability in GMV pointed to the same latent dimensions of risk factors that were previously found to relate to interindividual variability in CT.*

*Lines 305-360:*

## *Latent dimension linking cardiometabolic health to brain structure*

*A first significant latent dimension was found when analyzing the link between risk factors to raw CT in the main/mixed sample (merging women and men) (r_range_ = 0.30-0.34, p_range_ = 0.005-0.005) (Figure 2, Figures S5-S6, Table S4). Conceptually, this latent dimension captures variability in cardiometabolic health, for instance with positive loadings for higher physical activity and a negative pole for factors of metabolic conditions, such as body fat, BMI and waist-to-hip ratio, as well as blood pressure. Of note, when analyzing the link between the same set of risk factors and GMV in the main/mixed sample a latent dimension with a highly similar profile of risk factors loadings was yielded (Figure 2, Figures S5 and S7, Table S7; Figure S8 for comparison across latent dimensions). Note that this latent dimension corresponded to the first yielded latent dimension for all models except for the models for raw GMV, in which it was yielded as second latent dimension.*

***Figure 2. Loadings of the latent dimension of cardiometabolic health.*** *Risk factor loadings and brain loadings in the main/mixed sample for cortical thickness (left) and grey matter volume (right). The arrows depict the Spearman correlation of the loadings across models. The loadings represent the average over the five outer splits. Error bars depict one standard deviation. The shadowed zone marks loadings between −0.2 and 0.2.*

*The pattern of cardiometabolic health was linked to a specific brain pattern of CT and a specific brain pattern of GMV (Figure 2). The CT pattern shows that better cardiometabolic health is associated with higher CT in the insula, cingulate cortex, temporal lobe, inferior parietal, orbitofrontal, and primary motor cortex, and to lower CT in the primary somatosensory cortex, superior frontal areas, superior parietal areas, and occipital areas. In addition, better cardiometabolic health is associated to higher GMV in primary motor cortex and temporal cortex, and lower GMV in frontal, parietal, and occipital areas.*

*Also in lines 335-360:*

## *Latent dimension linking physical robustness to brain structure*

*Our results showed another significant latent dimension linking risk factors to raw CT (r_range_ = 0.09-0.12, p_range_ = 0.005-0.005) (Figure 3, Figures S25-S26, Table S4). Importantly, when studying raw CT, this latent dimension was significant and stable in the sample of men but not in the sample of women (see Supplementary results 2.5). For this reason, in the next steps we focus on the sample of men when considering this latent dimension with respect to CT. Also, note that this latent dimension corresponded to the second yielded latent dimension for all models except for the models for raw GMV, in which it was yielded as first latent dimension. This latent dimension captured variability in physical robustness (as opposed to physical frailty), with a positive pole associated to for instance whole body fat-free mass, grip strength and overall health rating, and a negative pole associated with smoking and air pollution.*

***Figure 3. Loadings of the latent dimension of physical robustness****. Risk factor loadings and brain loadings in the men sample for cortical thickness (left) and the main/mixed sample for and grey matter volume (right). The arrows depict the Spearman correlation of the loadings across models. The loadings represent the average over the five outer splits. Error bars depict one standard deviation. The shadowed zone marks loadings between −0.2 and 0.2.*

*This latent dimension of physical robustness was associated with a specific brain pattern of CT and a specific brain pattern of GMV (Figure 3), though the statistical comparison showed a marginally significant Spearman correlation indicating that they do share variance (r = 0.41, p = 0.046). However, this significant comparison was yielded only for the case of raw CT and raw GMV, but not for corrected brain structural measures (Figure S31-f). Conceptually, our results indicated that higher physical robustness was associated with higher CT, especially in superior frontal areas and insula. Moreover, the physical robustness pattern was associated to higher GMV, especially in the anterior cingulate cortex, medial superior frontal areas, orbitofrontal cortex, and temporal lobe.*

*References:*

*Vidal-Pineiro, D., Parker, N., Shin, J., French, L., Grydeland, H., Jackowski, A. P., ... & Alzheimer’s Disease Neuroimaging Initiative and the Australian Imaging Biomarkers and Lifestyle flagship study of ageing. (2020). Cellular correlates of cortical thinning throughout the lifespan. Scientific reports, 10(1), 21803.*

*Winkler, A. M., Kochunov, P., Blangero, J., Almasy, L., Zilles, K., Fox, P. T., ... & Glahn, D. C. (2010). Cortical thickness or grey matter volume? The importance of selecting the phenotype for imaging genetics studies. Neuroimage, 53(3), 1135-1146.*

**R2#4. *       The choice of focusing on the cerebral cortex and ignoring the subcortical structure is also unclear. Subcortical nuclei/regions have been widely implicated in brain disorders (e.g., hippocampus in dementia, striatum in psychosis etc) that often comorbid with cardiometabolic conditions. It would be important to consider the subcortex in my opinion.**

*We also appreciate the opportunity to clarify this point. In order to address this comment, we have added analyses linking risk factors to subcortical and cerebellar volumes. These can be found in Results subsection “Latent dimensions linking risk factors to subcortical and cerebellar volumes” as well as in Figures S43-S48 and Table S12 (see below). Briefly, these analyses yielded several significant latent dimensions. However, only the first one show similarity with the pattern of risk factors that were found in associations with cortical thickness and grey matter volume. In other words, this latent dimension that explains interindividual variability in non-cortical regions seems to combine both cardiometabolic health and physical robustness factors. It relates to variability across a wide range of non-cortical regions, such as the amygdala, the hippocampus, the thalamus and several cerebellar regions, but to a lesser extend in basal ganglia regions.*

*The results can be found in lines 433-440 of the main manuscript:*

## *Latent dimensions linking risk factors to subcortical and cerebellar volumes*

*Results yielded by the analysis of subcortical and cerebellar data are reported in Supplementary results section 2.9. Overall, more significant latent dimensions appeared and only one was partly related to the two main dimensions or risk factors found in association to cortical structure. This suggests that subcortical regions have different patterns of interindividual variability than cortical regions (likely higher interindividual variability and more vulnerability to exposome factors). Accordingly, their associations with risk factors should be the focus of specific studies. In the following discussion, we focus on cortical structure.*

*Lines 221-234 in Supplementary results 2.9:*

*Latent dimensions linking risk factors to subcortical and cerebellar volumes*

*The analysis of subcortical and cerebellar data yielded five significant latent dimensions (Figures S43-S47, Table S12). Of those, the risk factor loadings of the first subcortex-cerebellum latent dimension showed significant associations with the loadings of both, the latent dimension of cardiometabolic health and the latent dimension of physical robustness yielded with the cortical data (CT and GMV) (Figure S48). The second latent dimension yielded with subcortex-cerebellum also showed significant associations with the latent dimension of cardiometabolic health. In other words, this first latent dimension that explains interindividual variability in non-cortical regions seems to combine both cardiometabolic health and physical robustness factors. It relates to variability across a wide range of non-cortical regions, such as the amygdala, the hippocampus, the thalamus and several cerebellar regions, but to a lesser extend in basal ganglia regions. This suggests that the set of factors that explain variability in subcortical regions in an aging population may be importantly more complex than the set of factors that relate cortical interindividual variability. Accordingly, we would encourage future studies to further specifically investigate factors that may affect subcortical structures across the lifespan, instead of considering cortical and subcortical regions globally.*

*Also in Table S12 and in Figures S43-S48 in Supplementary results:*

*Table S12. Statistics of the latent dimensions for subcortical and cerebellar volumes.*

|  | Main/Mixed sample | | | | | | | | | |
| --- | --- | --- | --- | --- | --- | --- | --- | --- | --- | --- |
|  | 1^st^ LD | | 2^nd^ LD | | 3^rd^ LD | | 4^th^ LD | | 5^th^ LD | |
| Split | r | p-value | r | p-value | r | p-value | r | p-value | r | p-value |
| 1 | 0.27 | **0.005** | 0.13 | **0.005** | 0.08 | **0.005** | 0.03 | 0.459 | 0.03 | 0.739 |
| 2 | 0.25 | **0.005** | 0.14 | **0.005** | 0.10 | **0.005** | 0.08 | **0.005** | 0.02 | 0.889 |
| 3 | 0.27 | **0.005** | 0.18 | **0.005** | 0.11 | **0.005** | 0.08 | **0.005** | 0.03 | 0.424 |
| 4 | 0.27 | **0.005** | 0.17 | **0.005** | 0.08 | **0.025** | 0.06 | 0.115 | 0.003 | >0.99 |
| 5 | 0.26 | **0.005** | 0.16 | **0.005** | 0.11 | **0.005** | 0.05 | 0.105 | 0.06 | **0.03** |

*p-values are corrected using the Bonferroni method over 5 comparisons; significant p-values are in bold; r: Pearson’s correlation coefficient*

***Figure S43. Loadings of the first latent dimension linking risk factors to subcortical and cerebellar volumes.*** *Risk factors loadings and brain loadings. Shown loadings represent the average over the five outer splits. Error bars depict one standard deviation. The shadowed zone marks loadings between −0.2 and 0.2.*

***Figure S44. Loadings of the second latent dimension linking risk factors to subcortical and cerebellar volumes.*** *Risk factors loadings and brain loadings. Shown loadings represent the average over the five outer splits. Error bars depict one standard deviation. The shadowed zone marks loadings between −0.2 and 0.2.*

***Figure S45. Loadings of the third latent dimension linking risk factors to subcortical and cerebellar volumes.*** *Risk factors loadings and brain loadings. Shown loadings represent the average over the five outer splits. Error bars depict one standard deviation. The shadowed zone marks loadings between −0.2 and 0.2.*

***Figure S46. Loadings of the fourth latent dimension linking risk factors to subcortical and cerebellar volumes.*** *Risk factors loadings and brain loadings. Shown loadings represent the average over the five outer splits. Error bars depict one standard deviation. The shadowed zone marks loadings between −0.2 and 0.2.*

***Figure S47. Loadings of the fifth latent dimension linking risk factors to subcortical and cerebellar volumes.*** *Risk factors loadings and brain loadings. Shown loadings represent the average over the five outer splits. Error bars depict one standard deviation. The shadowed zone marks loadings between −0.2 and 0.2.*

***Figure S48. Comparison of risk factor loadings between latent dimensions yielded with subcortex-cerebellum and with CT and GMV in cortex.*** *Panel a shows associations of risk factors for the latent dimension of cardiometabolic health. Panel b shows associations of risk factors for the latent dimension of physical robustness. Only associations that remain significant after Bonferroni correction are shown.*

**Reviewer #3 Yunpeng Wang (note, Reviewer 3 has signed this review)**

**Nicolaisen-Sobesky et al. investigated the relationship between 68 cardiometabolic variables and brain cortical thickness using a carefully selected subset of the UK Biobank dataset. Unlike previous work, this study employed regularized canonical correlation analysis (RCCA), which accounts for dependencies within both sets of variables. This approach allows for a more holistic understanding of brain-body relationships. The authors identified several statistically significant associations, which appear robust and generalizable within UKBB. They further linked the latent dimensions to brain maps derived from external datasets. The novelty of this study includes: An important contribution to the literature on brain-body connections, particularly in the context of commonly studied physical health risk factors and MRI-derived brain features. The finding that some risk factors are associated with increased cortical thickness in certain regions and decreased thickness in others. This challenges the assumption—common in prior literature—that thicker cortex uniformly implies better function. Integration of latent dimensions with previously established brain maps.**

*We are grateful for your positive evaluation of our work and thoughtful comments on our manuscript.*

**However, several issues need to be addressed:**

**Major:**

**R3#1. Repeated measures: It is unclear which measurements of the risk factors were used. Did the authors use the measurement closest in time to the brain scan? Were other criteria used? This should be clearly described.**

*All the risk factor variables included in this study were acquired during the Imaging visit, except the variables associated to “Residential air pollution”, which were acquired before the Imaging visit. We have clarified this important point in lines 176-178:*

*All variables corresponding to risk factors were acquired on the Imaging visit, with the exception of those in the Category of “Residential air pollution” (Table S1), which were acquired before the Imaging visit.*

**R3#2. Overemphasis on inflammation in the Discussion: About 60% of the Discussion focuses on inflammation and chronic inflammation as potential mechanisms. While this is a plausible interpretation, the UKBB includes actual inflammatory biomarkers (e.g., CRP, OLINK inflammation panel). The authors should consider testing these directly or, at the very least, connect their current findings to previous results based on these measures. For example, are there risk factors that showed strong associations in univariate analyses but not here, or vice versa?**

*We appreciate the reviewer raising this important point. Accordingly, we have added analyses testing the association between the latent dimensions and C-reactive protein (CRP).* *We focused on CRP as the most relevant marker for chronic low-grade inflammation having a cardiometabolic link, good signal-to-noise ratio for population studies, relative stability in time, and low missingness in UK Biobank.*

*The analyses are described in lines 280-286:*

## *Association of the latent dimensions with peripheral inflammation*

*We explore the association between the composite variable of risk factors (i.e. the risk factor scores which are subject-wise values) and a peripheral marker of inflammation with Spearman correlation. We focused on C-reactive Protein (CRP, field-id 30710-1.0 in the UKB dataset) as the most relevant marker for chronic and systemic low-grade inflammation having a good signal-to-noise ratio for population studies, a relative stability in time, and a relatively low missingness in UK Biobank. Multiple comparisons were corrected with Bonferroni.*

*The findings are listed in lines 423-432:*

## *Correlation of the latent dimensions with peripheral marker of inflammation*

*CRP was significantly associated to the risk factors scores of cardiometabolic health when yielded in association with raw CT (r = -0.39, p < 0.001) and with raw GMV (r = -0.39, p < 0.001) in the main/mixed sample. It was also significantly associated to the risk factors pattern of physical robustness yielded in association with raw GMV in the main/mixed sample (r = -0.08, p = 0.04) but with a very low effect size. CRP was not significantly associated with the risk factors yielded in association with raw CT in the sample of men (r = 0.038, p = 0.999). Thus, overall, the composite variable of cardiometabolic health appears moderately correlated to a marker of low-grade systemic inflammation, while physical robustness was only very weakly associated ( r < 0.1) to such a marker.*

**R3#3. In sum, while the methodological approach and findings are interesting and potentially impactful, the manuscript would benefit from clearer methodological details and a stronger integration with prior work.**

*We thank the reviewer for his feedback. We have now added additional methodological details and we have also further interpreted our findings in line with prior work. For instance, we have clarified the interpretation of our results of associations with neuromaps in the figures (replacing the barplots by heatmaps, and adding comparative brainmaps side-by-side in Figures 4 and 5) as well as in the manuscript:*

*Lines 391-398:*

*Conceptually, this indicates that the latent dimension captured a brain axis in which cortical regions whose CT covaried positively the most (increased the most, red regions in Figure 2) with the pattern of cardiometabolic health were regions that showed the highest binding potentials for 5-HT1a, DAT, GABAa and VAChT. Similarly, the brain regions whose GMV increased the most (red regions) with cardiometabolic health were regions with the highest binding potential for D2 and DAT. In turn, the brain regions whose GMV covaried negatively the most (decreased the most, blue regions) with cardiometabolic health were regions with the highest binding potentials for 5-HT1b and H3.*

*Lines 418-422:*

*Conceptually, this indicates that the latent dimension captured a brain axis in which cortical regions whose CT increased the most with the pattern of physical robustness (red regions in Figure 3) were those with highest binding potential for mGluR5. In addition, regions whose GMV increased the most with physical robustness (red regions) were those with highest binding potential for 5-HT1a, mGluR5, NMDA, H3, MOR, KOR, CB1 and GABAa.*

### ***Figure 4. Association of brain structural loadings with neuromaps for the latent dimension of cardiometabolic health.*** *Only data for neuromaps that yielded a significant association with at least one of the shown loadings maps are depicted. Colorbar represents the Spearman correlation*. White tiles represent non-significant associations.

### ***Figure 5. Association of brain structural loadings with neuromaps for the latent dimension of physical robustness.*** *Only data for neuromaps that yielded a significant association with at least one of the shown loadings maps are depicted. Colorbar represents the Spearman correlation.* *White tiles represent non-significant associations.*

*In addition, we have strengthened the discussion of our results in the following sections in discusion:*

*Lines 455-472, with respect to the latent dimension of cardiometabolic health:*

*We showed that the variability in cardiometabolic health is related to an axis of variability in CT extending from the insula and cingulate cortex to the occipital lobes and superior parietal regions. Overall, this latent dimension indicates that regions engaged in processing internal information such as emotional and motivational systems (including for instance insula, orbitofrontal cortex, and anterior cingulate cortex) show positive loadings. Accordingly, this CT pattern was significantly related to dopamine and serotonin systems. In contrast, more dorsal regions typically engaged in dorsal attention and executive systems (such as the lateral superior prefrontal and parietal cortex) show negative CT loadings on the latent dimension. Similarly, cardiometabolic health was negatively associated with GMV in the frontal regions. However, it was positively associated with GMV primarily in sensorimotor regions (peri-central regions), but also to a lesser extent to the ventral attention network (lateral parietal and middle temporal regions). This suggests that cardiometabolic health may be differently related to variability in CT and variability in GMV in the population. More concretely, our results suggest that better cardiometabolic health is related to increased CT in emotional and motivational systems along with increased GMV in sensorimotor cortex. Future studies should further elucidate the mechanism behind these associations to identify whether improving cardiometabolic health can promote brain structural health in motivation, emotion and sensorimotor networks and hence improve mental health and motor function in aging.*

*Lines 502-529, with respect to the latent dimension of physical robustness:*

*Our results also show that interindividual variability in physical robustness (fat-free mass and strength) is associated with interindividual variability in brain structure, in both men and women for GMV, but only robustly in men for CT. At the brain level, this CT pattern in men shows highest loadings mainly across the whole lateral frontal cortex and the superior medial frontal gyrus. The pattern of GMV associated to physical robustness also shows high loadings on medial frontal regions, along with lateral temporal regions. Accordingly, this GMV pattern is also significantly related to cortical patterns of developmental area scaling, along with evolutionary expansion and sensory-association axis. This may suggest common mechanisms underlying physical health (likely musculoskeletal health) and brain health in key regions for human high-level cognition (such as the frontal cortex). Interestingly, time spent watching television appears on the negative pole of these dimensions suggesting that sedentary lifestyles may threaten this aspect of human brain health.*

*It should be noted that the variability of physical robustness in women appears to be linked to GMV but not to CT, while in men it is associated to both structural markers. This sex-variability could be explained by the risk factors profile in association with CT in men capturing a negative pole of air pollution and smoking. Previous studies have pointed towards sex-variability in the effect of air pollution on health and particularly on brain and behavior (95–99). This could be due to sex-specific exposures to pollutants, with more interindividual variability in exposures in men allowing us to observe associations with health outcomes. In line with this hypothesis, the distribution of variables related to air pollution in our sample of men include a few more extreme values than in women (Figure S2). Alternatively, a potential sex-specific effect of air pollutants could also be due to different biological aspects being differentially affected in men in comparison to women, or to sex-specific pathways underpinning the association between air pollution and brain structure. The mechanisms underpinning this difference should be explored in future studies. Moreover, studies analyzing the association between health variables and neuroimaging should consider this and perform sex-specific analyses. In the current stage of knowledge, we can only speculate from our results that air pollution and smoking may represent a specific threat for men brain health in high-level associations regions.*

**Minor:**

**Some references need attention. For instance:**

*We thank the reviewer for having noticed such issues in the references.*

**R3#4. References 22 and 27 appear to be duplicates.**

*References 22 and 27 were indeed duplicates. They now appear as reference 23.*

**R3#5. Reference 38 lacks complete journal information.**

*Journal information of reference 38 has been added (now reference 43).*

**R3#6. Reference 75 may not be the correct citation.**

*Reference 75 (now ref 90) is cited in our manuscript in lines 491-493:*

*In turn, low-grade systemic chronic inflammation has been associated with non-communicable diseases (6,88), including neuropsychiatric illnesses (21,38,39,88–92).*

A*nd in lines 496-498:*

*In fact, inflammation has been proposed as the cause of comorbidities between non-communicable diseases (86,90), such as depression and cardiovascular or neurodegenerative illnesses (90).*

*And in lines 556-559:*

*Moreover, since these neurotransmitter systems are implicated in mental disorders and in somatic non-communicable diseases (88,90), the mechanisms underlying the comorbidity between mental and somatic disorders might be partly related with alterations in neurotransmitter systems (86).*

*The cited article corresponds to an extensive review on the topic of the commorbidity between major depression and neurodegenerative disorders, with a special emphasis on the role of neurotransimtters and immune factors (cytokines) in such relationship. In addition, the review collects evidence about the relationship between depression and some somatic non-comunicable diseases, such as heart problems. Below, there are extractions from such review that show how the review supports the sentences written in our manuscript. Highlights were added for emphasis, and text inside [] was added for clarification of acronyms.*

*In section “1. Introduction”, the work of the article is presented as follows:*

*“The present report describes the contribution of stressful events to the emergence of various endophenotypes that comprise depression, including the possible biological processes (neurotransmitter, hormone, neuropeptide, growth factor and cytokine) that might be responsible for the disorder. In so doing, we will outline some of the moderating conditions that influence these biological changes, and will relate stress and depression to comorbid illnesses, including those associated with the aftermath of stroke (and head injury) and those of a neurodegenerative nature (Parkinson’s disease and Alzheimer’s disease), with the aim of defining some of the shared processes that subserve them.” (page 3).*

*Section “3. Biological correlates of depression” presents neurotransmitter alterations in depression. This section also presents the association between depression and neurodegenerative processes with the immune system:*

*“Moreover, depression has been associated with alterations of peptidergic functioning (e.g., corticotropin releasing hormone; CRH), including neuronal release or the number and sensitivity of peptide receptors (Mitchell, 1998; Nemeroff, 1996; Reul and Holsboer, 2002), as well as alterations of growth factors (e.g., brain-derived neurotrophic factor; BDNF) (Duman and Mon- teggia, 2006; Duman et al., 2000, 2001; Manji et al., 2001) and cytokines that are more typically considered as immune system signaling molecules (e.g., interleukin-1, -2 and -6, interferon-a, tumor necrosis factor-a (Hayley et al., 2005; Maes, 1999)).” (page 5).*

*“In this regard, a major focus of the model provided in Fig. 1 is that certain stressors, like immunogenic challenges, may provoke activation of the inflammatory immune system, and the ensuing release of immune signaling molecules (cytokines). These cytokines, in effect, function like stressors in affecting neurochemical processes, and may act synergistically with traditional stressors in provoking these outcomes)… It is believed that the sensitization of neurochemical responses might contribute to the development of pathology, such as depression. Moreover, because these same mechanisms also contribute to neurodegenerative processes, it is suggested that synergies between stressors and cytokines, and the sensitization of the neurochemical processes associated with stressors, may contribute to comorbidity frequently seen between psychological and neurodegenerative disorders.” (page 6).*

*In the article, there are sections describing the relationship between depression and different neurotransmitter systems, such as “5. Serotonin signaling and depression in stress-based models of depression” (page 9) and “10. Gamma-aminobutyric acid (GABA)” (page 21).*

*There is also a subsection that reviews evidence of how the immune markers can affect the central nervous system and in particular how they are linked to to depression (“11 Cytokines”, page 23) and to neurodegeneration (“14. Neuropeptide and cytokine influences on neurodegeneration: Contribution of the inflammatory immune system”, page 37; “15. Neuropeptide and cytokine interactions in the modulation of neurodegeneration”, page 39). In addition, a section collects evidence on how immune markers mediate the relationship between depression and other neurodegenerative conditions (“16. Cytokines mediate the comorbidity between depression and neurodegenerative conditions”, page 44) as well as somatic illnesses (“16.1 Depression and comorbid cardiovascular (heart and stroke) disturbances”, page 44).*

*Finally, in section “17. Summary and conclusion” it is mentioned:*

*“The neurochemical features associated with the MDD [Major depressive disorder] symptom profile span neurotransmitters, peptidergic factors, inflammatory processes, and factors that influence neuronal plasticity and neurogenesis. Some of these same features are also prominent in illnesses that are comorbid with MDD, most notably cardiovascular related events (including stroke and its aftermath), PD [Parkinson’s disease] and AD [Alzheimer’s disease]. In effect, it seems that inflammatory processes are a common denominator for these disorders, and it may well be that they contribute to the provocation of these different pathological states.”. (page 47).*

*For all of the above, we believe that this citation supports the sentences where it is cited in our manuscript.*

*Bibligoraphy:*

*34.	Hansen JY, Shafiei G, Markello RD, Smart K, Cox SML, Nørgaard M, et al. Mapping neurotransmitter systems to the structural and functional organization of the human neocortex. Nature Neuroscience. 2022;25(11):1569–81.*

*54.	Beliveau V, Ganz M, Feng L, Ozenne B, Højgaard L, Fisher PM, et al. A High-Resolution In Vivo Atlas of the Human Brain’s Serotonin System. 2017;37(1):120–8.*

*57.	Lukow PB, Martins D, Veronese M, Vernon AC, McGuire P, Turkheimer FE, et al. Cellular and molecular signatures of in vivo imaging measures of GABAergic neurotransmission in the human brain. Communications Biology. 2022;5(1):1–11.*

*59.	Glasser MF, Coalson TS, Robinson EC, Hacker CD, Harwell J, Yacoub E. A multi-modal parcellation of human cerebral cortex. Nature Publishing Group. 2016;536(7615):171–8.*

*60.	Margulies DS, Ghosh SS, Goulas A, Falkiewicz M, Huntenburg JM, Langs G, et al. Situating the default-mode network along a principal gradient of macroscale cortical organization. Proceedings of the National Academy of Sciences of the United States of America. 2016;113(44):12574–9.*

*68.	Gallezot JD, Planeta B, Nabulsi N, Palumbo D, Li X, Liu J, et al. Determination of receptor occupancy in the presence of mass dose: [^11^ C]GSK189254 PET imaging of histamine H_3_ receptor occupancy by PF-03654746. J Cereb Blood Flow Metab. 2017 Mar;37(3):1095–107.*

*69.	Smart K, Cox SML, Scala SG, Tippler M, Jaworska N, Boivin M, et al. Sex differences in [11C]ABP688 binding: a positron emission tomography study of mGlu5 receptors. Eur J Nucl Med Mol Imaging. 2019 May;46(5):1179–83.*

*70.	Mueller S, Wang D, Fox MD, Yeo BTT, Sepulcre J, Sabuncu MR, et al. Individual Variability in Functional Connectivity Architecture of the Human Brain. Neuron. 2013 Feb;77(3):586–95.*

*71.	Reardon PK, Seidlitz J, Vandekar S, Liu S, Patel R, Lalonde FM, et al. Normative brain size variation and brain shape diversity in humans. 2018;1227(June):4–8.*

*72.	DuBois JM, Rousset OG, Rowley J, Porras-Betancourt M, Reader AJ, Labbe A, et al. Characterization of age/sex and the regional distribution of mGluR5 availability in the healthy human brain measured by high-resolution [11C]ABP688 PET. Eur J Nucl Med Mol Imaging. 2016 Jan;43(1):152–62.*

*73.	Galovic M, Al-Diwani A, Vivekananda U, Torrealdea F, Erlandsson K, Fryer TD, et al. In vivo NMDA receptor function in people with NMDA receptor antibody encephalitis [Internet]. 2021 [cited 2025 Aug 5]. Available from: http://medrxiv.org/lookup/doi/10.1101/2021.12.04.21267226*

*74.	Galovic M, Erlandsson K, Fryer TD, Hong YT, Manavaki R, Sari H, et al. Validation of a combined image derived input function and venous sampling approach for the quantification of [18F]GE-179 PET binding in the brain. NeuroImage. 2021 Aug;237:118194.*

*75.	McGinnity CJ, Hammers A, Riaño Barros DA, Luthra SK, Jones PA, Trigg W, et al. Initial Evaluation of^18^ F-GE-179, a Putative PET Tracer for Activated N -Methyl d-Aspartate Receptors. J Nucl Med. 2014 Mar;55(3):423–30.*

*76.	Kantonen T, Karjalainen T, Isojärvi J, Nuutila P, Tuisku J, Rinne J, et al. Interindividual variability and lateralization of μ-opioid receptors in the human brain: Individual differences in the μ-opioid receptor system. NeuroImage. 2020;217(April).*

*77.	Turtonen O, Saarinen A, Nummenmaa L, Tuominen L, Tikka M, Armio RL, et al. Adult Attachment System Links With Brain Mu Opioid Receptor Availability In Vivo. Biological Psychiatry: Cognitive Neuroscience and Neuroimaging. 2021;6(3):360–9.*

*78.	Vijay A, Cavallo D, Goldberg A, De Laat B, Nabulsi N, Huang Y, et al. PET imaging reveals lower kappa opioid receptor availability in alcoholics but no effect of age. Neuropsychopharmacol. 2018 Dec;43(13):2539–47.*

*79.	Laurikainen H, Tuominen L, Tikka M, Merisaari H, Armio RL, Sormunen E, et al. Sex difference in brain CB1 receptor availability in man. NeuroImage. 2019 Jan;184:834–42.*

*80.	Normandin MD, Zheng MQ, Lin KS, Mason NS, Lin SF, Ropchan J, et al. Imaging the Cannabinoid CB1 Receptor in Humans with [^11^ C] OMAR: Assessment of Kinetic Analysis Methods, Test–Retest Reproducibility, and Gender Differences. J Cereb Blood Flow Metab. 2015 Aug;35(8):1313–22.*

*81.	Xu T, Nenning KH, Schwartz E, Hong SJ, Vogelstein JT, Goulas A, et al. Cross-species functional alignment reveals evolutionary hierarchy within the connectome. NeuroImage. 2020 Dec;223:117346.*

*82.	Sydnor VJ, Larsen B, Bassett DS, Alexander-Bloch A, Fair DA, Liston C, et al. Neurodevelopment of the association cortices: Patterns, mechanisms, and implications for psychopathology. Neuron. 2021 Sep;109(18):2820–46.*

*95.	Cotter DL, Ahmadi H, Cardenas-Iniguez C, Bottenhorn KL, Gauderman WJ, McConnell R, et al. Exposure to multiple ambient air pollutants changes white matter microstructure during early adolescence with sex-specific differences. Commun Med. 2024 Aug 1;4(1):155.*

*96.	Kim H, Noh J, Noh Y, Oh SS, Koh SB, Kim C. Gender Difference in the Effects of Outdoor Air Pollution on Cognitive Function Among Elderly in Korea. Front Public Health. 2019 Dec 10;7:375.*

*97.	Kuźma Ł, Struniawski K, Pogorzelski S, Bachórzewska-Gajewska H, Dobrzycki S. Gender Differences in Association between Air Pollution and Daily Mortality in the Capital of the Green Lungs of Poland–Population-Based Study with 2,953,000 Person-Years of Follow-Up. JCM. 2020 Jul 23;9(8):2351.*

*98.	Mo S, Wang Y, Peng M, Wang Q, Zheng H, Zhan Y, et al. Sex disparity in cognitive aging related to later-life exposure to ambient air pollution. Science of The Total Environment. 2023 Aug;886:163980.*

*99.	Oiamo T, Luginaah I. Extricating Sex and Gender in Air Pollution Research: A Community-Based Study on Cardinal Symptoms of Exposure. IJERPH. 2013 Aug 22;10(9):3801–17.*
